# Branched-chain amino acid transaminase 1–mediated pathway promotes proline-dependent collagen production in cardiac myofibroblasts

**DOI:** 10.1172/JCI182216

**Published:** 2026-07-16

**Authors:** Noburo Takizawa, Takanori Hironaka, Hayato Watanabe, Haruna Suetsugu, Keisuke Yoshioka, Yuma Horii, Yuri Nagata, Hiroaki Matoba, Hidetaka Kosako, Kenji Hamase, Go Hirai, Michio Nakaya

**Affiliations:** 1 Department of Disease Control, Graduate School of Pharmaceutical Sciences, Kyushu University, Fukuoka, Japan.; 2 Department of Disease Control, Research Institute of Environmental Medicine, Nagoya University, Nagoya, Japan.; 3 Department of Drug Discovery and Evolution, Graduate School of Pharmaceutical Sciences, and; 4 Department of Pharmaceutical Synthetic Chemistry, Graduate School of Pharmaceutical Sciences, Kyushu University, Fukuoka, Japan.; 5 Division of Cell Signaling, Fujii Memorial Institute of Medical Sciences, Institute of Advanced Medical Sciences, Tokushima University, Tokushima, Japan.

**Keywords:** Cardiology, Metabolism, Therapeutics, Amino acid metabolism, Cardiovascular disease, Fibrosis

## Abstract

Myofibroblasts are the cells responsible for collagen production, leading to tissue fibrosis. Because 20.5% of the total amino acids in collagen are proline, myofibroblasts must acquire a well-developed proline-producing mechanism during their differentiation. However, the detailed mechanism for myofibroblasts to acquire and keep the developed proline biosynthesis machinery remains obscure. Here, we show that branched-chain amino acid (BCAA) transaminase 1 (*Bcat1*) was upregulated in a substantial subset of Periostin (*Postn*)-expressing protomyofibroblast-like fibroblasts, which are transitional cells en route to fully differentiated myofibroblasts, as well as in myofibroblasts in the fibrotic hearts and livers of mice and humans, and promoted the production of proline. The production of BCAA by BCAT1 promoted SMAD3 phosphorylation via HDAC5 phosphorylation at Ser488, thereby enhancing SMAD3-dependent transcription of the proline biosynthesis–related genes *Aldh18a1*, *Pycr1*, and *Eprs* in protomyofibroblast-like fibroblasts and myofibroblasts. In BCAT1-deficient mice, expression of proline biosynthesis–related genes was significantly attenuated in their hearts after myocardial infarction (MI), resulting in decreased cardiac fibrosis. Moreover, mice with MI that were treated with a BCAT1 inhibitor had reduced cardiac fibrosis. Our results identified a BCAT1-mediated pathway that promoted collagen production via proline biosynthesis regulation in protomyofibroblast-like fibroblasts and myofibroblasts, which may provide a therapeutic target for cardiac fibrosis.

## Abstract 2

BCAT1 enhances cardiac fibrosis by promoting synthesis of proline, which constitutes about 20% of total amino acids in collagen.

## Graphical abstract

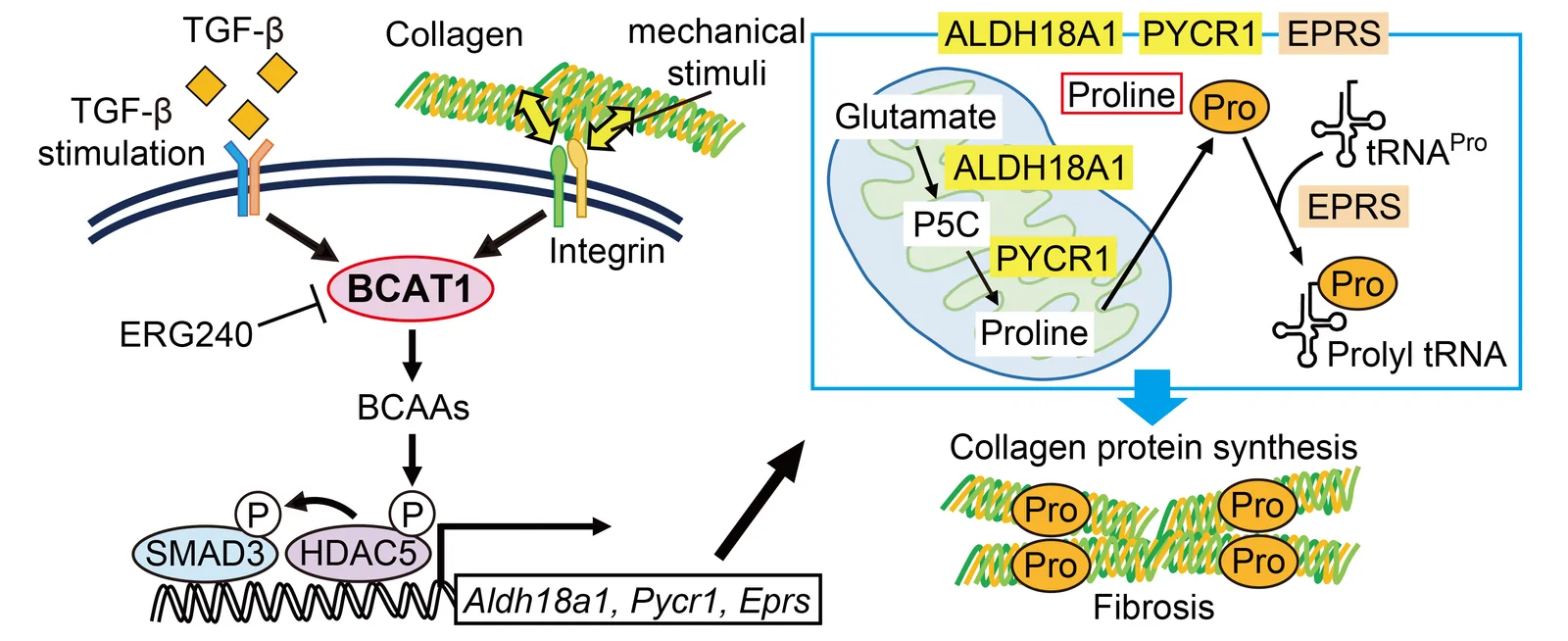

## Introduction

Fibrosis is a disease characterized by excessive deposition of extracellular matrix (ECM) components such as collagen in tissues as a result of inflammation and aging (1–3). Excessive ECM deposition results in tissue dysfunction (4). For example, when fibrosis progresses in the heart after myocardial infarction (MI) or in a liver diagnosed with metabolic dysfunction–associated steatohepatitis (MASH), heart failure or liver cirrhosis eventually occurs. Fibrosis is reportedly associated with approximately 45% of the deaths in developed countries (5). However, effective antifibrotic therapies remain limited, underscoring the need for therapeutic agents and treatments for fibrosis.

The cells that produce excessive ECM in fibrotic tissues are called myofibroblasts. These cells are absent from healthy tissues and appear only in fibrotic ones (3, 6, 7). Myofibroblasts mainly differentiate from resident fibroblasts after stimulation by humoral factors such as TGF-β released by macrophages during inflammation (8–10). Accumulating evidence suggests that this differentiation process is not a binary switch, but rather a continuum involving intermediate-state cells, such as protomyofibroblasts and protomyofibroblast-like fibroblasts, which exhibit partial activation of fibrogenic programs before full myofibroblast maturation (10–15). During this differentiation process, fibroblasts acquire the ability to produce and deposit excess ECM in the stroma, causing tissue hardening (10). Importantly, protomyofibroblasts, protomyofibroblast-like fibroblasts, and myofibroblasts, collectively referred to here as fibrogenic fibroblast–lineage cells, detect surrounding ECM stiffness via integrins and activate Rho/Rock signaling, forming an actin cytoskeleton, which then leads to further ECM production (16, 17).

Collagen, the main ECM protein found in fibrotic tissues, is characterized by a high proline content in its amino acid composition (18). Analysis by mass spectrometry (MS) has shown that proline constitutes approximately 20% of all amino acids in collagen (18). Accordingly, large amounts of proline must be produced by fibrogenic fibroblast–lineage cells to allow them to biosynthesize vast amounts of collagen. Proline is synthesized from glutamate in 2 steps (19, 20). First, glutamate is converted into pyrroline-5-carboxylate (P5C), which is catalyzed by aldehyde dehydrogenase 18 family member A1 (ALDH18A1) and subsequently yields proline via the enzymatic action of pyrroline-5-carboxylate reductase 1 (PYCR1) (19, 20). These enzymes are thus important for collagen production by myofibroblasts (21–23). In addition to de novo proline synthesis, glutamyl prolyl tRNA synthetase (EPRS), a prolyl-tRNA synthetase, promotes the production of collagen in both cardiac fibroblasts and fibrogenic fibroblast–lineage cells (24). However, the details of the molecular mechanisms underlying abundant proline production required for excessive collagen production in fibrogenic fibroblast–lineage cells remain unclear.

Two forms of branched-chain aminotransferases (BCATs) are present in mammals. BCAT1 is localized in the cytoplasm, whereas BCAT2 resides in the mitochondria (25, 26). Both BCATs are enzymes responsible for the production of branched-chain α-keto acids (BCKAs) and glutamic acid, by exchanging the amino group of branched-chain amino acids (BCAAs), such as valine, leucine, and isoleucine, for the keto group of α-ketoglutarate, or vice versa (25, 26). While BCAT2 is ubiquitously expressed in most tissues, BCAT1 expression is limited to embryonic tissues, adult brain, ovaries, and placental tissues, with low levels of expression. BCAT1 also reportedly promotes the production of glutamate neurotransmitters in the brain (27) as well tumor growth via mTOR signaling (28) and activates macrophages via oxygen consumption- and glycolysis-related mechanisms (29). However, its function in fibrogenic fibroblast–lineage cells remains unclear.

In this study, we revealed that BCAT1 was preferentially expressed in fibrogenic fibroblast–lineage cells, including a substantial subset of protomyofibroblast-like fibroblasts and myofibroblasts in fibrotic mouse hearts and livers, and promoted proline synthesis in fibrotic hearts, leading to enhanced collagen production and exacerbation of cardiac dysfunction.

## Results

### Mechanical stimulation induces Bcat1 expression in myofibroblasts.

**Figure 1:**
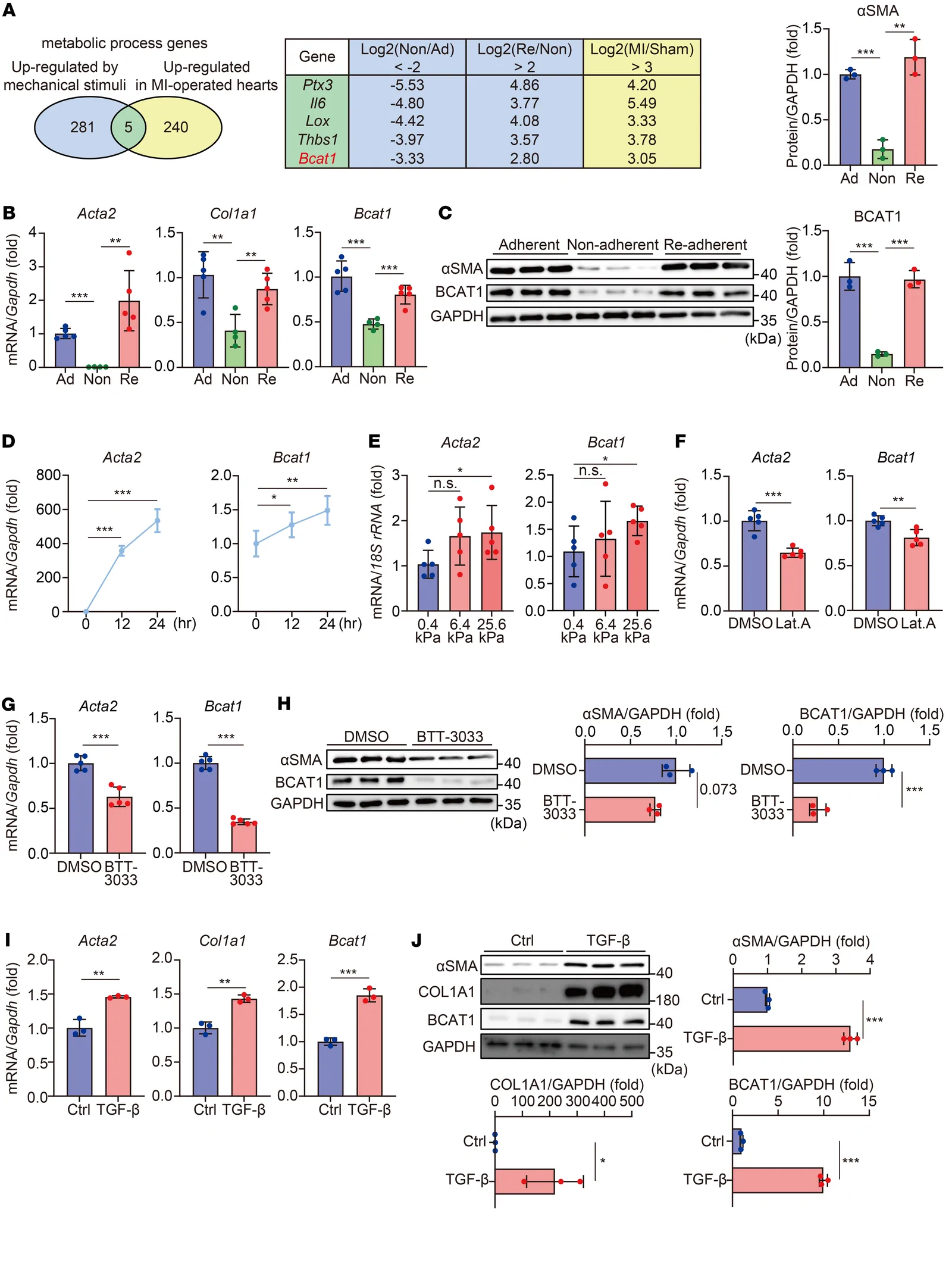
BCAT1 expression is induced in myofibroblasts by mechanical stimulation and TGF-β signaling. (**A**) Venn diagram of metabolic process genes (GO: 0008152) upregulated by mechanical stimulation and in MI-operated mouse hearts. Five overlapping genes are shown. (**B**) mRNA levels of *Acta2*, *Col1a1*, and *Bcat1* in adherent, nonadherent, or re-adherent cardiac myofibroblasts isolated from mouse hearts at post-MI day 3 (*n* = 4–5). (**C**) Protein levels of αSMA, BCAT1, and GAPDH in adherent, nonadherent, and re-adherent cardiac myofibroblasts (*n* = 3). (**D**) mRNA levels of *Acta2* and *Bcat1* at 0, 12, and 24 hours after re-adherence and in nonadherent cardiac myofibroblasts (*n* = 5). (**E**) mRNA levels of *Acta2* and *Bcat1* in NIH3T3 cells cultured on 0.4, 6.4, or 25.6 kPa stiffness gels for 12 hours (*n* = 5). (**F**) mRNA levels of *Acta2* and *Bcat1* in cardiac myofibroblasts after latrunculin A (Lat.A) treatment (*n* = 5). (**G**) mRNA levels of *Acta2* and *Bcat1* in cardiac myofibroblasts after BTT-3033 treatment (*n* = 5). (**H**) Protein levels of αSMA, BCAT1, and GAPDH in cardiac myofibroblasts after BTT-3033 treatment (*n* = 3). (**I** and **J**) mRNA levels of *Acta2*, *Col1a1*, and *Bcat1* (**I**) and protein levels of αSMA, BCAT1, and GAPDH (**J**) in cardiac resident fibroblasts isolated from normal mouse hearts after TGF-β stimulation (*n* = 3). An unpaired, 2-tailed *t* test was used for **B**–**J**. *P < 0.05, **P < 0.01, and ***P < 0.001. Ad, adherent; Non, nonadherent; Re, re-adherent. Data are shown as the mean ± SD.

We recently reported that culturing myofibroblasts isolated from fibrotic mouse hearts in suspension for 7 days, thereby removing mechanical stimulation, induces dedifferentiation (30) (Supplemental Figure 1A; supplemental material available online with this article; https://doi.org/10.1172/JCI182216DS1). Furthermore, we showed that adherent culture for 7 days redifferentiates these cells into myofibroblasts (30) (Supplemental Figure 1A). Myofibroblast differentiation strongly induces metabolic reprogramming (5). Thus, we identified the metabolic process–related genes (Gene Ontology [GO]: 0008152) that showed altered expression during differentiation by comparing gene expression in adherent, nonadherent, and re-adherent myofibroblasts (GSE158236) (Supplemental Figure 1A). We identified 286 metabolic process–related genes downregulated more than 4-fold in dedifferentiated myofibroblasts and upregulated more than 4-fold upon redifferentiation (Figure 1A). Among the 286 genes, five (*Ptx3*, *Il6*, *Lox*, *Thbs1*, and *Bcat1*) showed significantly increased expression (greater than 8-fold) in fibrotic mouse hearts 3 days after MI surgery (GSE158157) (Figure 1A). In this study, we focused on BCAT1 because its function in myofibroblasts remains unclear. Using quantitative reverse transcription PCR (qRT-PCR), we confirmed that the mRNA expression levels of *Bcat1* as well as of *Acta2* (which encodes α–smooth muscle actin [αSMA]) and *Col1a1* were decreased in nonadherent myofibroblasts and were increased in re-adherent myofibroblasts (Figure 1B) (*Acta2* and *Col1a1* are myofibroblast markers). Consistently, BCAT1 and αSMA protein levels were also decreased in nonadherent myofibroblasts and increased in re-adherent myofibroblasts (Figure 1C).

Next, we examined temporal changes in *Bcat1* mRNA expression in nonadherent myofibroblasts after transfer to plastic plates for mechanical stimulation. *Bcat1* and *Acta2* mRNA levels increased after mechanical stimulation in nonadherent myofibroblasts (Figure 1D). To test whether *Bcat1* mRNA expression is stiffness regulated, mouse embryonic fibroblasts were cultured on polyacrylamide gels of defined stiffness (0.4, 6.4, or 25.6 kPa). We found that *Bcat1* mRNA expression was significantly increased on the stiffest substrate (25.6 kPa), together with expression of the mechanosensitive genes *Acta2*, *Ctgf*, and *Cyr61* (Figure 1E and Supplemental Figure 1B).

The actin cytoskeleton mediates mechanical stimulation (31, 32). Thus, we examined the effect of latrunculin A, an inhibitor of actin cytoskeleton formation, on *Bcat1* mRNA expression in myofibroblasts. Latrunculin A treatment significantly reduced mRNA expression of *Bcat1* and *Acta2* (Figure 1F). Integrin β1 in myofibroblasts senses mechanical stimulation from the surrounding accumulated collagen, activating Rho/Rock signaling and promotes actin cytoskeleton formation (16, 17). Treatment with the integrin inhibitor (BTT-3033) decreased *Acta2* and *Bcat1* mRNA expression (Figure 1G), and αSMA and BCAT1 protein levels (Figure 1H). These results indicated that BCAT1 expression in myofibroblasts was induced by mechanical stimulation.

Myocardin-related transcription factors A/B (MRTFA/B) (33) and yes-associated protein 1 (YAP1) (34) are well-established mechanosensitive transcriptional coregulators. We therefore examined whether they are involved in the induction of *Bcat1* mRNA expression by mechanical stimulation. In myofibroblasts, knockdown of *Mrtfa/b* or *Yap1* did not affect *Bcat1* mRNA expression (Supplemental Figure 1, C and D).

Taken together, these results indicate that mechanical induction of *Bcat1* occurred independently of MRTFs and YAP1, suggesting the presence of an alternative regulatory mechanism.

### TGF-β signaling induces Bcat1 expression in myofibroblasts.

In addition to mechanical stimulation, TGF-β signaling is important for myofibroblast differentiation (10–12). Thus, we examined whether BCAT1 expression could be induced by TGF-β signaling.

Resident fibroblasts from normal mouse hearts and fibroblast cell lines such as IMR-90 differentiate into myofibroblasts and myofibroblast-like cells, respectively, upon TGF-β stimulation and thus increase their collagen production capacity (35, 36). As reported previously (30, 37), we found that mRNA expression of *Acta2* and *Col1a1* in cardiac resident fibroblasts and IMR-90 cells was increased by TGF-β treatment (Figure 1I and Supplemental Figure 1E), suggesting that these were myofibroblasts and myofibroblast-like cells, respectively. Under these conditions, TGF-β treatment increased *Bcat1* expression (Figure 1I and Supplemental Figure 1E). We further confirmed that TGF-β stimulation increased the protein levels of αSMA, collagen type I alpha 1 chain (COL1A1), and BCAT1 in cardiac resident fibroblasts (Figure 1J). In contrast, stimulation with TNF-α or LPS, both of which elicit the inflammatory signaling, did not increase *Bcat1* in cardiac resident fibroblasts (Supplemental Figure 1, F and G).

BCAT1 mediates BCAA catabolism (25, 26). BCAT1, in the cytoplasm, or BCAT2, in the mitochondria, converts BCAAs, such as valine, leucine, and isoleucine, to BCKAs (Supplemental Figure 1H). The BCKA dehydrogenase (BCKDH) complex, mainly BCKDHA and BCKDHB, then degrades BCKAs in mitochondria, generating products that enter the TCA cycle (Supplemental Figure 1H). We therefore examined the mRNA expression levels of *Bcat2*, *Bckdha*, and *Bckdhb* in cardiac resident fibroblasts after TGF-β stimulation. We found that TGF-β stimulation did not increase these mRNA expression levels (Supplemental Figure 1I). In adherent, nonadherent, and re-adherent myofibroblasts, *Bcat2*, *Bckdha*, and *Bckdhb* showed a pattern opposite that seen with *Bcat1*, with higher expression in nonadherent and lower expression in adherent and re-adherent myofibroblasts (Figure 1B and Supplemental Figure 1J).

These results indicated that TGF-β signaling induced the expression of *Bcat1,* but not that of other BCAA metabolism–related genes, in myofibroblasts.

### MI induces Bcat1 expression in protomyofibroblast-like fibroblasts and myofibroblasts.

**Figure 2:**
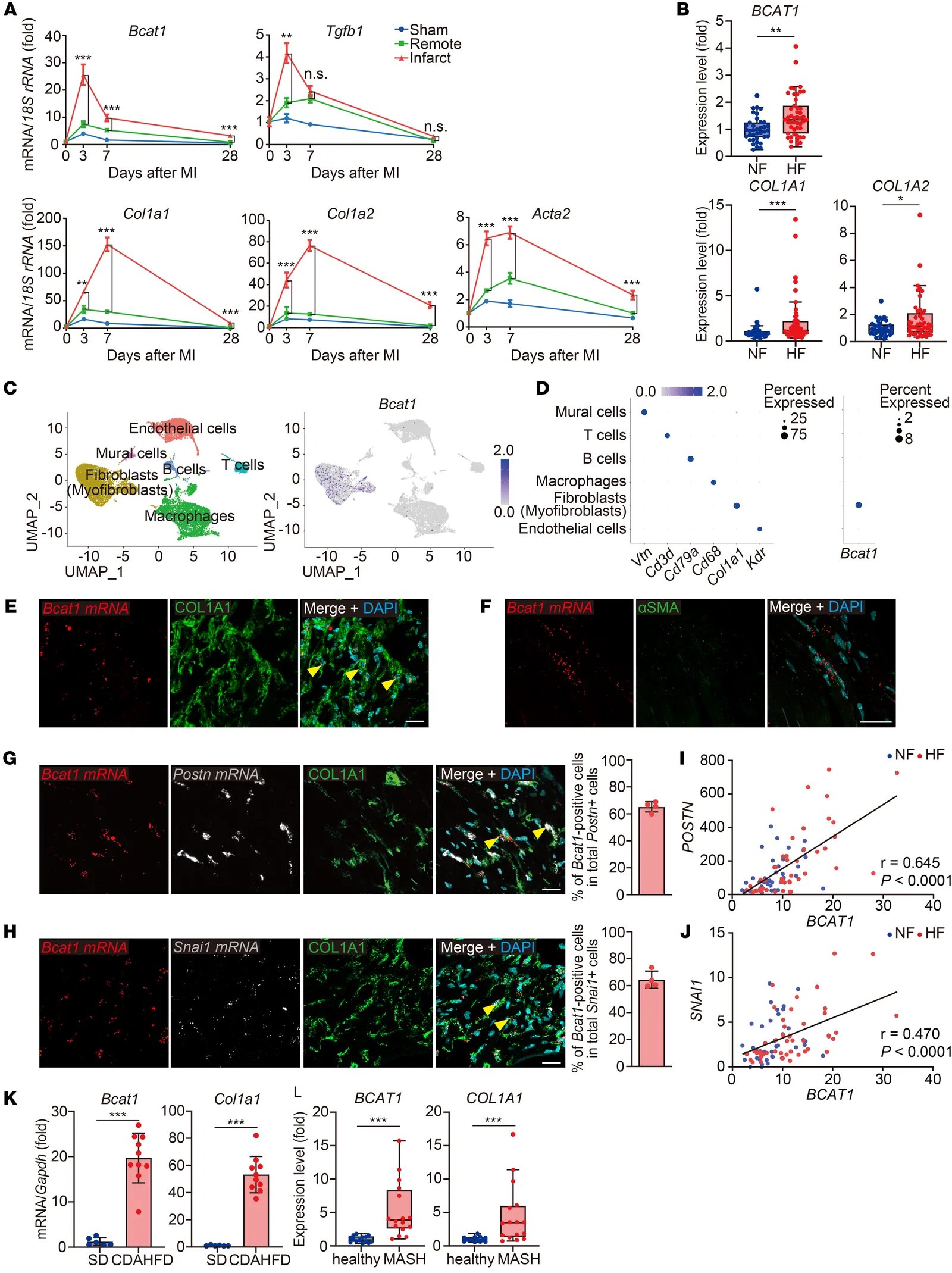
*Bcat1* expression is specifically induced in a substantial subset of protomyofibroblast-like fibroblasts of mammalian fibrotic hearts and livers. (**A**) mRNA levels of *Bcat1*, *Tgfb1*, *Acta2*, *Col1a1*, and *Col1a2* in intact, sham-operated, and MI-operated mouse hearts. The MI-operated mouse hearts were divided into infarcted (Infarct) and noninfarcted (Remote) areas (*n* = 3–6). (**B**) Expression levels of *BCAT1*, *COL1A1*, and *COL1A2* in nonfailing (NF) human hearts or human HF hearts (GSE161472, NF: *n* = 37, HF: *n* = 47). (**C**) Uniform manifold approximation and projection (UMAP) plot of the scRNA-seq dataset E-MTAB-7376 with annotation by cell type (left) and *Bcat1* expression in stromal cells from mouse hearts, 3 and 7 days after sham treatment or MI (right). (**D**) Dot plot of marker genes in each cell (left) and *Bcat1* expression (right). (**E** and **F**) Codetection of *Bcat1* mRNA and COL1A1 (**E**) or αSMA (**F**) protein in mouse hearts 3 days after MI. Scale bar: 20 μm. (**G** and **H**) Codetection of *Bcat1* mRNA, COL1A1 protein, and *Postn* mRNA (**G**), or *Snai1* mRNA (**H**) in mouse hearts 3 days after MI. Each graph shows the percentages of *Bcat1*^+^ cells in *Postn*^+^ or *Snai1*^+^ cells of the infarcted area (*n* = 4). Scale bars: 20 μm. (**I** and **J**) Relative *BCAT1* and *POSTN* (**I**) or *SNAI1* (**J**) in NF (blue) or HF (red) human hearts (GSE161472, NF: *n* = 37, HF: *n* = 47). (**K**) mRNA levels of *Bcat1* and *Col1a1* in livers of mice fed a standard diet or a CDAHFD (standard diet: *n* = 6, CDAHFD: *n* = 10). (**L**) BCAT1 and COL1A1 expression in healthy and MASH human livers (GSE126848, healthy: *n* = 14, MASH: *n* = 16). An unpaired, 2-tailed *t* test was used for **A**, **B**, **K**, and **L**. Spearman’s rank correlation test was used for **I** and **J**. Arrowheads in the images indicate merged cells. *P < 0.05, **P < 0.01, and ***P < 0.001. Data are shown as the mean ± SD.

We next measured the mRNA expression of *Bcat1* and fibrosis-related genes in mouse hearts after MI. Expression of *Bcat1* and *Tgfb1* peaked at day 3 after MI, whereas *Col1a1*, *Col1a2*, and *Acta2* expression peaked at day 7 (Figure 2A). In contrast, other BCAA catabolism–related genes, including *Bcat2*, *Bckdha*, and *Bckdhb*, were not upregulated in the infarcted area (Supplemental Figure 2A). Notably, *Bcat1* expression remained elevated 28 days after MI, whereas *Tgfb1* expression had returned to basal levels by this time point (Figure 2A). Thus, at this phase, *Bcat1* expression appeared more closely associated with collagen-derived mechanical cues than was observed with *Tgfb1* expression.

We also analyzed *BCAT1* expression in hearts from healthy volunteers and patients with heart failure (HF) with reduced ejection fraction (HFrEF) (GSE161472). The expression of *BCAT1*, *COL1A1*, and *COL1A2* was significantly increased in the hearts of patients with HFrEF (Figure 2B). In contrast, the expression levels of *Bcat2*, *Bckdha*, and *Bckdhb* did not increase in the hearts of patients with HFrEF (Supplemental Figure 2B).

To identify cells expressing *Bcat1* in fibrotic hearts after MI, we analyzed a published single-cell RNA-seq (scRNA-seq) dataset derived from stromal cells in mouse hearts 3 or 7 days after sham treatment or MI (E-MTAB-7376). In scRNA-seq analysis, stromal cells were divided into 6 clusters on the basis of marker gene expression: fibroblasts (e.g., *Col1a1*), endothelial cells (e.g., *Kdr*), macrophages (e.g., *Cd68*), B cells (e.g., *Cd79a*), T cells (e.g., *Cd3d*), and mural cells (e.g., *Vtn*) (Figure 2C, Supplemental Figure 2C). *Bcat1* was specifically expressed in a cluster of fibroblasts, including myofibroblasts (Figure 2C). Dot plots also showed that the expression of *Bcat1* was specific to a cluster of fibroblasts, including myofibroblasts (Figure 2D). BCAT1 has been reported to be expressed in human monocyte–derived macrophages, where it promotes immune-responsive gene 1 (IRG1) expression and enhances macrophage infiltration and collagen accumulation in arthritis and glomerulonephritis (29). In contrast, *Irg1* was not expressed in the (myo)fibroblast cluster in post-MI hearts (Supplemental Figure 2C), and *Bcat1* expression was not detected in CD45^+^ cells isolated from fibrotic mouse hearts at day 3 after MI (Supplemental Figure 2D). Consistently, at day 3 after MI, *Bcat1* mRNA was detected in fibroblasts expressing abundant COL1A1, but not in CD45^+^ leukocytes, CD31^+^ endothelial cells, or TNNI3^+^ cardiomyocytes (Figure 2E and Supplemental Figure 2, E–G). *Bcat1* mRNA was also nearly absent in sham-operated hearts, indicating that it was not expressed in resident fibroblasts (Supplemental Figure 2H). Notably, *Bcat1* mRNA was detected in αSMA^–^ fibroblasts at day 3 after MI (Figure 2F), suggesting that *Bcat1* expression was induced before the emergence of fully differentiated αSMA^+^ myofibroblasts. We therefore referred to these COL1A1^hi^, αSMA^–^ fibroblasts at day 3 after MI as protomyofibroblast-like fibroblasts. At day 7 after MI, abundant αSMA^+^ myofibroblasts were present in the infarcted hearts (Supplemental Figure 2I). Previous studies have identified Periostin (*Postn*) as a marker of protomyofibroblasts (13) and Snail family transcriptional repressor 1 (*Snai1*) as an early regulator of fibroblast-to-myofibroblast transition (14, 15). Therefore, we examined the relationship between *Bcat1* mRNA expression and *Postn*- or *Snai1*-expressing cells at day 3 after MI. IHC and ISH analyses showed that a substantial proportion of *Postn*-expressing cells (approximately 65%) and *Snai1*-expressing cells (approximately 64%) expressed *Bcat1* mRNA (Figure 2, G and H). Consistent with these findings, *BCAT1* expression positively correlated with *POSTN* and *SNAI1* expression in human HFrEF hearts (Figure 2, I and J). *Bcat1* mRNA expression was induced in a substantial subset of *Postn*- or *Snai1*-expressing protomyofibroblast-like fibroblasts and was still detectable in αSMA^+^ myofibroblasts at day 7 after MI (Supplemental Figure 2I). Together, these findings suggest that *Bcat1* expression was induced in a substantial subset of protomyofibroblast-like fibroblasts during early myofibroblast differentiation and remained expressed in αSMA^+^ myofibroblasts after MI (Supplemental Figure 2J).

### Bcat1 expression is specifically induced in myofibroblasts of fibrotic livers.

We investigated whether fibrosis increased *Bcat1* mRNA expression in mouse livers. Mice were fed a choline-deficient, methionine-reduced high-fat diet (CDAHFD) for 10 weeks to induce MASH, which progresses to liver fibrosis. The mRNA expression levels of *Bcat1* and *Col1a1* were markedly increased in the liver (Figure 2K). Analysis of the RNA-seq dataset (GSE126848) demonstrated that expression levels of *BCAT1* and *COL1A1* were also increased in the livers of patients with MASH (Figure 2L).

IHC analysis of mouse livers with MASH demonstrated that *Bcat1* mRNA was specifically expressed in activated hepatic stellate cells (HSCs), corresponding to liver myofibroblasts characterized by both desmin and abundant *Col1a1* mRNA expression (Supplemental Figure 2K).

### The expression levels of Aldh18a1, Pycr1, and Eprs are enriched in Bcat1^+^ fibrogenic fibroblast–lineage cells.

**Figure 3:**
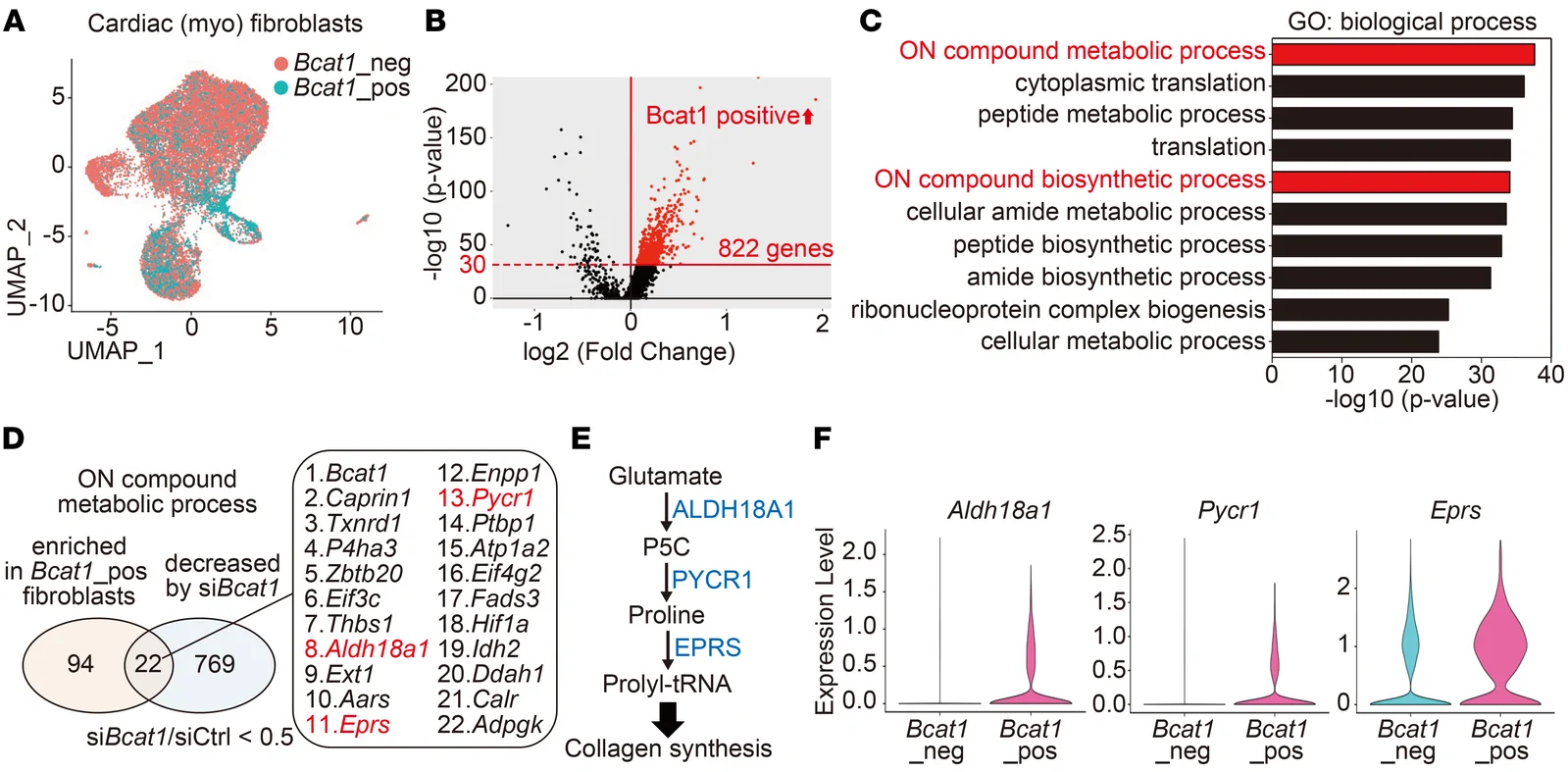
*Aldh18a1*, *Pycr1*, and *Eprs* expression is enriched in fibrogenic fibroblast–lineage cells and induced by BCAT1. (**A**) UMAP plot of *Bcat1*^–^ (*Bcat1*_neg) and *Bcat1^+^* (*Bcat1*_pos) fibroblasts isolated from mouse hearts 3 and 7 days after sham treatment or MI (E-MTAB-7376). (**B**) Volcano plot for comparison of gene expression between *Bcat1^–^* and *Bcat1^+^* fibroblasts. (**C**) DAVID analysis of enriched genes in *Bcat1^+^* fibroblasts based on log_2_ (fold change) and –log_10_ (*P* value), shown as red dots in **B**. (**D**) Venn diagram of ON compound metabolic process–related genes (GO: 1901564) that are highly expressed in *Bcat1^+^* fibroblasts according to scRNA-seq and that were downregulated by *Bcat1* knockdown in protomyofibroblast-like fibroblasts isolated from mouse hearts 3 days after MI according to RNA-seq. (**E**) Schematic diagram of proline and collagen synthesis from glutamate. (**F**) Expression levels of *Aldh18a1*, *Pycr1*, and *Eprs* in *Bcat1^–^* or *Bcat1^+^* fibroblasts.

To investigate the role of *Bcat1* in cardiac fibrogenic fibroblast–lineage cells, we analyzed a scRNA-seq dataset focusing on all fibroblasts, including resident fibroblasts and fibrogenic fibroblast–lineage cells in mouse hearts 3 and 7 days after sham treatment or MI (European Molecular Biology Laboratory – European Bioinformatics Institute [EMBL-EBI] accession code: E-MTAB-7376). We divided the fibroblasts into *Bcat1*^+^ and *Bcat1*^–^ fibroblasts (Figure 3A). The genes with differential expression between *Bcat1*^+^ and *Bcat1*^–^ fibroblasts are shown in a volcano plot in Figure 3B. We then compared gene expression in the 2-cell population and performed GO analysis using the Database for Annotation, Visualization, and Integrated Discovery (DAVID), focusing on 822 genes that showed abundant expression in *Bcat1*^+^ cells in Figure 3B. The analysis revealed that the genes related to organonitrogen (ON) compound metabolic or biosynthetic processes were enriched in *Bcat1*^+^ cells (Figure 3C). Of the 116 genes related to the organonitrogen (ON) compound metabolic process shown in Figure 3C, expression levels of 22 genes were greatly decreased by *Bcat1* knockdown in cardiac protomyofibroblast–like fibroblasts isolated from fibrotic mouse hearts 3 days after MI (si*Bcat1*/siControl [siCtrl] <0.5) (Figure 3D). Among these 22 genes, we identified the *Aldh18a1* and *Pycr1*, genes encoding proline synthesis enzymes and a proline translation enzyme gene, *Eprs* (Figure 3D). Glutamate is converted to proline by the sequential action of ALDH18A1 and PYCR1 (Figure 3E). The generated proline is attached to prolyl-tRNA via EPRS (Figure 3E), which promotes the production of proline-containing proteins, such as collagen (24). Using violin plots, we confirmed that the expression levels of *Aldh18a1*, *Pycr1*, and *Eprs* were higher in *Bcat1*^+^ cells than in *Bcat1*^–^ cells (Figure 3F).

### Mechanical stimulation and TGF-β signaling induce Aldh18a1, Pycr1, and Eprs expression.

**Figure 4:**
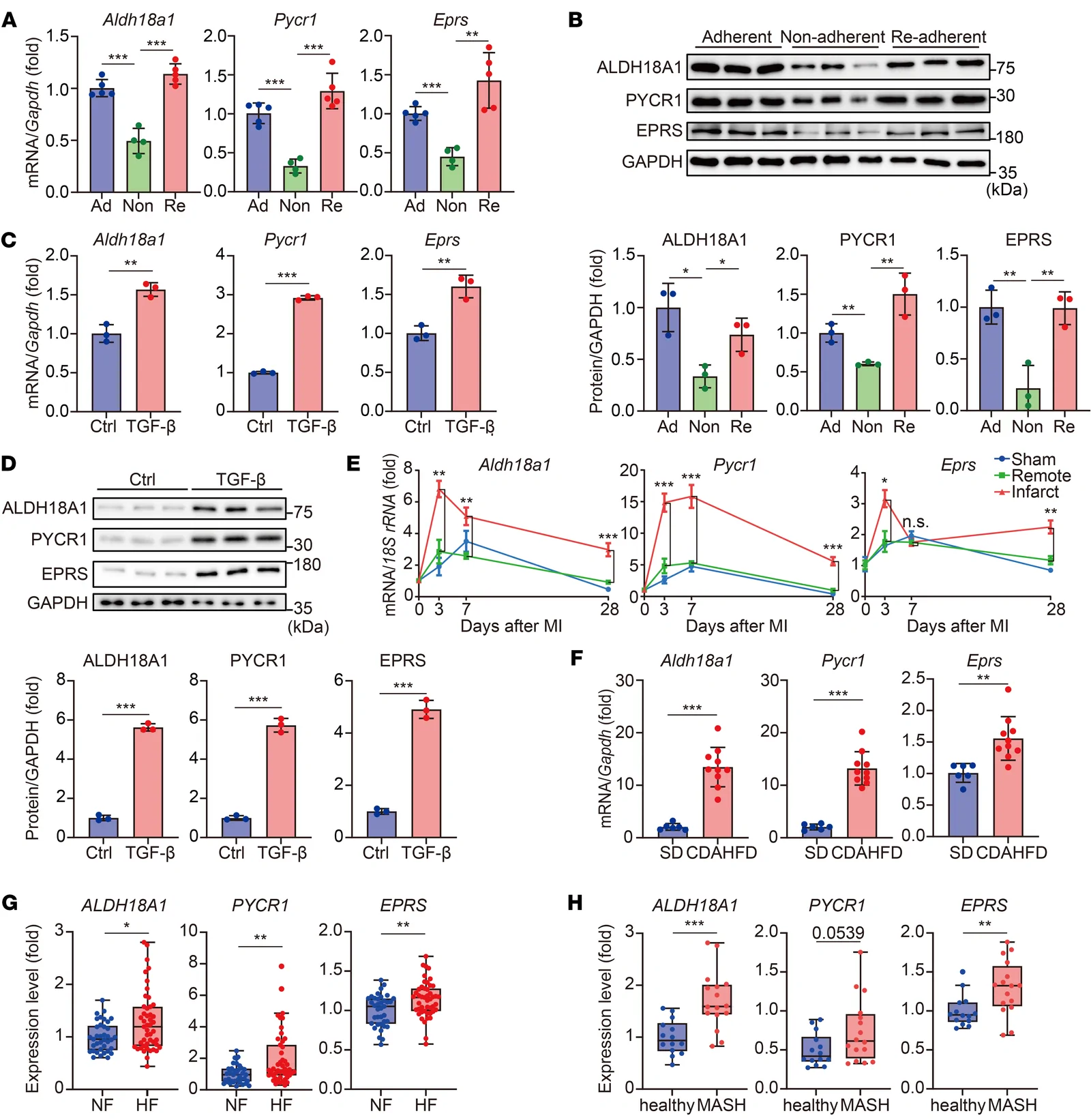
*Aldh18a1*, *Pycr1*, and *Eprs* expression is induced by mechanical stimulation and TGF-β signaling and is increased in mammalian fibrotic hearts and livers. (**A**) mRNA levels of *Aldh18a1*, *Pycr1*, and *Eprs* in adherent, nonadherent, or re-adherent cardiac myofibroblasts (*n* = 4–5). (**B**) Protein levels of ALDH18A1, PYCR1, EPRS, and GAPDH in adherent, nonadherent, or re-adherent cardiac myofibroblasts (*n* = 3). (**C**) mRNA levels of *Aldh18a1*, *Pycr1*, and *Eprs* in cardiac resident fibroblasts after TGF-β stimulation (*n* = 3). (**D**) Protein levels of ALDH18A1, PYCR1, EPRS, and GAPDH in cardiac resident fibroblasts after TGF-β stimulation (*n* = 3). (**E** and **F**) mRNA levels of *Aldh18a1*, *Pycr1*, and *Eprs* in intact mouse hearts or infarcted (infarct) and noninfarcted (remote) areas of mouse hearts 3, 7, and 28 days after sham treatment or MI (*n* = 3–6) (**E**) and mRNA levels in livers of mice fed a CDAHFD (standard diet: *n* = 6, CDAHFD: *n* = 10) (**F**). (**G** and **H**) Expression levels of *ALDH18A1*, *PYCR1*, and *EPRS* in human HF or NF hearts (GSE161472, NF: *n* = 37, HF: *n* = 47) (**G**) or human livers with MASH (GSE126848, healthy: *n* = 14, MASH: *n* = 16) (**H**). An unpaired, 2-tailed *t* test was used for **A**–**H**. *P < 0.05, **P < 0.01, and ***P < 0.001. Data represent the mean ± SD.

We next investigated whether mechanical stimulation and TGF-β signaling regulated the mRNA and protein expression of ALDH18A1, PYCR1, EPRS, and BCAT1 (Figure 1, B, C, I, and J). qRT-PCR and Western blot experiments demonstrated that all expression levels in nonadherent myofibroblasts were lower than those in adherent and re-adherent myofibroblasts (Figure 4, A and B), and TGF-β stimulation induced their expression in cardiac resident fibroblasts (Figure 4, C and D). These results indicated that both mechanical stimulation and TGF-β signaling induced the expression of *Aldh18a1*, *Pycr1*, and *Eprs* in myofibroblasts, as observed for *Bcat1*.

### Aldh18a1, Pycr1, and Eprs expression is increased in mouse hearts after MI and in livers of mice with MASH.

We determined the mRNA expression levels of *Aldh18a1*, *Pycr1*, and *Eprs* in mouse hearts after MI. Similar to *Bcat1*, the expression of these genes peaked on day 3 after MI (Figure 2A and Figure 4E). scRNA-seq revealed that *Aldh18a1* and *Pycr1* were specifically expressed in fibroblasts, including fibrogenic fibroblast–lineage cells, whereas *Eprs* was broadly expressed across multiple cell types in fibrotic hearts, including these cells, endothelial cells, and macrophages (Supplemental Figure 3). We also found that the mRNA expression levels of *Aldh18a1*, *Pycr1*, and *Eprs* were markedly increased in the livers of MASH model mice (Figure 4F).

Consistent with these data, the expression of *ALDH18A1*, *PYCR1*, and *EPRS* was increased in patients with HFrEF or MASH (GSE161472, GSE126848) (Figure 4, G and H). These results suggested that the expression levels of *Aldh18a1*, *Pycr1*, *Eprs*, and *Bcat1* were increased during fibrosis in both the heart and liver.

### BCAT1 promotes the expression of Aldh18a1, Pycr1, and Eprs.

**Figure 5:**
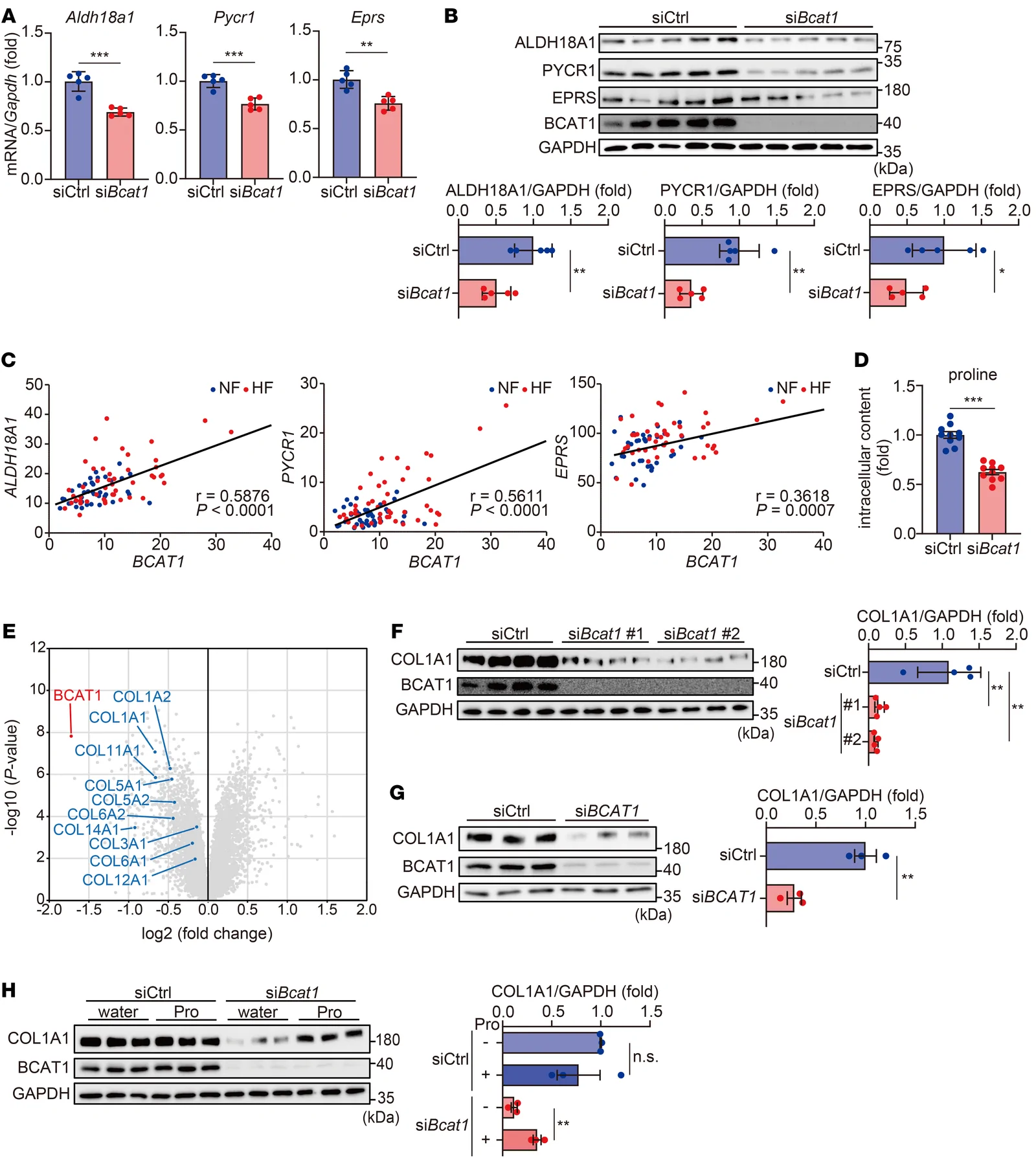
BCAT1 increases protein levels of collagen via proline biosynthesis in protomyofibroblast-like fibroblasts. (**A** and **B**) mRNA levels of *Aldh18a1*, *Pycr1*, and *Eprs* (**A**) and protein levels of ALDH18A1, PYCR1, EPRS, BCAT1, and GAPDH (**B**) in protomyofibroblast-like fibroblasts after si*Bcat1* treatment (*n* = 5). (**C**) Relative *BCAT1* and *ALDH18A1*, *PYCR1*, and *EPRS* mRNA expression in human HF hearts (GSE161472, NF: *n* = 37, HF: *n* = 47). (**D**) Intracellular proline levels in protomyofibroblasts after si*Bcat1* treatment (*n* = 10). (**E**) Volcano plot of the differential 7,672 proteins identified by LC-MS/MS in protomyofibroblast-like fibroblasts after si*Bcat1* treatment (*n* = 4). Blue dots represent the collagen proteins with the top-10 PSM values detected in these cells after si*Bcat1* treatment. (**F**) Protein levels of COL1A1, BCAT1, and GAPDH in protomyofibroblast-like fibroblasts after si*Bcat1* treatment (*n* = 5). (**G**) Protein levels of COL1A1, BCAT1, and GAPDH in CCD-18Co cells after si*BCAT1* treatment (*n* = 3). (**H**) Protein levels of COL1A1, BCAT1, and GAPDH in si*Bcat1*-treated protomyofibroblast-like fibroblasts after proline supplementation (*n* = 3). An unpaired, 2-tailed *t* test was used for **A**, **B**, and **D**–**H**. Spearman’s rank correlation test was used for **C**. *P < 0.05, **P < 0.01, and ***P < 0.001. Data represent the mean ± SD.

RNA-seq data (Figure 3D) suggested that the mRNA expression levels of *Aldh18a1*, *Pycr1*, and *Eprs* were reduced following *Bcat1* knockdown in cardiac protomyofibroblast–like fibroblasts. This was confirmed in protomyofibroblast-like fibroblasts isolated from fibrotic hearts and activated HSCs of fibrotic livers by qRT-PCR (Figure 5A and Supplemental Figure 4A). We further found that the protein expression levels of ALDH18A1, PYCR1, and EPRS were reduced by *Bcat1* knockdown in cardiac protomyofibroblast–like fibroblasts, whereas expression of αSMA was not, indicating that BCAT1 did not significantly affect differentiation into myofibroblasts (Figure 5B and Supplemental Figure 4B). Importantly, mRNA expression of *ALDH18A1*, *PYCR1*, and *EPRS* correlated with that of *BCAT1* in the hearts of patients with HFrEF (Figure 5C).

Because both ALDH18A1 and PYCR1 are enzymes that promote proline production (Figure 3E), we examined the amount of proline in protomyofibroblast-like fibroblasts treated with siRNA against *Bcat1*. 3D-HPLC analysis showed that si*Bcat1* treatment significantly decreased intracellular proline levels in protomyofibroblast-like fibroblasts, indicating that BCAT1 enhanced proline synthesis via ALDH18A1 and PYCR1 expression (Figure 5D and Supplemental Data 1). To assess global amino acid metabolic changes regulated by BCAT1, we performed comprehensive hydrophilic metabolomics in cardiac protomyofibroblast–like fibroblasts after *Bcat1* knockdown. Analysis of all 20 proteinogenic amino acids showed that *Bcat1* loss broadly altered intracellular amino acid levels (Supplemental Figure 4C and Supplemental Data 2). Consistent with previous findings in BCAT1^hi^ chronic myeloid leukemia cells showing increased levels of BCAAs, proline, and other amino acids (28), we observed the opposite pattern in cardiac protomyofibroblast–like fibroblasts, where these amino acids were consistently reduced after *Bcat1* silencing (Supplemental Figure 4C and Supplemental Data 2). Together, these findings suggest that *Bcat1* contributes to the regulation of intracellular amino acid pools across distinct cellular contexts.

### BCAT1 promotes the protein expression level of COL1A1.

ALDH18A1, PYCR1, and EPRS have been reported to increase collagen protein expression levels (21, 22, 24). Consistent with previous reports, the protein expression level of COL1A1 in protomyofibroblast-like fibroblasts was markedly suppressed by knockdown of *Aldh18a1*, *Pycr1*, or *Eprs* (Supplemental Figure 4, D–F). We confirmed that COL1A1 protein expression in protomyofibroblast-like fibroblasts was markedly suppressed by deficiency in glutamine and glutamic acid, which are upstream of proline synthesis (Supplemental Figure 4G).

Because we found that BCAT1 regulates the expression of ALDH18A1, PYCR1, and EPRS, we examined the effect of BCAT1 deficiency on the expression of collagen proteins, which contain a substantial amount of proline. Tandem mass tag–based (TMT-based) quantitative proteomics revealed that *Bcat1* knockdown in protomyofibroblast-like fibroblasts decreased protein expression of the top-10 collagens (COL14A1, COL1A1, COL11A1, COL1A2, COL5A1, COL6A2, COL5A2, COL6A1, COL12A1, and COL3A1) with the highest number of peptide spectrum matches (PSMs) in the MS analysis (Figure 5E and Supplemental Data 3). Among the collagens, COL1A1 was most abundant in fibrotic hearts, and we performed Western blotting using COL1A1 antibody to confirm the proteomics analysis. The experiments revealed that *Bcat1* knockdown decreased the protein expression levels of COL1A1 in protomyofibroblast-like fibroblasts and activated HSCs (Figure 5F and Supplemental Figure 4H). A decrease in COL1A1 protein expression levels after *BCAT1* knockdown was also observed in CCD-18Co cells, a human intestinal myofibroblast line, and in LX-2 cells, a human liver myofibroblast line (Figure 5G and Supplemental Figure 4I). Importantly, we also found that the decrease in protein expression levels of COL1A1 (containing 19.0% proline) after *Bcat1* knockdown in protomyofibroblast-like fibroblasts was rescued by the addition of proline (Figure 5H). In contrast, protein expression levels of COL6A1, a collagen with a lower proline content (9%), was not restored by proline supplementation (Supplemental Figure 4J). These findings raise the possibility that proline-dependent enhancement of protein synthesis in protomyofibroblast-like fibroblasts selectively augments collagens with a high proline content, such as COL1A1.

These results indicated that BCAT1 promoted the expression of COL1A1 protein, which has a high proline content, via both proline synthesis by ALDH18A1 and PYCR1 and proline attachment to its translation enzyme by EPRS in protomyofibroblast-like fibroblasts.

### BCAT1 promotes SMAD3 phosphorylation, which induces the expression of Aldh18a1, Pycr1, and Eprs.

We next investigated how BCAT1 promotes the expression of *Aldh18a1*, *Pycr1*, and *Eprs*. Because BCAT1 is an aminotransferase and has been reported to enhance mTOR signaling, an amino acid–sensing pathway (26), we examined the possible involvement of amino acid–responsive pathways. mTOR/transcription factor EB (TFEB) and General control nonderepressible 2 (GCN2) are well-established amino acid–responsive pathways (38, 39), we assessed the phosphorylation levels of mTOR, TFEB, and GCN2 following *Bcat1* knockdown in protomyofibroblast-like fibroblasts from mouse fibrotic hearts. However, Western blot analysis showed that the phosphorylation levels of mTOR, TFEB, and GCN2 were unchanged (Supplemental Figure 5A).

**Figure 6:**
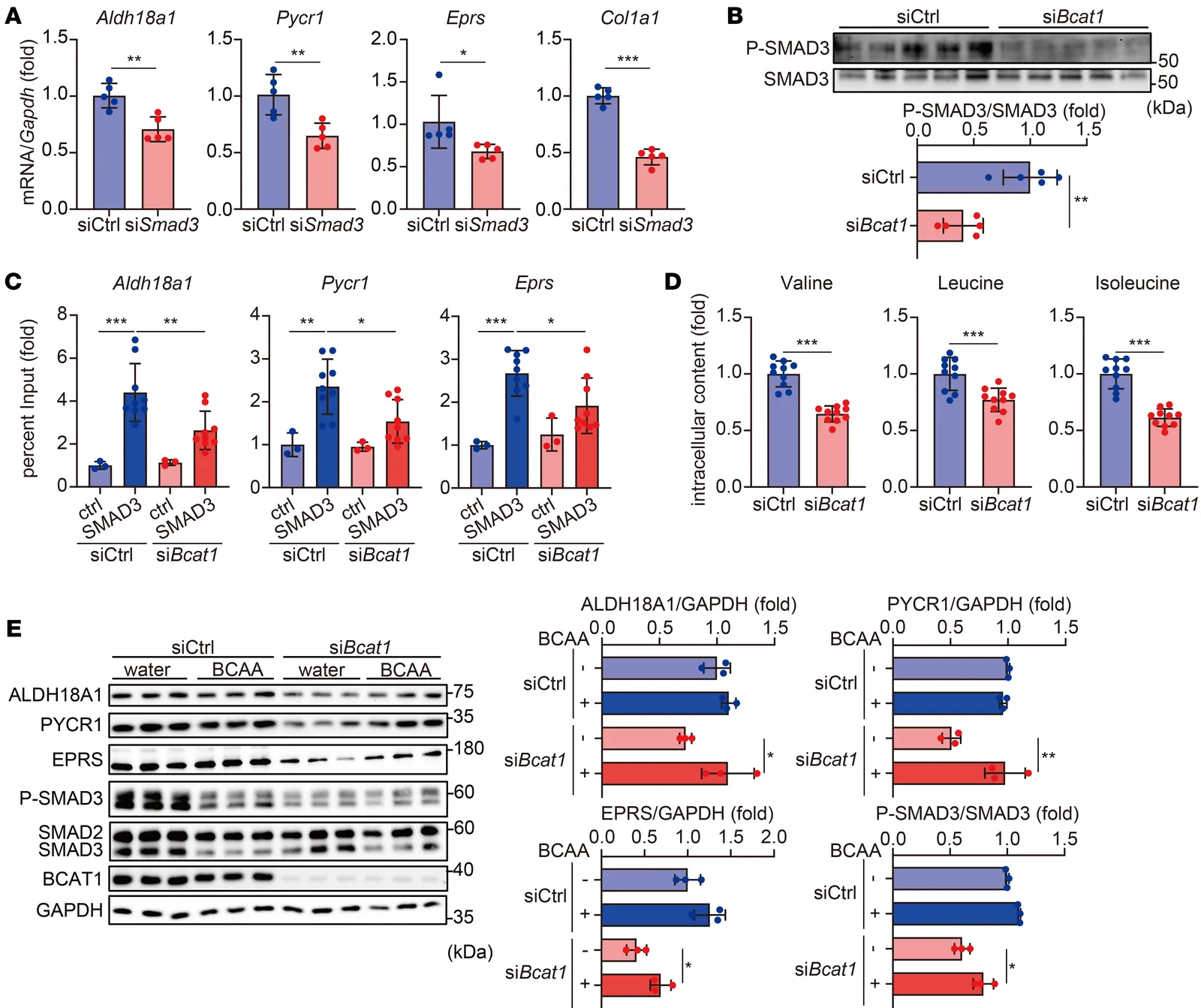
BCAT1 increases the expression levels of *Aldh18a1*, *Pycr1*, and *Eprs* in protomyofibroblast-like fibroblasts through BCAA-dependent SMAD3 signaling. (**A**) mRNA levels of *Aldh18a1*, *Pycr1*, *Eprs*, and *Col1a1* in protomyofibroblast-like fibroblasts after si*Smad3* treatment (*n* = 5). (**B**) Protein levels of p-SMAD3 and SMAD3 in protomyofibroblast-like fibroblasts after si*Bcat1* treatment (*n* = 5). (**C**) ChIP-qPCR for SMAD3 binding to the promoters of *Aldh18a1*, *Pycr1*, and *Eprs* in protomyofibroblast-like fibroblasts after si*Bcat1* treatment (control: *n* = 3; SMAD3: *n* = 9). (**D**) Intracellular levels of valine, leucine, and isoleucine in protomyofibroblast-like fibroblasts after si*Bcat1* treatment (*n* = 10). (**E**) Protein levels of ALDH18A1, PYCR1, EPRS, BCAT1, p-SMAD3, SMAD3, and GAPDH in si*Bcat1*-treated protomyofibroblast-like fibroblasts after BCAA treatment (*n* = 3). An unpaired, 2-tailed *t* test was used for **A**, **B**, and **D**. One-way ANOVA with Tukey’s test was used for **C** and **E**. *P < 0.05, **P < 0.01, and ***P < 0.001. Data are shown as the mean ± SD.

TGF-β promotes mRNA and protein expression of *Aldh1*8a1, *Pycr1*, and *Eprs* (Figure 4, C and D). We therefore focused on SMAD3 phosphorylation, a key mediator of TGF-β signaling (40, 41). The treatment of cardiac protomyofibroblast–like fibroblasts with an siRNA against *Smad3* significantly decreased the mRNA expression of *Aldh18a1*, *Pycr1*, *Eprs*, and *Col1a1* (Figure 6A), indicating that SMAD3 mediated the mRNA expression of these genes. We then knocked down *Bcat1* in protomyofibroblast-like fibroblasts, which revealed that the *Bcat1* knockdown significantly decreased the phosphorylation levels of SMAD3, thereby indicating that BCAT1 promoted the phosphorylation of SMAD3 in protomyofibroblast-like fibroblasts (Figure 6B). In fact, *Col1a1* mRNA levels in protomyofibroblast-like fibroblasts were significantly reduced by si*Bcat1* treatment (Supplemental Figure 5B), indicating that BCAT1 also contributed to the production of *Col1a1* mRNA.

Consistent with these findings, analysis of published ChIP-seq data (GSE145250) showed SMAD3 binding sites in the upstream regions of *ALDH18A1*, *PYCR1*, *EPRS*, and *COL1A1* (Supplemental Figure 5C). To validate this result, we performed ChIP-qPCR with an anti-SMAD3 antibody in protomyofibroblast-like fibroblasts and found significant SMAD3 enrichment at the promoter regions of *Aldh18a1*, *Pycr1*, and *Eprs* (Figure 6C). Notably, *Bcat1* knockdown markedly reduced SMAD3 occupancy at these sites (Figure 6C). Together, these results indicate that BCAT1 enhanced SMAD3 activation and promoted the transcription of *Aldh18a1*, *Pycr1*, and *Eprs* in protomyofibroblast-like fibroblasts.

We next examined whether BCAT1-mediated BCAA production enhances SMAD3-dependent transcription of these genes in protomyofibroblast-like fibroblasts. To determine whether BCAT1 primarily drives the anabolic or catabolic arm of BCAA metabolism in these cells, we measured intracellular BCAA levels after *Bcat1* knockdown. Both 3D-HPLC and GC-MS analyses showed that *Bcat1* knockdown significantly reduced intracellular levels of BCAAs, including valine, leucine, and isoleucine, in protomyofibroblast-like fibroblasts (Figure 6D, Supplemental Figure 4C, and Supplemental Data 1 and 2). Notably, BCAA supplementation restored ALDH18A1, PYCR1, and EPRS expression, as well as SMAD3 phosphorylation, after *Bcat1* knockdown (Figure 6E).

We finally asked whether BCAT1 expression alone is sufficient to induce SMAD3 phosphorylation and collagen production in the absence of mechanical stress or TGF-β signaling. We induced overexpression of BCAT1 in cardiac resident fibroblasts from normal mouse hearts and cultured them in suspension on nonadherent plates for 3 days to minimize mechanical cues and TGF-β–dependent activation. Under these conditions, SMAD3 phosphorylation and COL1A1 expression were largely unchanged in BCAT1-overexpressing fibroblasts (Supplemental Figure 5D). These results indicate that BCAT1 overexpression alone was insufficient to activate SMAD3 or induce collagen production.

### BCAT1 enhances phosphorylation of HDAC5 at Ser488, leading to subsequent SMAD3 phosphorylation.

**Figure 7:**
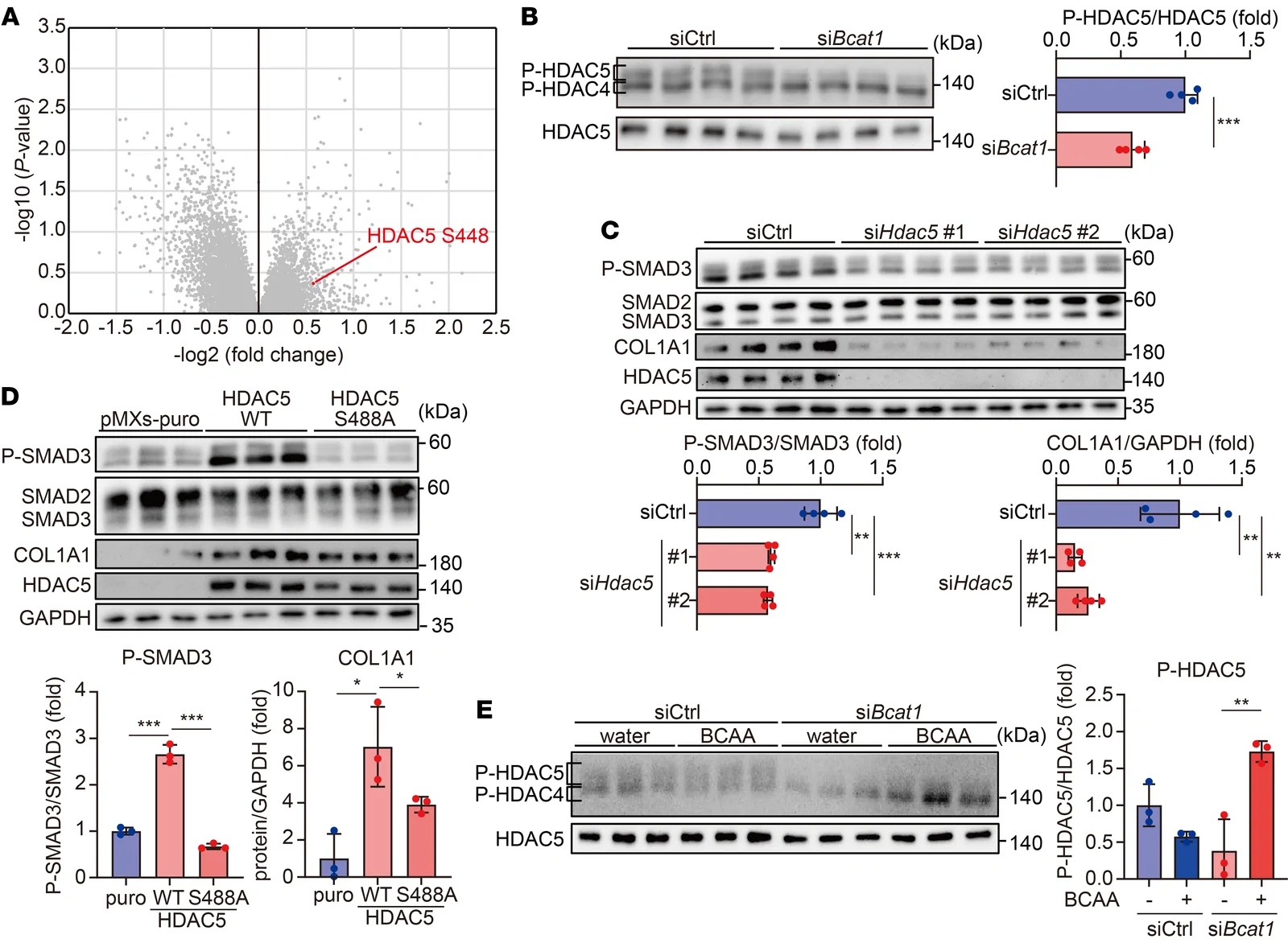
BCAT1 increases the phosphorylation of HDAC5 at Ser488 in protomyofibroblast-like fibroblasts through BCAA production. (**A**) Volcano plot of the differentially regulated phosphorylated peptides in which the protein expression was not reduced by si*Bcat1* (siCtrl/si*Bcat1* ratio >0.9) in protomyofibroblast-like fibroblasts after si*Bcat1* treatment (*n* = 4). (**B**) Protein levels of p-HDAC5 (Ser488) and HDAC5 in protomyofibroblast-like fibroblasts after si*Bcat1* treatment (*n* = 4). (**C**) Protein levels of p-SMAD3, SMAD3, COL1A1, HDAC5, and GAPDH in protomyofibroblast-like fibroblasts after si*Hdac5* treatment (*n* = 4). (**D**) Protein levels of p-SMAD3, SMAD3, COL1A1, HDAC5, and GAPDH in protomyofibroblast-like fibroblasts after pMXs-puro, HDAC5 WT, or HDAC5 S488A overexpression (*n* = 3). (**E**) Protein levels of p-HDAC5, and HDAC5 in protomyofibroblast-like fibroblasts with si*Bcat1* treatment after BCAA treatment (*n* = 3). An unpaired, 2-tailed *t* test was used for **A**–**C**. One-way ANOVA with Tukey’s test was used for **D** and **E**. *P < 0.05, **P < 0.01, and ***P < 0.001. Data are shown as the mean ± SD.

Next, we performed phosphoproteomics analysis of *Bcat1*-knockdown protomyofibroblast-like fibroblasts to identify molecules linking BCAT1 to SMAD3 phosphorylation (Supplemental Data 4). Among the altered phosphoproteins, phosphorylation of HDAC5 at Ser488, which has been reported to promote SMAD3 phosphorylation, was markedly reduced by *Bcat1* knockdown (Figure 7A). We validated this finding by Western blotting using an antibody against phosphorylated mouse HDAC4 (Ser629)/HDAC5 (Ser488) and confirmed the phosphorylated HDAC5 (p-HDAC5) band by *Hdac5* siRNA treatment (Supplemental Figure 6). Consistently, we found that *Bcat1* knockdown reduced HDAC5 (Ser488) phosphorylation in protomyofibroblast-like fibroblasts (Figure 7B). Moreover, *Hdac5* knockdown suppressed both SMAD3 phosphorylation and COL1A1 protein expression, as previously reported (42) (Figure 7C). To assess the functional relevance of HDAC5 (Ser488) phosphorylation, we induced overexpression of either WT HDAC5 or a phospho-null mutant (S488A). WT HDAC5 increased SMAD3 phosphorylation, whereas the S488A mutant did not (Figure 7D). COL1A1 protein expression was also markedly increased by WT HDAC5, but not comparably by the S488A mutant (Figure 7D). Importantly, BCAA supplementation restored HDAC5 (Ser488) phosphorylation after *Bcat1* knockdown (Figure 7E). Together, these results indicate that BCAT1-mediated BCAA production promoted HDAC5 (Ser488) phosphorylation, leading to SMAD3 activation and induction of *Aldh18a1*, *Pycr1*, and *Eprs* expression in protomyofibroblast-like fibroblasts.

### Increased cardiac expression of ALDH18A1, PYCR1, and EPRS and post-MI cardiac fibrosis are attenuated in Bcat1-KO mice.

**Figure 8:**
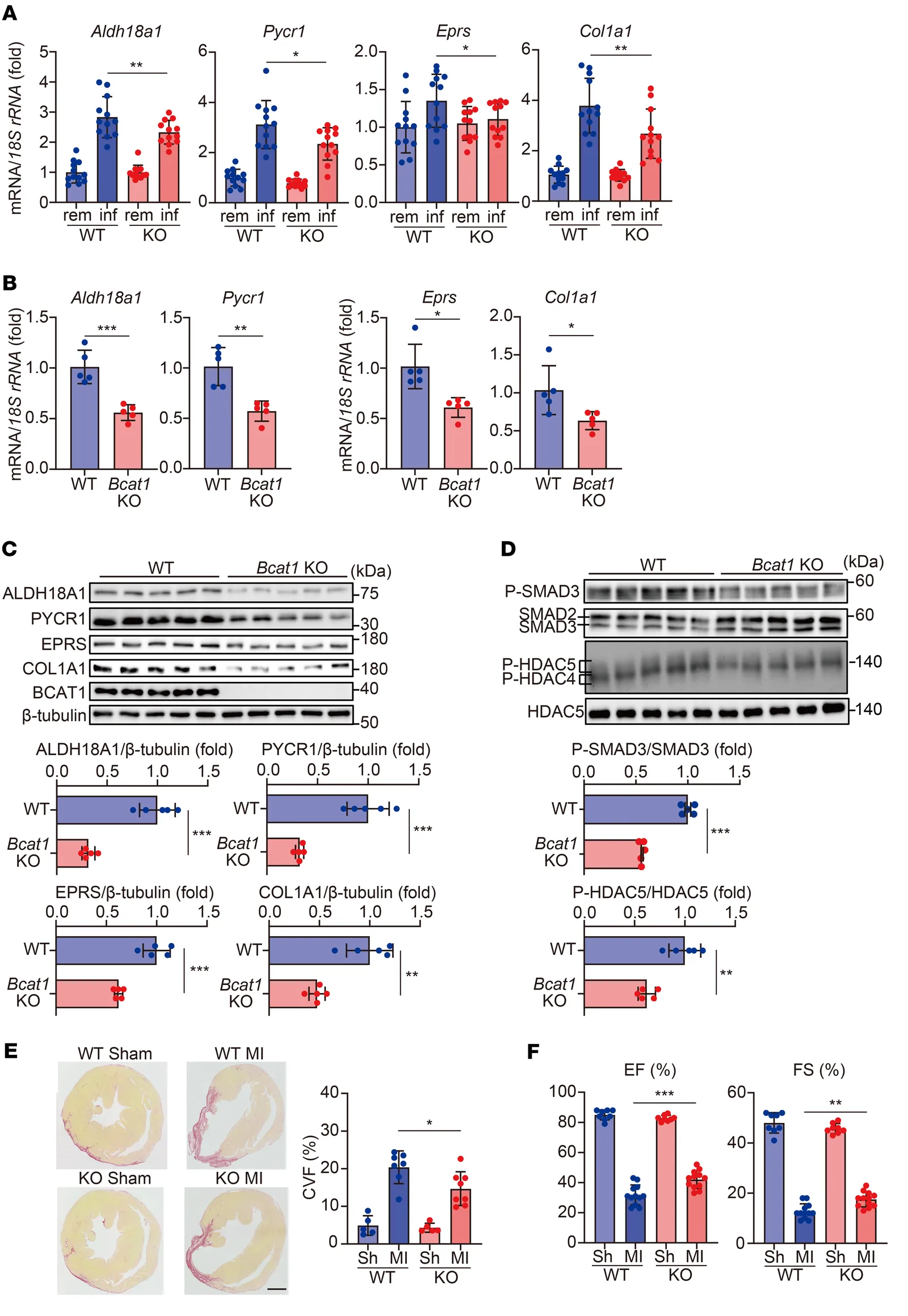
*Bcat1* deficiency attenuates mouse cardiac fibrosis and improves cardiac function after MI. (**A**) mRNA levels of *Aldh18a1*, *Pycr1*, *Eprs*, and *Col1a1* in infarcted (inf) or noninfarcted (rem) areas of WT or *Bcat1*-KO (KO) mouse hearts 3 days after MI (*n* = 12). (**B**) mRNA levels of *Aldh18a1*, *Pycr1*, *Eprs*, and *Col1a1* in protomyofibroblast-like fibroblasts isolated from WT or *Bcat1*-KO mouse hearts 3 days after MI (*n* = 5). (**C** and **D**) Protein levels of COL1A1, ALDH18A1, PYCR1, EPRS, BCAT1, and β-tubulin (**C**), p-SMAD3, SMAD3, p-HDAC5, and HDAC5 (**D**), in protomyofibroblast-like fibroblasts isolated from WT or *Bcat1*-KO mouse hearts 3 days after MI (*n* = 4–5). (**E**) Collagen staining of WT or *Bcat1*-KO mouse hearts 28 days after sham treatment (Sh) or MI. The collagen volume fraction (CVF) was calculated as the percentage of the Picrosirius red^+^ area (WT sham/MI: *n* = 5/7, KO sham/MI: *n* = 5/8). Scale bar: 1 mm. (**F**) Cardiac function in WT or *Bcat1*-KO mice 28 days after sham treatment or MI (WT sham/MI: *n* = 8/12, KO sham/MI: *n* = 8/12). An unpaired, 2-tailed *t* test was used for **B**–**D**. One-way ANOVA with Tukey’s test was used for **A**, **E**, and **F**. *P < 0.05, **P < 0.01, and ***P < 0.001. Data are shown as the mean ± SD.

To assess BCAT1 function in vivo, we established *Bcat1*-KO mice (Supplemental Figure 7, A–C) (43). We examined mRNA expression of *Aldh18a1*, *Pycr1*, *Eprs*, and *Col1a1* in remote nonfibrotic areas (rem) and infarcted fibrotic areas (inf) of WT and *Bcat1*-KO mouse hearts 3 days after MI. qRT-PCR revealed that induction of *Aldh18a1*, *Pycr1*, *Eprs*, and *Col1a1* mRNA expression in fibrotic areas was significantly attenuated in *Bcat1*-KO mice as compared with that in WT mice (Figure 8A). Consistent with this result, we found that mRNA expression of *Aldh18a1*, *Pycr1*, *Eprs*, and *Col1a1* was decreased in isolated cardiac protomyofibroblast–like fibroblasts of *Bcat1*-KO mouse hearts as compared with that in WT mouse hearts (Figure 8B). We further observed that protein expression levels of ALDH18A1, PYCR1, and EPRS were reduced in the fibrotic area and isolated cardiac protomyofibroblast–like fibroblasts of *Bcat1*-KO mouse hearts after MI, as compared with those of WT mouse hearts (Figure 8C and Supplemental Figure 7D). In addition, the protein expression levels of COL1A1 and phosphorylated SMAD3 and HDAC5 were decreased in the fibrotic area and isolated protomyofibroblast-like fibroblasts of *Bcat1*-KO mouse hearts 3 days after MI (Figure 8D and Supplemental Figure 7E). The decrease in cardiac fibrosis after MI in *Bcat1*-KO mice was verified by Picrosirius red staining of heart sections 28 days after MI (Figure 8E). Thus, we examined cardiac function in WT and *Bcat1*-KO mice 28 days after MI using echocardiography. The results showed that ejection fraction (EF) and fractional shortening (FS) rates were significantly increased in *Bcat1*-KO mice as compared with those in WT mice (Figure 8F, Supplemental Figure 7F, and Supplemental Table 1). These results indicated that BCAT1 promoted the protein expression of enzymes related to proline synthesis and translation in protomyofibroblast-like fibroblasts, leading to collagen accumulation in the heart after MI. In contrast, mRNA expression of proinflammatory genes in the infarcted area and in these cells was comparable between WT and *Bcat1*-KO mice 3 days after MI (Supplemental Figure 7, G and H). Moreover, infiltration of CD45^+^ macrophages into the fibrotic area and mRNA expression levels of *Tnf*, *Tgfb1*, *Il1b*, and *Cxcl2* in macrophages isolated from WT and *Bcat1*-KO hearts after MI did not significantly differ between the 2 groups (Supplemental Figure 7, I and J). We also observed no difference in the proliferative capacity of fibrogenic fibroblast–lineage cells between WT and *Bcat1*-KO mice at post-MI day 4 (Supplemental Figure 7K). Taken together, these findings indicate that BCAT1 did not contribute to the inflammation that initiated fibrosis or to the proliferation of fibrogenic fibroblast–lineage cells in fibrotic hearts.

### Inhibition of BCAT1 attenuates cardiac fibrosis and dysfunction after MI.

**Figure 9:**
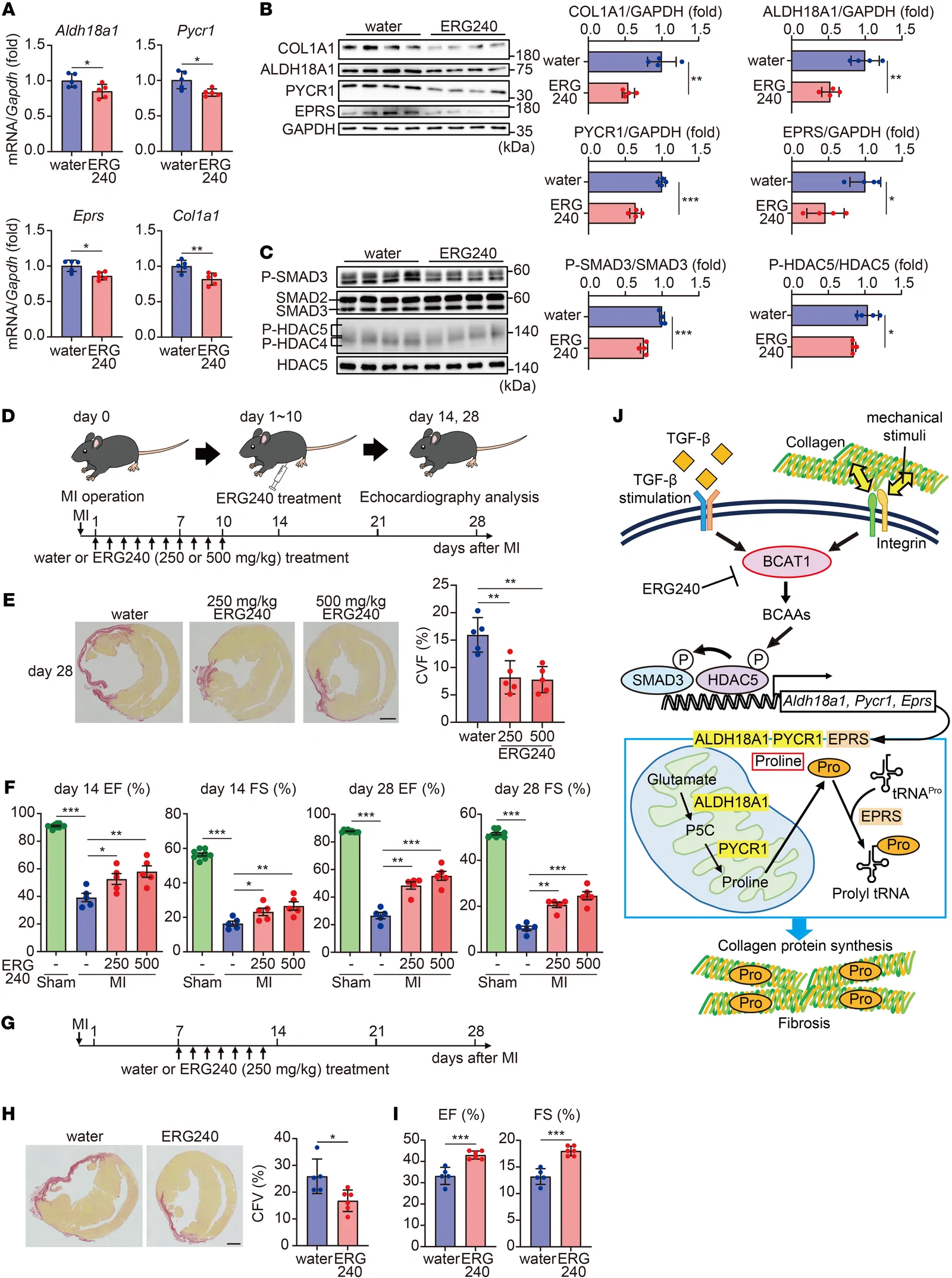
ERG240 treatment attenuates mouse cardiac fibrosis and improves cardiac function after MI. (**A**) mRNA levels of *Aldh18a1*, *Pycr1*, *Eprs*, and *Col1a1* in protomyofibroblast-like fibroblasts after ERG240 treatment (*n* = 5). (**B** and **C**) Protein levels of COL1A1, ALDH18A1, PYCR1, EPRS, and GAPDH (**B**), and of p-SMAD3, SMAD3, p-HDAC5, and HDAC5 (**C**) in protomyofibroblast-like fibroblasts after ERG240 treatment (*n* = 4). (**D**) Schematic image of water or ERG240 treatment for 10 days after MI. (**E**) Collagen staining of the heart 28 days after MI in mice administrated ERG240 for 10 days. The CVF was calculated as the percentage of the Picrosirius red^+^ area (*n* = 5). Scale bar: 1 mm. (**F**) Cardiac function 14 and 28 days after sham treatment or MI in mice administrated ERG240 for 10 days (sham/MI: *n* = 9/5). (**G**) Schematic of treatment for 7 days with water or ERG240 (250 mg/kg) starting at post-MI day 7. (**H**) Collagen staining of the heart 28 days after MI in mice administrated ERG240 for 7 days, starting at post-MI day 7. The CVF was calculated as the percentage of the Picrosirius red^+^ area (water: *n* = 5, ERG240: *n* = 6). Scale bar: 1 mm. (**I**) Cardiac function 28 days after sham treatment or MI in mice administrated ERG240 for 7 days, starting at post-MI day 7 (water: *n* = 5, ERG240: *n* = 6). (**J**) Schematic of the BCAT1-mediated signaling pathway that enhances collagen expression in protomyofibroblast-like fibroblasts after MI. An unpaired, 2-tailed *t* test was used for **A**–**C**, **E**, **H**, and **I**. One-way ANOVA with Tukey’s test was used for **F**. *P < 0.05, **P < 0.01, and ***P < 0.001. Data are shown as the mean ± SD.

Finally, we investigated the effects of ERG240 (29), a specific inhibitor of BCAT1, on cardiac fibrosis and dysfunction after MI. We first examined whether treatment of protomyofibroblast-like fibroblasts with ERG240 affects the expression levels of *Aldh18a1*, *Pycr1*, and *Eprs*. We observed that ERG240 treatment significantly suppressed mRNA and protein expression of these enzymes in this cell population (Figure 9, A and B).

We further found that the protein expression levels of COL1A1 and the phosphorylation levels of SMAD3 and HDAC5 were significantly suppressed by ERG240 treatment in protomyofibroblast-like fibroblasts of fibrotic hearts 3 days after MI (Figure 9, B and C). In contrast, ERG240 treatment did not alter the infiltration of CD45^+^ macrophages into the fibrotic area or the mRNA expression levels of *Tnf*, *Tgfb1*, *Il1b*, and *Cxcl2* in macrophages isolated from fibrotic hearts (Supplemental Figure 8, A and B), indicating that ERG240 did not enhance inflammatory responses.

We next administered ERG240 to WT mice for 10 days, starting the day after MI, and measured collagen accumulation in the hearts 28 days after MI and cardiac function at 14 and 28 days after MI (Figure 9D). We found that ERG240 administration significantly reduced collagen accumulation in mouse hearts after MI (Figure 9E). The administration also attenuated decreased in the EF and FS, indicating reduced post-MI cardiac dysfunction (Figure 9F, Supplemental Figure 8C, and Supplemental Table 2).

We next examined whether ERG240 administration initiated on day 7 after MI, when acute inflammation shifts to the chronic inflammatory phase, could ameliorate fibrosis (Figure 9G). We found that ERG240 treatment initiated on day 7 after MI and continued for 7 consecutive days significantly reduced cardiac collagen deposition 28 days after MI (Figure 9H). In addition, treatment with ERG240 attenuated the decline in EF and FS (Figure 9I, Supplemental Figure 8D, and Supplemental Table 3). These findings demonstrate that BCAT1 inhibition mitigated post-MI cardiac fibrosis and functional deterioration during both the acute and chronic inflammatory phases.

## Discussion

In this study, we demonstrated that *Bcat1* expression was induced by both TGF-β stimulation and mechanical stimulation in fibrogenic fibroblast–lineage cells. BCAT1 promoted the expression of ALDH18A1, PYCR1, and EPRS, all of which are proline biosynthesis–related molecules (Figure 9J). In mouse hearts, *Bcat1* expression peaked by day 3 and remained high even by day 28 after MI (Figure 2A). During the acute inflammatory phase (0–3 days after MI), many immune cells, such as macrophages, had infiltrated the infarcted area. At this stage, *Bcat1* expression in protomyofibroblast-like fibroblasts was likely induced primarily by immune cell–derived TGF-β in the infarcted area. In contrast, 28 days after MI, the amount of TGF-β was greatly decreased as compared with that at 3 days (Figure 2A) in the infarcted hearts, but the tissue stiffness had increased greatly due to fibrosis progression. Therefore, we believe that mechanical stimulation predominantly induced *Bcat1* expression in myofibroblasts 28 days after MI. Importantly, in the post-MI hearts, expression of *Bcat1* peaked on day 3 after MI treatment, whereas expression of various collagens peaked on post-treatment day 7 (Figure 2A). These results indicated that *Bcat1* was rapidly induced by TGF-β in protomyofibroblast-like fibroblasts at an early phase of myofibroblast differentiation to produce proline, the main amino acid present in collagen, to allow smooth translation of collagen protein. Consistent with *Bcat1* expression, *Aldh18a1*, *Pycr1*, and *Eprs* also showed peak expression levels on day 3 after MI (Figure 4E), suggesting coordinated activation of the proline biosynthetic program in protomyofibroblast-like fibroblasts during the early phase after MI. Although the codetection analysis of *Bcat1* with *Postn* suggests that BCAT1 contributes to collagen production in protomyofibroblast-like fibroblasts, future studies using an inducible *Postn*-Cre–based approach (13) for *Bcat1* deletion would be needed to directly determine the functional contribution of BCAT1 in protomyofibroblast-lineage cells.

BCAT1 is expressed in human monocyte–derived macrophages, and administration of a BCAT1 inhibitor suppresses macrophage infiltration and collagen accumulation in arthritis and glomerulonephritis (29). However, in the setting of MI, scRNA-seq (Figure 2C), qRT-PCR (Supplemental Figure 2D), and ISH (Supplemental Figure 2E) consistently showed that *Bcat1* was not expressed in macrophages. Consistent with this, ERG240 administration did not alter macrophage infiltration. Moreover, a previous study demonstrated that the proinflammatory effects of BCAT1 in macrophages depend on IRG1 expression (29). In contrast, *Irg1* was barely detectable in resident fibroblasts and fibrogenic fibroblast–lineage cells in our scRNA-seq analysis (Supplemental Figure 2C) and bulk RNA-seq analysis (GSE244876). These findings suggest that the BCAT1/IRG1 inflammatory axis operates predominantly in macrophages in arthritis and glomerulonephritis but is unlikely to contribute to BCAT1-mediated effects in cardiac fibrogenic fibroblast–lineage cells after MI. Thus, the decrease in collagen accumulation in the infarcted hearts of *Bcat1*-KO mice (Figure 8E) and ERG240-treated WT mice (Figure 9, E and H) was probably not due to reduced inflammation in the infarcted area.

We identified BCAT1 as an enzyme that promotes collagen production by enhancing both proline synthesis and the generation of prolyl-tRNA in fibrogenic fibroblast–lineage cells. This enhancement of proline synthesis by BCAT1 is also found in blast crisis chronic myeloid leukemia with high BCAT1 expression, although the previous report measuring intracellular amino acid levels in chronic myeloid leukemia cells only focused on activated BCAAs (valine [Val], leucine [Leu], isoleucine [Ile]) production by BCAT1, and did not mention the relationship between proline levels and BCAT1 (28). Our study demonstrated that BCAAs generated by BCAT1 increased intracellular proline levels by upregulating the expression of proline biosynthetic enzymes, including ALDH18A1, PYCR1, and EPRS, through HDAC5 and SMAD3 phosphorylation.

Notably, BCAT1 overexpression alone in cardiac resident fibroblasts did not induce SMAD3 phosphorylation or COL1A1 protein expression (Supplemental Figure 5D). These findings suggest that BCAT1 requires additional profibrotic cues, such as TGF-β signaling and/or mechanical stimulation, to exert its full fibrogenic effects, warranting further mechanistic studies.

We focused on BCAT1-mediated collagen protein production through enhanced proline synthesis and increased expression of translation-related enzymes. We also found that BCAT1 promoted the transcription of *Col1a1* under both in vitro and in vivo conditions (Figure 8, A and B, and Supplemental Figure 5C). Together, these findings suggest that the effect of BCAT1 on COL1A1 protein production involved both transcriptional and translational pathways. Importantly, proline supplementation significantly increased COL1A1 protein levels (Figure 5H), underscoring the importance of BCAT1-mediated proline synthesis in driving efficient production of proline-rich collagen.

Moreover, the mechanism by which BCAAs generated by BCAT1 induce HDAC5 phosphorylation at Ser488 remains unclear. Previous studies have shown that HDAC5 (Ser488) can be directly phosphorylated by protein kinase D (44) and AMP-activated protein kinase (45), suggesting that these kinases may act downstream of BCAT1-mediated BCAA production in protomyofibroblast-like fibroblasts. Alternatively, BCAT1-derived BCAAs may influence HDAC5 phosphorylation indirectly through broader changes in intracellular signaling. Further investigation will be required to clarify how BCAA metabolism is linked to HDAC5 (Ser488) phosphorylation and to identify the kinase or signaling pathway that mediates this effect.

Accumulating evidence indicates that metabolic reprogramming is associated with myofibroblast differentiation (5). For example, mitochondrial synthesis and glycolysis have been reported to be activated during their differentiation (5). It has also been reported that the expression levels of enzymes that metabolize amino acids, such as glycine, glutamic acid, and proline, are altered during myofibroblast differentiation. Similar to proline-metabolizing enzymes, previous reports have demonstrated that synthesis of proline by ALDH18A1 and PYCR1 and generation of prolyl-tRNA by EPRS promote the production of collagen protein in fibrogenic fibroblast–lineage cells (21–24). However, the detailed mechanisms regulating their expression in this cell population remain unclear. Here, we showed that BCAT1 expression was specifically induced in a substantial subset of *Postn*-expressing protomyofibroblast-like fibroblasts and controlled the mRNA expression of *Aldh18a1*, *Pycr1*, and *Eprs* through HDAC5 and SMAD3 phosphorylation. We also found that *Bcat1* knockdown only modestly suppressed the expression of these mRNAs. However, BCAT1 markedly affected collagen protein production. We believe that this is because BCAT1 not only promotes the generation of proline through ALDH18A1 and PYCR1, which is required for collagen production*,* but also acts on subsequent collagen synthesis through EPRS. Identification of the role of BCAT1, which controls the proline metabolism that underlies collagen protein production, has broad implications for the development of therapeutic strategies to combat fibrotic heart disease.

## Methods

### Sex as a biological variable.

We examined male mice because female mice were reported to be resistant to the acute MI model (46). Whether our findings apply to female mice requires further study.

### Surgical procedure.

C57BL/6N mice were purchased from Japan SLC and maintained in cages (5 mice per cage) under a 12-hour light/12-hour dark cycle.

For the MI-induced cardiac fibrosis mouse model, 8- to 10-week-old male mice were anesthetized and subjected to ligation of the anterior branch of the left coronary artery. Sham-operated mice underwent the same procedure, except for ligation.

For BCAT1 inhibitor experiments, 8- to 10-week-old male mice were treated i.p. with water or ERG240 (250 or 500 mg/kg), and cardiac collagen accumulation and function were assessed on day 28.

For the mouse model of liver fibrosis associated with MASH, 8-week-old male mice were fed a CDAHFD consisting of 60 kcal% fat and 0.1% methionine (A06071302N, Research Diets) for 10 weeks. Control mice received a standard diet (A06071314N, Research Diets).

For the mouse model of liver fibrosis induced by carbon tetrachloride (CCl_4_), CCl_4_ was administered i.p. to 8-week-old male mice twice a week for 4–6 weeks.

### Generation of Bcat1-KO mice.

*Bcat1*-KO mice were generated using the i-GONAD method (43) and Matsuyama’s protocol at Shigei Medical Research Institute. Integrated DNA Technologies (Coralville) synthesized the CRISPR gRNA and single-stranded oligodeoxynucleotides (ssODNs). The sgRNA (a mix of CRISPR RNA [crRNA] and trans-activating CRISPR RNA [tracrRNA]) targeted exon 2 of *Bcat1* (NC_000072.7), with the following sequence: 5′-GGATGCTCCGCGCCGTTTGCTGG-3′. Electroporation was performed using an electroporator (NEPA21, Nepagene).

### qRT-PCR analysis.

Using RLT Plus buffer, containing 1% 2-mercaptoethanol (QIAGEN), we lyzed cardiac protomyofibroblast–like fibroblasts and myofibroblasts, cardiac resident fibroblasts, activated HSCs, IMR-90, CCD-18Co, and LX-2 cells. We collected cell lysates using QIAshredder (QIAGEN). Sham-operated mouse hearts and normal or MASH mouse livers were rapidly frozen in liquid nitrogen and stored at –80°C. MI-operated mouse hearts were divided into infarcted and noninfarcted areas and were stored at –80°C. Total RNA was extracted from mouse tissues stored at –80°C using ISOGEN (Nippon Gene). This was followed by purification using the RNeasy Mini Kit (QIAGEN). After RNA purification, cDNA was synthesized using the High-Capacity cDNA Reverse Transcription Kit (Life Technologies, Thermo Fisher Scientific). mRNA expression was quantified using the Luna Universal qPCR Master Mix (New England BioLabs) protocol. qRT-PCR probes were purchased from MilliporeSigma (Supplemental Table 4). mRNA expression levels were calculated by the ΔΔCt method using GAPDH or 18S ribosomal RNA as an internal control.

### Isolation of protomyofibroblast-like fibroblasts and myofibroblasts from post-MI fibrotic hearts and resident fibroblasts from normal hearts.

Mouse hearts collected 3 days after MI and normal mouse hearts were digested in PBS containing 0.1% collagenase type II (Worthington Biochemical) and 0.01% elastase (Worthington Biochemical) at 37°C for 20 minutes. After digestion, debris and RBCs were removed using Debris Removal Solution (Miltenyi Biotec) and RBC lysis buffer (Roche), respectively. For infarcted hearts, cells were plated overnight in DMEM (Nacalai Tesque) containing 10% FBS (Gibco, Thermo Fisher Scientific) and 1% penicillin-streptomycin (Nacalai Tesque), and adherent cells were harvested with Accutase (Nacalai Tesque). Cells were then incubated with CD45 antibody–conjugated magnetic beads (1:10, Miltenyi Biotec) in magnetically activated cell-sorting buffer (0.5% BSA from MilliporeSigma and 2 mM EDTA in PBS) and subjected to MS column separation. The CD45^–^ fraction was defined as cardiac protomyofibroblast–like fibroblasts and myofibroblasts, as described previously (30), whereas resident fibroblasts from normal hearts were further enriched according to PDGFRα or THY1.2 positivity.

### Regulation of myofibroblast differentiation by mechanical stimulation.

As previously reported (30), cardiac myofibroblasts from mouse hearts 3 days after MI were cultured on plastic plates, and total RNA and protein were extracted from a portion of the cultured cardiac myofibroblasts. The remaining adherent myofibroblasts were detached from the plates using 0.25% trypsin/1 mM EDTA solution and cultured in ultra-low-attachment surface cell culture plates (Sumitomo Bakelite). After culturing, some of the suspended cells were collected by centrifugation, and total RNA and protein were extracted. The remaining suspended cells were cultured on plastic plates, and total RNA and protein were extracted from these re-adhered cardiac myofibroblasts.

### Cell culture.

NIH3T3 and CCD-18Co cell lines were purchased from the American Type Culture Collection (ATCC). LX-2 cell lines were purchased from Merck Millipore (Burlington). Plat-E cells were provided by T. Kitamura (Tokyo University). IMR-90 cell lines were purchased from RIKEN BRC. NIH3T3 cells, Plat-E cells, cardiac resident fibroblasts, and activated HSCs were cultured in DMEM supplemented with 10% FBS and 1% penicillin-streptomycin. Protomyofibroblast-like fibroblasts and myofibroblasts isolated from post-MI fibrotic hearts were cultured in DMEM with 10% FBS and 1% penicillin-streptomycin or glutamate, or in glutamine-deficient DMEM (GMEP) with 10% FBS and 1% penicillin-streptomycin. CCD-18Co cells were cultured in MEM (Nacalai Tesque) supplemented with 10% FBS, 1 mM sodium pyruvate solution (Nacalai Tesque), and 1% penicillin-streptomycin. LX-2 cells were cultured in DMEM supplemented with 2% FBS and 1% penicillin-streptomycin. IMR-90 cells were cultured in MEM supplemented with 10% FBS and 1% penicillin-streptomycin.

### Knockdown using siRNA.

Protomyofibroblast-like fibroblasts and myofibroblasts, activated HSCs, CCD-18Co cells, and LX-2 cells were transfected with siRNAs using Lipofectamine RNAiMAX (Invitrogen, Thermo Fisher Scientific). Total RNA or protein was collected 72 or 96 hours after transfection, respectively. siRNAs were purchased from Life Technologies (Thermo Fisher Scientific) (Supplemental Table 5).

### Retrovirus transduction.

Plat-E cells were transfected with BCAT1-HA/pMXs-puro, HA-HDAC5/pMXs-puro, or HA-HDAC5-S488A/pMXs-puro using PEI-Max (Polysciences). Forty-eight hours after transfection, supernatants were collected and used to infect protomyofibroblast-like fibroblasts in the presence of polybrene (10 μg/mL, MilliporeSigma). Total protein lysates were harvested 72 hours after transduction.

### Preparation of polyacrylamide hydrogels with varying stiffness.

Polyacrylamide hydrogels were prepared as described previously (47). Briefly, 32 mm micro cover glasses (Matsunami) were treated with 1% 3-aminopropyltrimethoxysilane (MilliporeSigma) in methanol. Silanized coverslips were coated with different acrylamide/bis-acrylamide compositions as described before (47). After polymerization, the hydrogels were crosslinked with Pierce Sulfo-SANPAH, No-Weigh Format (Thermo Fisher Scientific) and sterilized by UV light. The gels were rinsed with 0.5 M HEPES (pH 7.5) and reacted to 0.1 mg/mL fibronectin (MilliporeSigma).

### Stimulation of cells with various reagents.

Cardiac protomyofibroblast–like fibroblasts and myofibroblasts were stimulated with 2 μM latrunculin A (Abcam), 30 μM BTT-3033 (Tocris Bioscience), or 2 mM ERG240 for 4, 8, or 72 hours, respectively. Cardiac resident fibroblasts from normal mouse hearts were stimulated with 5 ng/mL recombinant TGF-β (240-B; R&D Systems), 10 ng/mL recombinant TNF-α (315-01A-20UG; Peprotech), or 100 ng/mL LPS (L4391, MilliporeSigma) for 48 hours after starvation in DMEM/0.1% FBS/1% penicillin-streptomycin for 24 hours.

### Western blotting.

Cardiac protomyofibroblast–like fibroblasts and myofibroblasts, cardiac resident fibroblasts from normal mouse hearts, activated HSCs, CCD-18Co cells, LX-2 cells, and infarcted areas of WT or *Bcat1*-KO mouse hearts from 3 days after MI were lyzed in RIPA buffer (50 mM TrisHCl [pH 8.0]/150 mM NaCl/0.5% sodium deoxycholate/0.1% SDS/1% NP40/protease inhibitors [1:100; Nacalai Tesque]/phosphatase inhibitors [1:100, Nacalai Tesque]) at 4°C. The solution was then centrifuged, and the supernatants were collected. A portion of the supernatant was mixed with 4′ SDS sample buffer (200 mM Tris-Cl [pH 6.8]/40% glycerol/4% SDS/0.08% bromophenol blue) containing 1% 2-mercaptoethanol and boiled at 95°C.

For immunoblotting to detect COL1A1 protein, the cells and tissues were lysed in RIPA buffer with 1% 2-mercaptoethanol, incubated at 4°C, boiled at 95°C before centrifugation, and the supernatants were then mixed with 4× SDS sample buffer.

The samples were electrophoretically separated using SDS-polyacrylamide gel electrophoresis and were transferred to polyvinylidene fluoride membranes. The membranes were blocked with 5% skim milk/TBS-T and incubated with the primary antibodies, followed by incubation with HRP-conjugated secondary antibodies. Western blot images were acquired using AN enhanced chemiluminescence system (PerkinElmer) and analyzed using Image Lab software (Bio-Rad). All antibodies used in this study are listed in Supplemental Table 6.

### Determination of l-proline and BCAAs using 3D-HPLC.

Protomyofibroblast-like fibroblasts treated with an siRNA were collected from culture dishes with Accutase (Nacalai Tesque). After centrifugation, the supernatant was removed, and the collected cells were washed with PBS. Cells were mixed with water and MeOH, and the solution was centrifuged to obtain the supernatant. A portion of the supernatant was dried under reduced pressure, and the residue was redissolved with water. Amino acids were derivatized with 4-fluoro-7-nitro-2,1,3-benzoxadiazole (NBD-F) to form NBD-Pro, -Val, -Leu, and -Ile as described previously (48), and the reaction mixture was injected into the 3D-HPLC system. NBD-Pro, -Val, -Leu, and -Ile was isolated using microboreODS column (Singularity RP18) in the first dimension and using a mixed-mode column (Singularity MX-103) in the second dimension. Then, the isolated fraction of NBD-Pro, -Val, -Leu, and -Ile was introduced into an enantioselective column (Singularity CSP-001S) in the third dimension to separate d- and l-forms of NBD-Pro, -Val, -Leu, and -Ile. Detection of NBD-Pro, -Val, -Leu, and -Ile enantiomers was performed according to their fluorescence (excitation, 470 nm; emission, 530 nm).

### ChIP-qPCR.

Protomyofibroblast-like fibroblasts treated with an siRNA were crosslinked with 1% formaldehyde and then quenched with 0.25 M glycine. Cells were collected and subjected to ChIP using the SimpleChIP Enzymatic Chromatin IP Kit (9003; Cell Signaling Technology) with an anti-SMAD2/-3 antibody. The eluted DNA was analyzed with the StepOnePlus Real-Time PCR system (Applied Biosystems) using the primers listed in Supplemental Table 7.

### TMT-based proteomics analysis.

Protomyofibroblast-like fibroblasts were treated with an siRNA, washed with ice-cold HEPES-saline (20 mM HEPES-NaOH [pH 7.5] and 137 mM NaCl), detached by Accutase, and lysed in guanidine-TCEP buffer (8 M guanidine-HCl, 100 mM HEPES-NaOH [pH 7.5], 10 mM TCEP, 40 mM CCA). After heating and sonication, 200 μg protein was purified by methanol-chloroform precipitation and resuspended in 0.1% RapiGest SF (Waters) in 50 mM triethylammonium bicarbonate. Proteins were further sonicated, heated, and digested with trypsin/Lys-C mix (Promega) for 16 hours at 37°C. Peptide concentrations were determined using the Pierce quantitative colorimetric peptide assay (Thermo Fisher Scientific). Approximately 130 μg peptides was labeled with TMTpro 16-plex reagent (Thermo Fisher Scientific) for 1 hour at 25°C. After quenching with hydroxylamine, labeled samples were pooled, acidified with trifluoroacetic acid (TFA), and fractionated by offline high-pH reversed-phase chromatography on the Vanquish DUO UHPLC system (Thermo Fisher Scientific). The 48 fractions were consolidated into 16 fractions, evaporated in a SpeedVac concentrator, and dissolved in 3% acetonitrile (ACN) and 0.1% TFA.

Liquid chromatography–tandem MS (LC-MS/MS) analysis of the resulting peptides (500 ng each) was performed on an EASY-nLC 1200 UHPLC coupled to Q Exactive Plus mass spectrometer through a nanoelectrospray ion source (Thermo Fisher Scientific). Peptides were separated on a 75 μm inner diameter × 150 mm C18 reversed-phase column (Nikkyo Technos) using a linear gradient of 4%–20% ACN for 0–115 minutes and 20%–32% ACN for 115–160 minutes, followed by an increase to 80% ACN for 10 minutes and a final 10-minute hold at 80% ACN. The mass spectrometer was operated in data-dependent acquisition mode with a top-10 MS/MS method. MS1 spectra were acquired at a resolution of 70,000, an automatic gain control (AGC) target of 3 × 10^6^, and a mass range of 375–1,400 *m/z*. HCD MS/MS spectra were acquired at a resolution of 35,000 with an AGC target of 1 × 10^5^, an isolation window of 0.7 *m/z*, a maximum injection time of 150 ms, and a normalized collision energy of 33. Dynamic exclusion was set to 20 seconds. Raw data were analyzed against the *Mus musculus* SwissProt database using Proteome Discoverer, version 2.5 (Thermo Fisher Scientific), with the Sequest HT search engine for protein identification and TMT quantification. Search parameters included trypsin with up to 2 missed cleavages, a precursor mass tolerance of 10 ppm, and a fragment mass tolerance of 0.02 Da. TMTpro labeling of lysine residues and peptide N-termini and carbamidomethylation of cysteine were set as fixed modifications, and methionine oxidation was set as a variable modification. Peptides and proteins were filtered at a FDR of 1% using the percolator node and the protein FDR validator node, respectively. Normalization was performed so that the total abundance across all peptides was equal for each TMT channel.

### RNA-seq analysis.

RNA-seq analysis was performed as previously described (30). The RNA-seq dataset after *Bcat1* knockdown in protomyofibroblast-like fibroblasts isolated from post-MI fibrotic hearts was deposited in the Gene Expression Omnibus (GEO) database (GEO GSE244876). The previously deposited microarray datasets were GSE158236 (mechanical stimulation microarray) and GSE158157 (MI-operated heart microarray). RNA-seq datasets for patients were obtained from the GEO database (HF: GSE161472; and MASH: GSE126848). The expression levels of the target genes were determined after normalizing raw counts.

### scRNA-seq analysis.

scRNA-seq data were analyzed using the R package Seurat, version 3.2.2, as previously described (49). The scRNA-seq dataset was obtained from ArrayExpress (E-MTAB-7376).

### ISH.

Mouse hearts and livers were fixed overnight in 4% paraformaldehyde (Nacalai Tesque)/0.1 M phosphate buffer, embedded in Tissue-Tek O.C.T. compound (Sakura), and rapidly frozen in liquid nitrogen. The tissue sections were prepared at 6 μm thickness using the Cryostar NX70 (Thermo Fisher Scientific).

To detect *Bcat1*, *Col1a1*, or both mRNAs in mouse hearts and livers, tissue sections were subjected to the RNAScope Multiplex Fluorescent v2 Assay using RNAScope probes in accordance with the manufacturer’s instructions. After mRNA was labeled with tyramide-Cy3 or tyramide-Cy5 (PerkinElmer), tissue sections were blocked with 10% BSA and PBS and stained with each primary antibody, followed by staining with the appropriate secondary antibodies. After immunostaining, nuclei were stained with DAPI. Tissue staining was evaluated under a confocal microscope (LSM700 or LSM980, Carl Zeiss). All antibodies used in this study are listed in Supplemental Table 6.

### Picrosirius red staining.

WT or *Bcat1*-KO mouse hearts were collected 28 days after sham treatment or MI. The collected mouse hearts were fixed in 10% neutral buffered formalin solution (Wako) and embedded in paraffin. Cardiac paraffin sections (4 μm thick) were deparaffinized and incubated in Picrosirius red buffer (3% picric acid/0.15% Direct Red). The sections were incubated in 0.01 N HCl and dehydrated. The stained sections were observed under a microscope (BZ-X700; Keyence) and analyzed using the Keyence BZ-II analyzer.

### GO enrichment analysis.

In the scRNA-seq analysis, genes showing increased expression in resident fibroblasts and fibrogenic fibroblast–lineage cells compared with *Bcat1*-negative fibroblasts [−log_10_(*P* value) > 30, log_2_(fold change) > 0] were analyzed using DAVID 2021.

### Echocardiography.

Echocardiographic analysis was performed with 2D-targeted M-mode tracings obtained using Nemio XG image analysis system (SSA-580A, Toshiba Medical Systems). Mice after sham treatment or MI were anesthetized with 2% isoflurane gas throughout the echocardiographic procedure, while their body temperature was maintained at 37°C with a heating pad and their heart rate was maintained at approximately 500 bpm. FS was calculated as follows: FS = (left ventricular internal diameter in diastole [LVIDd] – left ventricular internal diameter in systole [LVIDs])/LVIDd) × 100. The EF was calculated as follows: EF = (end-diastolic volume [EDV] – end-systolic volume [ESV])/EDV) × 100. EDV and ESV were calculated using the Pombo’s method (50).

### Synthesis of ERG240.

ERG240 synthesis, nuclear magnetic resonance analysis, and the structure, purity, and molecular formula strings are described in the Supplemental Methods.

### Statistics.

Statistical analyses were performed using GraphPad Prism version 9.3.1 (GraphPad Software). All experiments were replicated at least 3 times. All data were shown as the mean ± SD. Statistical significance was determined using a 2-tailed, unpaired Student’s *t* test for comparisons between 2 groups and 1-way ANOVA with Tukey’s test for multiple comparisons. *P* values of less than 0.05 were considered statistically significant.

### Study approval.

All animal procedures were approved by the IACUC of Kyushu University (A23-092-0) and Nagoya University (R250027-005). All experiments were performed in accordance with relevant national and international guidelines, including Japan’s Act on the Welfare and Management of Animals.

### Data availability.

Values underlying all figures are provided in the Supporting Data Values file. RNA-seq data generated in this study are deposited in the Gene Expression Omnibus (GEO) database (GSE244876). Public RNA-seq, ChIP-seq, and scRNA-seq datasets were obtained from the GEO database (GSE161472, GSE126848, and GSE145250) and ArrayExpress (E-MTAB-7376). The R scripts used are available from the authors upon request. The MS proteomics data have been deposited to the ProteomeXchange Consortium via the jPOST partner repository with the dataset identifiers PXD075667 and PXD075668.

## Author contributions

NT, TH, and MN conceptualized and designed the research. NT, TH, HS, KY, HW, and YH performed in vivo and in vitro experiments. NT and YH performed animal surgeries. HM and GH synthesized the compounds. YN and KH measured intracellular amino acid levels. HK performed proteomics analysis. NT, HW, and MN wrote the original draft of the manuscript.

## Conflict of interest

The authors have declared that no conflict of interest exists.

## Funding support

- Grants-in-Aid for Scientific Research (KAKENHI) (JP26K02168, JP25K22523, JP23K27323 to MN).
- The Takeda Science Foundation (to MN).
- The Mochida Memorial Foundation for Medical and Pharmaceutical Research (to MN).
- Princess Takamatsu Cancer Research Fund (to MN).
- Ono Medical Research Foundation (to MN).
- Hoansha Foundation (to MN).
- The Yasuda Medical Foundation (to MN).
- KOSÉ Cosmetology Research Foundation (to MN).
- Japan Agency for Medical Research and Development (AMED) (JP23am0401003s0305, JP25gm6710018h0003, JP25fk0210127h0003, and JP26gm6710018h0004, to MN).
- Grant-in-Aid for JSPS Research Fellow (19J20083, to YH; 21J11273, to TH; 22J20790, to NT).
- Make New Standards Program for the Next Generation Researchers (I260029 to HW).

## Supplementary Material

Supplemental data

Supplemental data sets 1-4

Unedited blot and gel images

Supporting data values

## Version 1 07/31/2026

In-Press Preview

## Version 2 09/01/2026

Electronic publication
