# Supplementary material for: Branched-chain amino acid transaminase 1–mediated pathway promotes proline-dependent collagen production in cardiac myofibroblasts
Source: J Clin Invest. 2026 Jul 16;136(17):e182216. doi: 10.1172/JCI182216 (PMC13528941; doi:10.1172/JCI182216)
Supplement: Supplementary file 1 [file jci-136-182216-s285.pdf]

Supplemental Methods

Metabolic analysis using GC-MS

Intracellular amino acid levels were measured in siRNA-treated proto-myofibroblast-like fibroblasts. Cells were detached with accutase (Nacalai Tesque), extracted with 75%–80% methanol, and centrifuged, and the supernatants were purified using MonoSpin C18 columns. (GL Sciences). Samples were dried, derivatized by methoximation with methoxyamine in pyridine followed by trimethylsilylation, and analyzed using a GC–MS system (TQ-8050NX, Shimadzu). Metabolites were identified based on mass-to-charge ratios and retention times.

Isolation of activated HSCs from fibrotic liver using magnetic-activated cell sorting

Fibrotic mouse livers, generated by CCl4 treatment, were perfused with Hanks' balanced salt solution (HBSS; Nacalai Tesque) containing 25 mM HEPES and 0.5 mM EDTA and digested in HBSS containing 0.1% Collagenase A (Roche) and 1.5 mM HEPES at 37°C for 30 min. After the digestion, hepatocytes and red blood cells were removed by centrifugation and red blood cell lysis buffer (Roche), respectively. Activated HSCs were isolated from the remaining hepatic cells in the same manner as for cardiac proto-myofibroblast-like fibroblasts and myofibroblasts, and CD45-negative cells were defined as activated HSCs.

ChIP-seq analysis

The published ChIP-seq dataset was obtained from the GEO database (GSE145250). The data were analyzed by trimming and mapping and visualized using the Integrated Genome Viewer (https://software.broadinstitute.org/software/igv/).

Genotyping

We confirmed genome editing of *Bcat1* by genotyping (genotyping primer: *Bcat1* Fw 5′-TGTTGGACAGTACAGACTTGGC-3′; *Bcat1* Rv 5′-CCCTAATCTCAACCTGAAATGC-3′). We verified deficiency of BCAT1 protein in KO mice by western blot using anti-BCAT1 antibody (1:4,000, MA5-25892; Invitrogen, Waltham, MA, USA).

EdU assay

WT and *Bcat1*-KO mice were intraperitoneally injected with EdU (10 μg/g body weight) at3 days after MI. Hearts were harvested 24 h later, fixed in 4% paraformaldehyde in 0.1 M

phosphate buffer, embedded, and sectioned at 6 μm. EdU incorporation was detected using the Click-iT EdU Imaging Kit (C10337; Thermo Fisher Scientific) according to the manufacturer's instructions, prior to immunostaining.

Synthesis of ERG240

Experiments and Reagents

Reactions were carried out under an argon atmosphere unless noted otherwise. Reactions were monitored by thin-layer chromatography using Merck Silica Gel 60 F254 plates. Flash chromatography was performed using flash silica gel (CHROMATOREX PSQ 60B (spherical neutral, particle size 60 μm) purchased from Fuji Silysia Chemical Co. Ltd.

Instrumentation

NMR spectra were recorded 500 MHz Bruker Avance III, operating at 500 MHz for ^{1}H NMR and 126 MHz for ^{13}C NMR. Chemical shifts were reported in the scale relative to $CHCl_3$ (δ 7.26 ppm for ^{1}H NMR, δ 77.16 ppm for ^{13}C NMR) or H_2O (δ 4.79 ppm for ^{1}H NMR) 1,4-dioxane (δ 67.19 ppm in D_2O for ^{13}C NMR) as an internal reference. Splitting patterns are designated as s: singlet, d: doublet, t: triplet, q: quartet, br: broadening, and m: multiplet. High-resolution mass spectrometry (HRMS) was obtained with Bruker MicrOTOF II.

4-methyl-5-oxohexanoic acid [CAS: 6818-07-1]

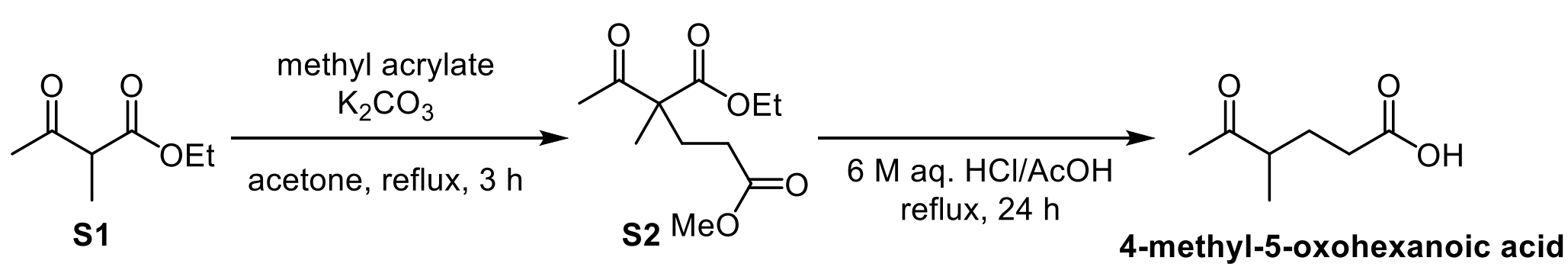

This compound was synthesized following a reported procedure [1].

To a suspension of ethyl 2-methylacetoacetate (**S1**) (5.0 mL, 35 mmol) and K_2CO_3 (24.3 g, 176 mmol) in acetone (140 mL), methyl acrylate (3.8 mL, 42 mmol) was added. The reaction mixture was stirred for 3 h under reflux, then the volatiles were removed under reduced pressure. The residue was treated with Et_2O (100 mL) and water (100 mL). After the separation of the layers, the aqueous phase was extracted with Et_2O (2 x 100 mL). The combined organic layer was dried over $MgSO_4$, filtered through a cotton plug, and then the filtrate was concentrated under reduced pressure to give the crude diester **S2** and it was used in the next step without further purification.

Crude diester **S2** was dissolved into 6 M aqueous HCl and AcOH (20/20 mL). The solution was stirred for 24 under reflux, then the volatiles were removed under reduced pressure. The residue was extracted with Et_2O (3 x 100 mL). The combined organic layer was dried over $MgSO_4$, filtered through a cotton plug, and then the filtrate was concentrated under reduced pressure. The residue was purified with flash column chromatography (SiO_2, 200 mL, Hexane/EtOAc = 9/1 to 2/1) to give 4-methyl-5-oxohexanoic acid (2.93 g, 58% over 2 steps) as a yellow oil. The spectral data of the obtained compound was well-matched to the reported one.

^{1}H NMR (500 MHz, $CDCl_3$): δ 2.60 (tq, *J* = 7.0, 7.0 Hz, 1H, H4), 2.42–2.29 (m, 2H, H2), 2.16 (s, 3H, H6), 2.05–1.96 (m, 2H, H3a), 1.72–1.62 (m, 2H, H3b), 1.13 (d, *J* = 7.0 Hz, 3H, H4').
^{13}C NMR (126 MHz, $CDCl_3$): δ 212.2, 179.2, 46.1, 31.6, 28.4, 27.2, 16.4.
HRMS (ESI): *m/z* calculated for $C_7H_{12}NaO_3$ $[M+Na]^+$ 167.0684, found: 167.0683.

ERG240 [CAS: 1415683-79-2]

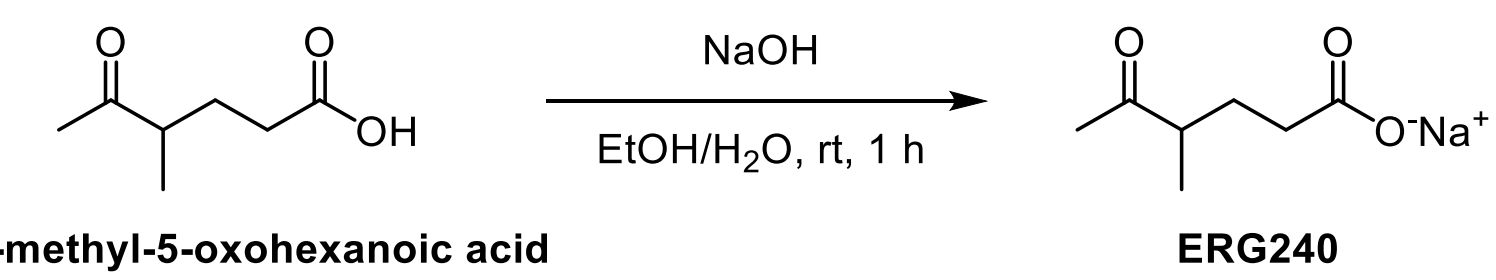

To a solution of ketoacid 4-methyl-5-oxohexanoic acid (2.93 g, 20.3 mmol) in EtOH (20 mL) was added 1 M aqueous NaOH (19.2 mL, 19.2 mmol). The reaction mixture was stirred for 1 h and then the volatiles were removed under reduced pressure. The resultant solid was washed with Et_2O (3 x 10 mL) to give ERG240 (2.72 g, 85%) as a white solid.

^{1}H NMR (500 MHz, D_2O): δ 2.68 (tq, *J* = 7.0, 7.0 Hz, 1H, H4), 2.25 (s, 3H, H6), 2.20–2.14 (m, 2H, H2), 1.95–1.86 (m, 2H, H3a), 1.69–1.60 (m, 2H, H3b), 1.10 (d, *J* = 7.0 Hz, 3H, H4').
^{13}C NMR (126 MHz, D_2O): δ 220.8, 183.2, 47.2, 35.6, 29.5, 28.4, 15.9.
HRMS (ESI): *m/z* calculated for $C_7H_{11}Na_2O_3$ $[M+Na]^+$ 189.0504, found: 189.0513.

Reference:

1. Holloway, C. A., Muratore, M. E., Storer, R. L. & Dixon, D. J. Direct enantioselective Brønsted acid catalyzed N-acyliminium cyclization cascades of tryptamines and ketoacids. *Org. Lett.* **12**, 4720–4723 (2010).

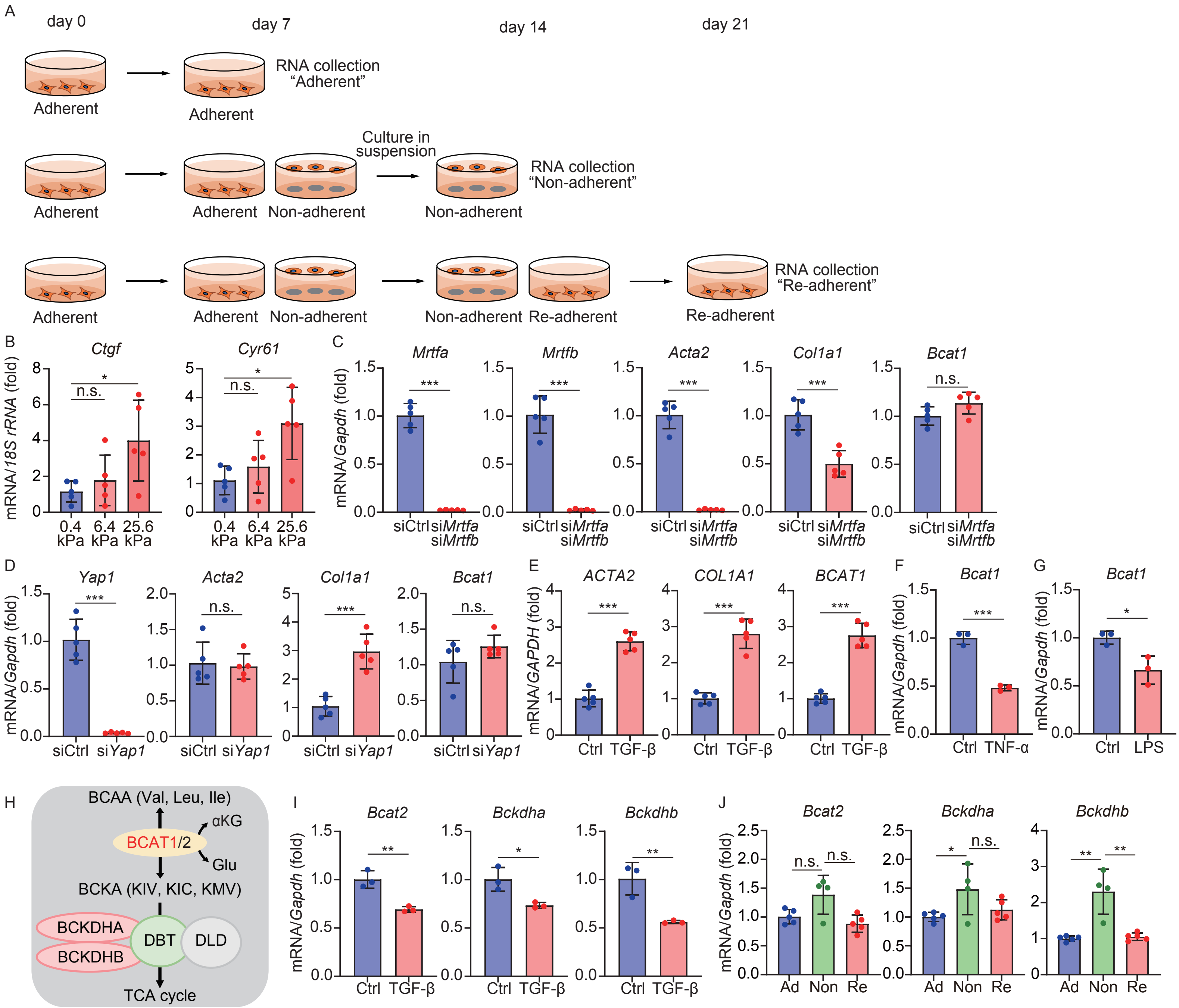

Supplemental Figure 1. *Bcat1*, expression but not *Bcat2, Bckdha* and *Bckdhb* expression is induced in myofibroblasts by mechanical stimulation and TGF-β signaling.
(**A**) Schematic image of the protocol for myofibroblast differentiation regulated by mechanical stimulation. (**B**) mRNA levels of *Ctgf*, and *Cyr61* in NIH3T3 cells cultured in 0.4kPa, 6.4kPa, or 25.6kPa stiffness gels for 12h ($n = 5$). (**C**) mRNA levels of *Mrtfa, Mrtfb, Acta2, Col1a1*, and *Bcat1* in cardiac myofibroblasts after si*Mrtfa* and si*Mrtfb* treatment ($n = 5$). (**D**) mRNA levels of *Yap1, Acta2, Col1a1*, and *Bcat1* in cardiac myofibroblasts after si*Yap1* treatment ($n = 5$). (**E**) mRNA levels of *ACTA2, COL1A1*, and *BCAT1* in IMR-90 cells after TGF-β stimulation ($n = 5$). (**F–G**) mRNA level of *Bcat1* in cardiac resident fibroblasts isolated from normal mouse hearts after TNF-α stimulation ($n = 3$) (**F**) or LPS stimulation ($n = 3$) (**G**). (**H**) Schematic diagram of BCAA catabolism. BCAA: Branched chain amino acids, Val: Valine, Leu: Leucine, Ile: Isoleucine, α-KG: α-ketogurtarate, Glu: Glutamate, BCKA: Branched chain keto acids, KIV: α-ketoisovalerate, KIC: α-ketoisocaproate, KMV: α-keto-β-methylvalerate, BCKDHA/B: BCKA dehydrogenase E1 subunit A/B, DBT: dihydrolipoamide branched chain transacylase E2, DLD: dihydrolipoamide dehydrogenase, TCA cycle: tricarboxylic acid cycle. (**I**) mRNA levels of *Bcat2, Bckdha*, and *Bckdhb* in cardiac resident fibroblasts isolated from normal mouse hearts after TGF-β stimulation ($n = 3$). (**J**) mRNA levels of *Bcat2, Bckdha*, and *Bckdhb* in adherent (Ad), non-adherent (Non), or re-adherent (Re) cardiac myofibroblasts isolated from mouse hearts 3 days after MI ($n = 4–5$). Unpaired *t* test was used for **B**–**G**, **I**, and **J**. n.s.: not significant, *$P < 0.05$, **$P < 0.01$ ***$P < 0.001$.

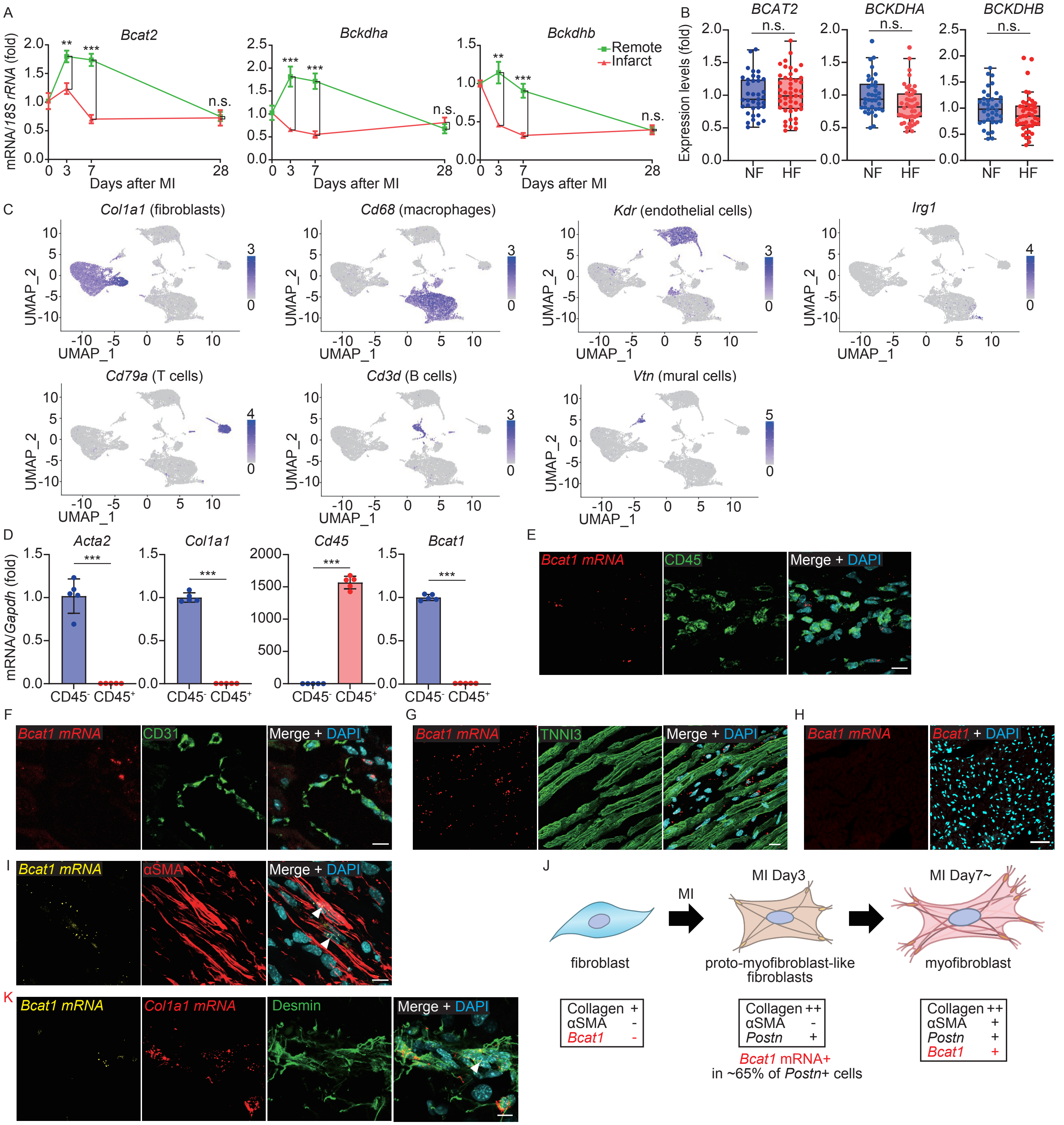

Supplemental Figure 2. *Bcat1* expression, but not *Bcat2, Bckdha,* and *Bckdhb* expression, is specifically induced in proto-myofibroblast-like fibroblasts and myofibroblasts of fibrotic hearts and livers.

(**A**) mRNA levels of *Bcat2*, *Bckdha*, and *Bckdhb* in intact mouse hearts or mouse hearts 3, 7, or 28 days after sham or MI. The MI-operated mouse hearts were divided into infarcted (infarct) and non-infarcted (remote) areas (n = 3–6). (**B**) Expression levels of *BCAT2*, *BCKDHA*, and *BCKDHB* in non-failing (NF) or failing (HF) human hearts (GSE161472, NF: n = 37, HF: n = 47). (**C**) UMAP plot of single-cell RNA-seq dataset (E-MTAB-7376) of stromal cells from mouse hearts 3 and 7 days after sham and MI. UMAP plot of *Col1a1, Cd68, Kdr, Cd79a, Cd3d, Vtn*, and *Irg1* expression in stromal cells of mouse hearts 3 and 7 days after sham and MI. The expressions of *Col1a1, Cd68, Kdr, Cd79a, Cd3d,* and *Vtn* were used as markers for each cell type. (**D**) mRNA levels of *Acta2, Col1a1, Cd45*, and *Bcat1* in CD45-negative cells and -positive cells isolated from mouse hearts 3 days after MI. (n = 5). (**E–G**) Co-detection of *Bcat1* mRNA, and CD45 (**E**), CD31 (**F**), and TNNI3 (**G**) in mouse hearts 3 days after MI. Scale bar: 10 µm. (**H**) *In situ* hybridization with *Bcat1* mRNA in mouse hearts 3 days after sham operation. Scale bar: 50 µm. (**I**) Co-detection of *Bcat1* mRNA and αSMA in mouse hearts 7 days after MI. Scale bar: 10 µm. (**J**) Stepwise differentiation of fibroblasts toward myofibroblasts. Quiescent fibroblasts (left) transition into proto-myofibroblast-like fibroblasts (middle; MI Day 3) and subsequently into myofibroblasts (right; MI Day 7). Proto-myofibroblast-like fibroblasts represent an intermediate state characterized by early activation prior to full myofibroblast differentiation. (**K**) Co-detection of *Bcat1* mRNA, *Col1a1* mRNA, and Desmin in the fibrotic livers of mice with MASH. Scale bar: 10 µm. Unpaired t test was used for **A**, **B** and **D**. Arrowheads indicate merged cells. n.s.: not significant. $^{**}P < 0.01$, $^{***}P < 0.001$.

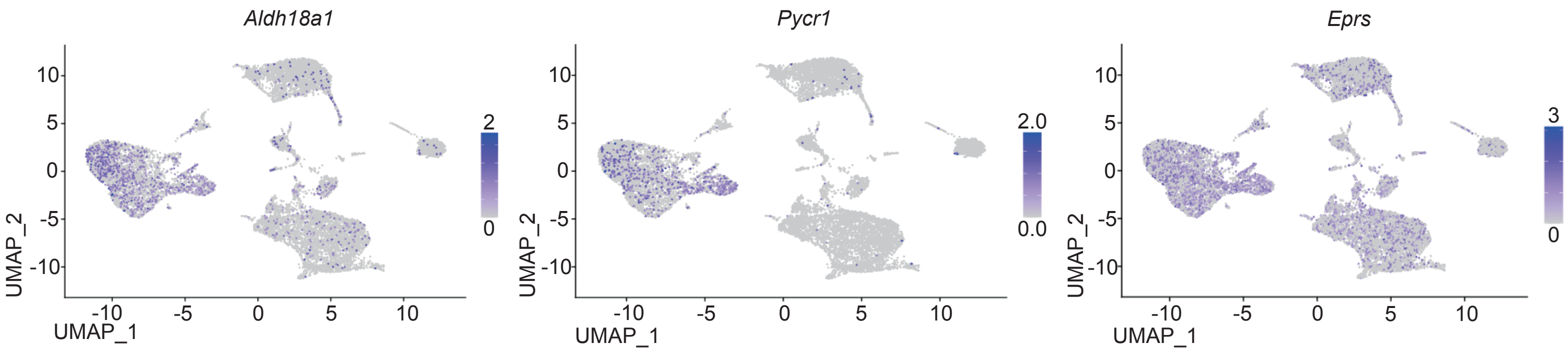

Supplemental Figure 3. *Aldh18a1*, *Pycr1*, and *Eprs* expression is detected in fibrotic hearts.
UMAP plot of *Aldh18a1*, *Pycr1*, and *Eprs* expression in the stromal cells from mouse hearts at 3 and 7 days after sham and MI.

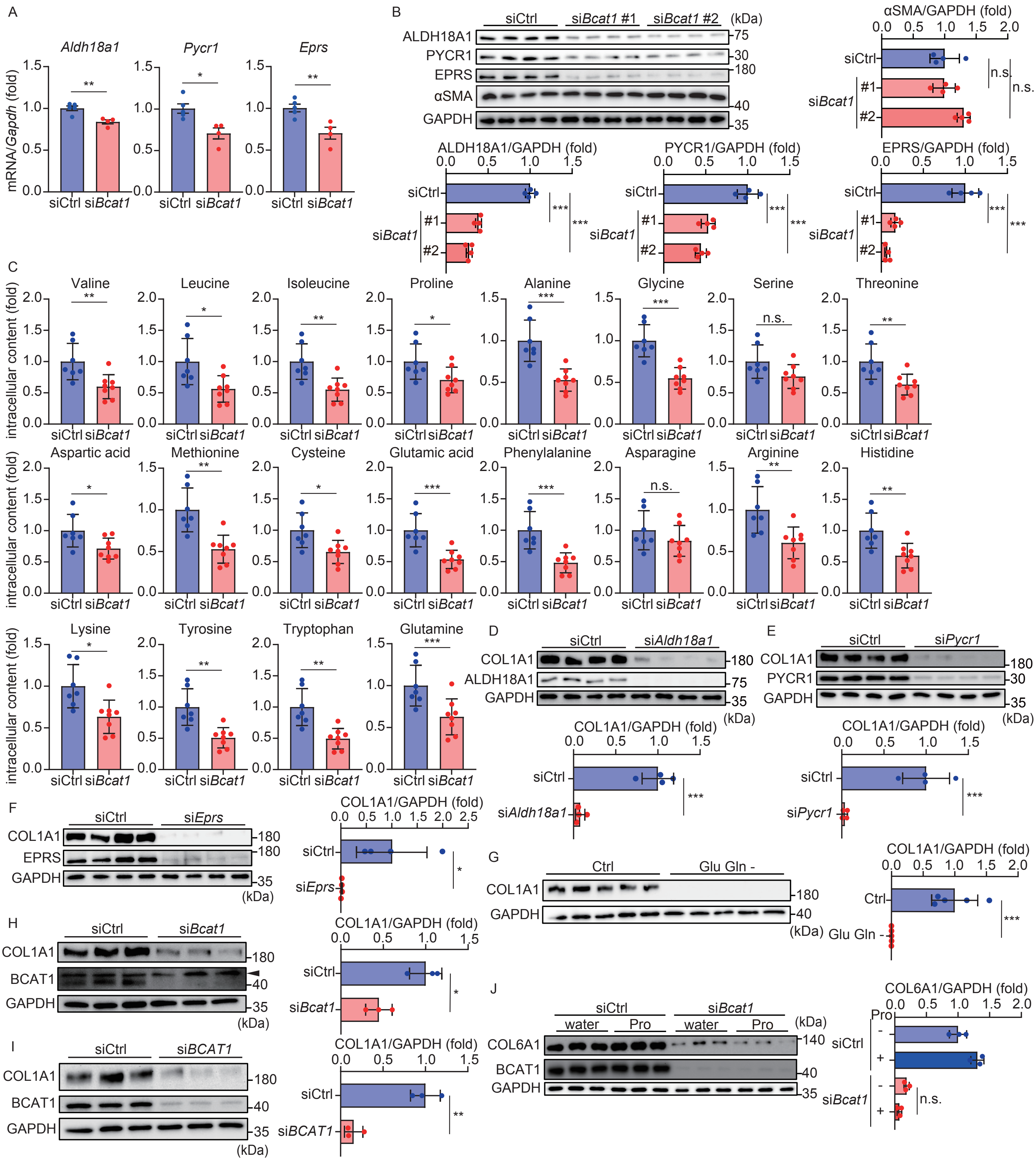

Supplemental Figure 4. BCAT1 promotes collagen expression via proline biosynthesis in proto-myofibroblast-like fibroblasts of fibrotic heart and activated HSCs of fibrotic liver.

(**A**) mRNA levels of *Aldh18a1, Pycr1*, and *Eprs* in activated HSCs isolated from CCl4 treated mouse livers after si*Bcat1* treatment (siCtrl: $n = 5$, si*Bcat1*: $n = 4$). (**B**) Protein levels of ALDH18A1, PYCR1, EPRS, αSMA and GAPDH in proto-myofibroblast-like fibroblasts of fibrotic hearts after si*Bcat1* treatment ($n = 4$). (**C**) Intracellular levels of amino acid in proto-myofibroblast-like fibroblasts after si*Bcat1* treatment (siCtrl: $n = 7$, si*Bcat1*: $n = 8$). (**D**) Protein levels of COL1A1, ALDH18A1, and GAPDH in proto-myofibroblast-like fibroblasts after si*Aldh18a1* treatment ($n = 4$). (**E**) Protein levels of COL1A1, PYCR1, and GAPDH in proto-myofibroblast-like fibroblasts after si*Pycr1* treatment ($n = 4$). (**F**) Protein levels of COL1A1, EPRS, and GAPDH in proto-myofibroblast-like fibroblasts after si*Eprs* treatment ($n = 4$). (**G**) Protein levels of COL1A1 and GAPDH in proto-myofibroblast-like fibroblasts after glutamate (Glu) and glutamine (Gln) deficiency ($n = 5$). (**H**) Protein levels of COL1A1, BCAT1, and GAPDH in liver activated HSCs after si*Bcat1* treatment ($n = 3$). A black arrowhead indicates non-specific signal. (**I**) Protein levels of COL1A1, BCAT1, and GAPDH in LX-2 cells after si*BCAT1* treatment ($n = 3$). (**J**) Protein levels of COL6A1, BCAT1, and GAPDH in proto-myofibroblast-like fibroblasts with si*Bcat1* treatment after proline treatment ($n = 3$). Unpaired *t* test was used for **A**–**I**. One-way ANOVA with Tukey's test was used for **J**. n.s.: not significant. $*P < 0.05$, $**P < 0.01$, $***P < 0.001$.

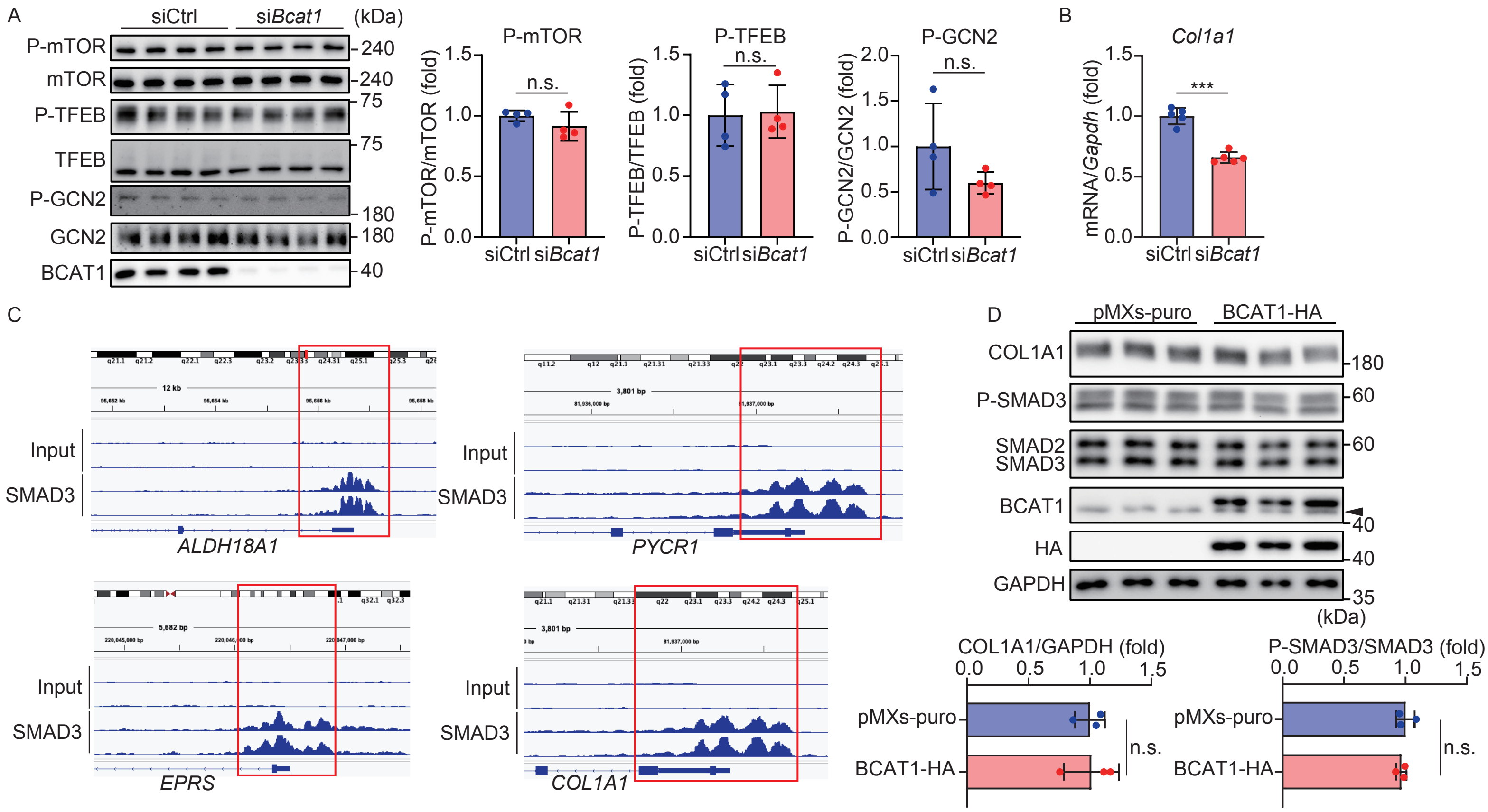

Supplemental Figure 5. BCAT1 promotes phosphorylation of SMAD3, which binds to promoter region of *ALDH18A1*, *PYCR1*, *EPRS*, and *COL1A1* in proto-myofibroblast-like fibroblasts.

(**A**) Protein levels of phosphorylated-mTOR, mTOR, phosphorylated-TFEB, TFEB, phosphorylated-GCN2, GCN2, and BCAT1 in proto-myofibroblast-like fibroblasts after si*Bcat1* treatment ($n = 4$). (**B**) mRNA levels of *Col1a1* in proto-myofibroblast-like fibroblasts after si*Bcat1* treatment ($n = 5$). (**C**) Gene track of *ALDH18A1, PYCR1*, *EPRS,* and *COL1A1* using ChIP-seq dataset (GSE145250) of H69 cells immunoprecipitated with anti-SMAD3 antibody after TGF-β stimulation ($n = 2$). (**D**) Protein levels of COL1A1, phosphorylated-SMAD3, SMAD3, BCAT1, HA, and GAPDH in cardiac resident fibroblasts isolated from normal mouse hearts after BCAT1-HA overexpression cultured in non-adherent plates for 3 days ($n = 4$). A black arrowhead indicates non-specific signal. Unpaired t test was used for **A**, **B**, and **D**. n.s.: not significant, $^{***}P < 0.001$.

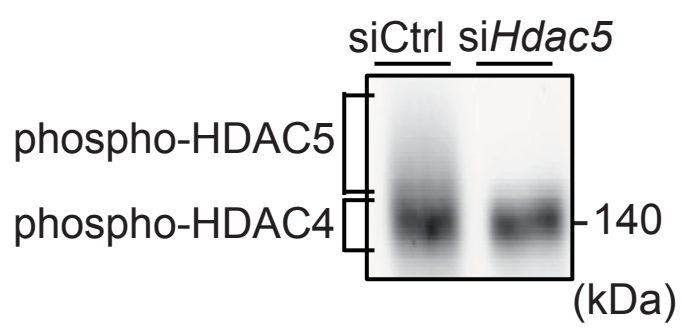

Supplemental Figure 6. Phosphorylated HDAC5 in proto-myofibroblast-like fibroblasts was decreased by treatment of siRNA against mouse *Hdac5*.

Protein levels of phosphorylated-HDAC5, and phosphorylated-HDAC4 in proto-myofibroblast-like fibroblasts were assessed by anti-phosphorylated-mouse HDAC4(Ser629)/HDAC5(Ser488) antibody.

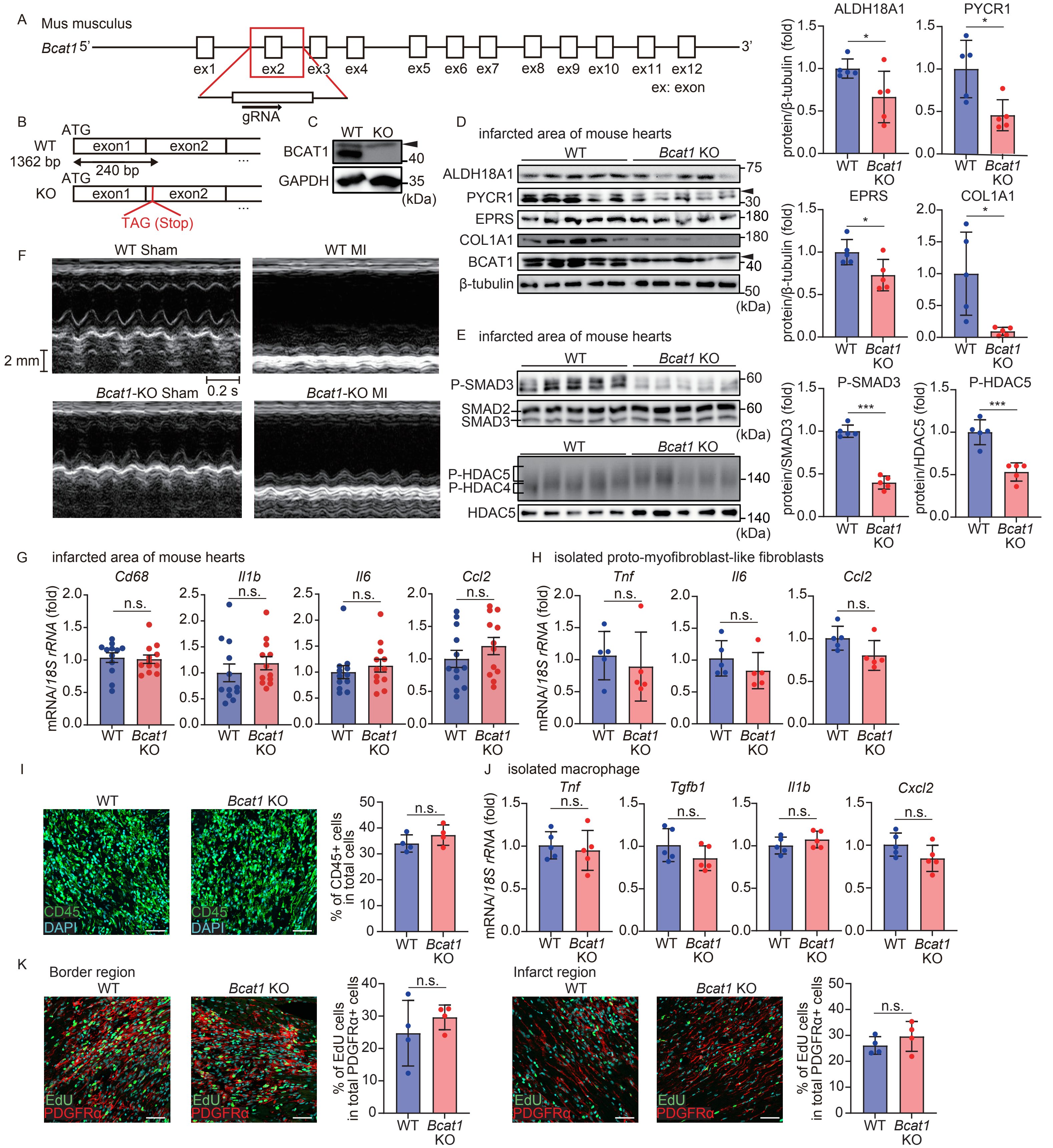

Supplemental Figure 7. Generation of *Bcat1*-KO mice and cardiac condition of *Bcat1*-KO mice after myocardial infarction. (**A**) Schematic image of the target site of a gRNA on the genomic sequence of *Bcat1*. (**B**) A stop codon (TAG) was newly generated in the *Bcat1* mRNA in *Bcat1*-KO mice. To prevent the expression of a variant starting from exon2, a stop codon was inserted immediately following the start codon of exon2. (**C**) Protein levels of BCAT1 and GAPDH in WT or *Bcat1*-KO mouse hearts 3 days after MI. (**D–E**) Protein levels of COL1A1, ALDH18A1, PYCR1, EPRS, BCAT1, and β-tubulin (**D**), phosphorylated SMAD3, SMAD3, phosphorylated HDAC5, and HDAC5 (**E**) in infarcted areas of WT or *Bcat1*-KO mouse hearts 3 days after MI ($n = 5$). Black arrowheads indicate non-specific signals. (**F**) Representative M-mode images of WT and *Bcat1*-KO mice 28 days after sham or MI. (**G**) mRNA levels of *Cd68, Il1b, Il6,* and *Ccl2* in infarcted areas of WT or *Bcat1*-KO mouse hearts 3 days after MI ($n = 12$). (**H**) mRNA levels of *Tnf, Il6*, and *Ccl2* in proto-myofibroblast-like fibroblasts isolated from WT or *Bcat1*-KO mouse hearts 3 days after MI ($n = 5$). (**I**) Co-detection of CD45 and DAPI in WT and *Bcat1*-KO mouse hearts 3 days after MI. The graph shows the percentages of CD45-positive cells in total cells of infarcted area ($n = 4$). Scale bar: 50 µm for x20. (**J**) mRNA levels of *Tnf, Tgfb1, Il1b*, and *Cxcl2* in macrophages isolated from WT or *Bcat1*-KO mouse hearts 3 days after MI ($n = 5$). (**K**) Immunohistochemistry of WT or *Bcat1*-KO mouse hearts 4 days after MI. The graph shows the percentages of EdU-positive cells in total PDGFRα-positive fibroblasts of border or infarcted area ($n = 4$). Scale bar: 50 µm for x20. Unpaired *t* test was used for **D, E, G–K**. n.s.: not significant, $*P < 0.05$, $***P < 0.001$.

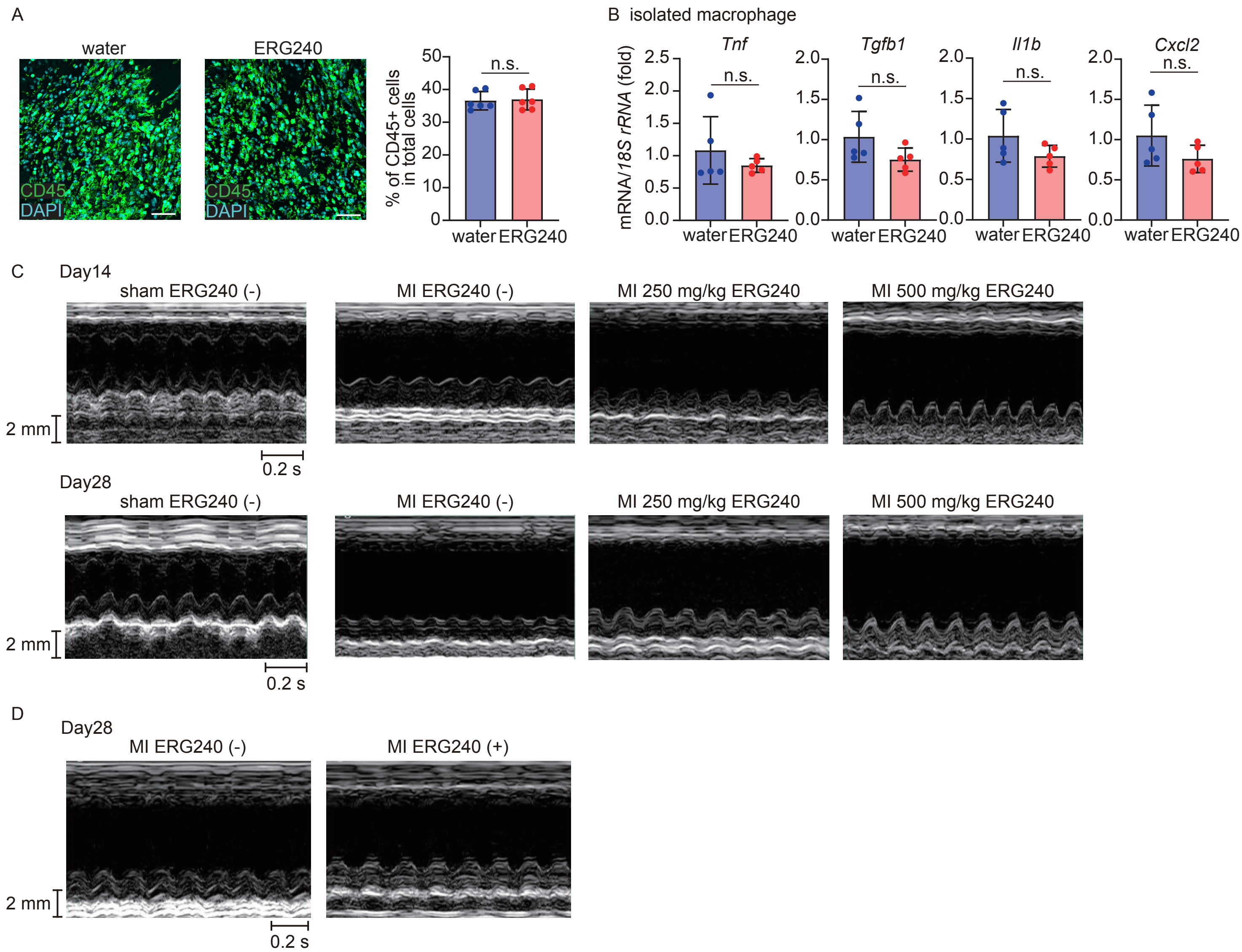

Supplemental Figure 8. Cardiac functions of mice after myocardial infarction with administration of ERG240.

(**A**) Co-detection of CD45 and DAPI in the hearts at 4 days after MI in mice administrated with ERG240 for 3 days. The graph shows the percentages of CD45-positive cells in total cells of infarcted area ($n = 6$). Scale bar: 50 μm for x20. (**B**) mRNA levels of *Tnf, Tgfb1, Il1b*, and *Cxcl2* in macrophages isolated from hearts 4 days after MI in mice administrated with ERG240 for 3 days. ($n = 5$). (**C**) Representative M-mode images of the hearts 14 or 28 days after sham or MI in mice administrated with ERG240 for 10 days. (**D**) Representative M-mode images of the hearts 28 days after MI in mice administrated with ERG240 starting at day 7 for 7 days. Unpaired *t* test was used for **A** and **B**. n.s.: not significant.

Supplemental Table 1. Echocardiographic measurements and heart weights of WT and *Bcat1* KO mice hearts on 28 days after sham or MI operation.

HW; heart weight, BW; body weight. IVSTd; interventricular septal thickness diastolic, LVIDd; left ventricular inner diameter diastolic, LVPWd; left ventricular posterior wall diameter diastolic, LVIDs; left ventricular inner diameter systolic, %EF; percent ejection fraction, %FS; percent fractional shortening. One-way ANOVA with Tukey's test was used for statistical analyses.

$^{\$}$ WT-MI vs WT-sham, $^{\#}$ KO-MI vs WT-MI

parameters	WT-sham (*n* =8)	WT-MI (*n* =12)	*Bcat1* KO-sham (*n* =8)	*Bcat1* KO-MI (*n* =12)
HW/BW (mg/g)	3.29±1.25	8.44±2.13$^{\$}$ (P =<0.0001)	3.87±0.28	7.07±1.96
IVSTd (mm)	0.86±0.14	0.29±0.11$^{\$}$ (P =<0.0001)	0.70±0.11	0.33±0.07
LVPWd (mm)	0.95±0.28	1.13±0.41$^{\$}$ (P =0.2845)	0.89±0.29	1.38±0.46
LVIDs (mm)	1.66±0.24	5.04±0.56$^{\$}$ (P <0.0001)	1.83±0.23	4.33±0.81
LVIDd (mm)	3.20±0.25	5.52±0.83$^{\$}$ (P <0.0001)	3.35±0.39	4.94±0.90
%EF	85.0±3.34	31.83±6.63$^{\$}$ (P <0.0001)	82.75±1.98	41.83±5.78$^{\#}$ (P =0.0002)
%FS	48.0±4.07	12.75±3.02$^{\$}$ (P <0.0001)	45.75±1.98	17.42±2.94$^{\#}$ (P =0.0009)

Supplemental Table 2. Echocardiographic measurements of the hearts 14 or 28 days after sham or MI operation in mice administrated with ERG240 for 10 days.

IVSTd; interventricular septal thickness diastolic, LVIDd; left ventricular inner diameter diastolic, LVPWd; left ventricular posterior wall diameter diastolic, LVIDs; left ventricular inner diameter systolic, %EF; percent ejection fraction, %FS; percent fractional shortening. One-way ANOVA with Tukey's test was used for statistical analyses.

$^{\$}$ sham vs MI-water, $^{\#}$ MI-water vs MI-250mg/kg ERG240, ## MI-water vs MI-500mg/kg ERG240

day14

parameters	sham (*n* =9)	MI-water (*n* =5)	MI-250mg/kg ERG240 (*n* =5)	MI-500mg/kg ERG240 (*n* =5)
IVSTd (mm)	0.744±0.19	0.42±0.13$^{\$}$ (*P* =0.0062)	0.40±0.07	0.34±0.13
LVPWd (mm)	0.867±0.20	0.76±0.33$^{\$}$ (*P* =0.4597)	1.08±0.42	0.92±0.25
LVIDs (mm)	1.50±0.18	4.06±0.71$^{\$}$ (*P* <0.0001)	3.54±0.89	3.36±0.98
LVIDd (mm)	3.41±0.43	4.82±0.81$^{\$}$ (*P* =0.0010)	4.56±0.96	4.52±1.06
%EF	91.00±1.58	39.20±6.76$^{\$}$ (*P* <0.0001)	52.60±8.76$^{\#}$ (*P* =0.0268)	58.00±9.27$^{\#\#}$ (*P* =0.0064)
%FS	56.40±2.65	16.40±3.21$^{\$}$ (*P* <0.0001)	23.20±4.87$^{\#}$ (*P* =0.0312)	26.60±5.46$^{\#\#}$ (*P* =0.0070)

day28

parameters	sham (*n* =9)	MI-water (*n* =5)	MI-250mg/kg ERG240 (*n* =5)	MI-500mg/kg ERG240 (*n* =5)
IVSTd (mm)	0.74±0.18	0.26±0.09$^{\$}$ (*P* =0.0010)	0.36±0.05	0.42±0.13
LVPWd (mm)	1.00±0.38	1.58±0.40$^{\$}$ (*P* =0.0201)	1.18±0.15	1.28±0.39
LVIDs (mm)	1.63±0.20	4.22±0.46$^{\$}$ (*P* <0.0001)	3.78±0.92	3.50±1.17
LVIDd (mm)	3.36±0.41	4.72±0.42$^{\$}$ (*P* <0.0001)	4.74±1.01	4.60±1.29
%EF	87.67±0.87	26.60±5.13$^{\$}$ (*P* =0.0005)	48.40±5.94$^{\#}$ (*P* =0.0079)	55.40±7.23$^{\#\#}$ (*P* <0.0001)
%FS	51.56±1.59	10.40±2.19$^{\$}$ (*P* <0.0001)	20.60±2.61$^{\#}$ (*P* =0.0079)	24.60±3.97$^{\#\#}$ (*P* =0.0001)

Supplemental Table 3. Echocardiographic measurements of the hearts 28 days after MI operation in mice administrated with ERG240 starting at day 7 post-MI for 7 days.

IVSTd; interventricular septal thickness diastolic, LVIDd; left ventricular inner diameter diastolic, LVPWd; left ventricular posterior wall diameter diastolic, LVIDs; left ventricular inner diameter systolic, %EF; percent ejection fraction, %FS; percent fractional shortening. Unpaired t test was used for statistical analyses.

MI-water vs MI-250mg/kg ERG240

day28

parameters	MI-water (n=5)	MI-250mg/kg ERG240 (n=6)
IVSTd (mm)	0.24±0.09	0.30±0.09
LVPWd (mm)	1.16±0.29	1.02±0.38
LVIDs (mm)	4.50±0.64	4.38±0.53
LVIDd (mm)	5.18±0.74	5.35±0.69
%EF	33.20±4.02	43.00±1.90# (P=0.0005)
%FS	13.2±1.48	18.00±0.89# (P<0.0001)

Supplemental Table 4. List of qRT-PCR primer and probe sets used in this study

qRT-PCR

Gene Name	Species	Company name	Catalogue number	Forward	Rerverse	Probe
Bcat1	mouse	Sigma-Aldrich	Custom	5'-CGAGAGGGGAAAAAGGACCCA-3'	5'-TGGTCCTACAACTCCACCTTCTC-3'	5'-AGGTGCTATTGGTTTCCCTGGACCCA-3'
Acta2	mouse	Sigma-Aldrich	Custom	5'-CACCATGAAGATCAAGATCATTGCC-3'	5'-GGTAGACAGCGAAGCCAGGA-3'	5'-AGCCACCGATCCAGACAGAGTACTTGCG-3'
Gapdh	mouse	Sigma-Aldrich	Custom	5'-CGTCCCGTAGACAAAATGGTGA-3'	5'-CCACTTTGCCACTGCAAATGG-3'	5'-CCAATACGGCCAAATCCGTTCACACCGA-3'
18S rRNA	mouse	Sigma-Aldrich	Custom	5'-GGGTCATAAGCTTGCGTTGATTAAG-3'	5'-TCCGAGGGCCTCACTAAACC-3'	5'-TACACACCGCCCGTCGCTACTACCG-3'
Mrtfa	mouse	Sigma-Aldrich	Custom	5'-CCCACATCTCTGCTGAAGAAGG-3'	5'-GACTGGAGGAGCCATTTTCCTG-3'	5'-AGGCTGCTGGGTCACAGTCTCTTCATAAC-3'
Mrtfb	mouse	Sigma-Aldrich	Custom	5'-AGCCAGCAAGCCTAGCTCA-3'	5'-GGATCTTTTGTCTTCGTGATTGGG-3'	5'-CCCAGCAGTTTGTTGTTCAGCACTCTTTGT-3'
Yap1	mouse	Sigma-Aldrich	Custom	5'-GAACCAGAGAATCAGTCAGAGTG-3'	5'-CGCAGCCTCTCCTTCTCCA-3'	5'-TCCAGTGAAACAGCCACCACCCCTG-3'
Ctgf	mouse	Sigma-Aldrich	Custom	5'-GGACCGCACAGCAGTTGG-3'	5'-GGCAGTTGGCTCGCATCATA-3'	5'-CCTAGCTGCCTACCGACTGGAAGACACATT-3'
Cyr61	mouse	Sigma-Aldrich	Custom	5'-CGGGAGTCGGGCTGGTCC-3'	5'-GGTGTGGTCGCAGGGCTG-3'	5'-CGCAGACCTTACAGCAGCCGCAGC-3'
Bcat2	mouse	Sigma-Aldrich	Custom	5'-GTGTCCTCCATATTCAAGGCTG-3'	5'-CTGTGAAGGTCTTCCCAAACA-3'	5'-ACCTCCAGATTCAGATGACCAAAGAACCAC -3'
Bckdha	mouse	Sigma-Aldrich	Custom	5'-AGGGTTGGTGGGATGAGGAA-3'	5'-CTTCCGCTCAGCCTGCTC-3'	5'-ATGACCTTCTTTCGTGACTGCTTCCGCC-3'
Bckdhb	mouse	Sigma-Aldrich	Custom	5'-GGAACAGGTCCCAGTAGAACC-3'	5'-CCCGGATGACATGAACCTGAG-3'	5'-CCCAGGCAACCAGAGTCACATCGCT-3'
Collagen 1a1	mouse	Sigma-Aldrich	Custom	5'-CCCAAAGGTTCTCCTGGTGAAG-3'	5'-CGGTTTTGCCATCAGGACCA-3'	5'-TGGTGCCAAGGGTCTCACTGGCAGTC-3'
Collagen 1a2	mouse	Sigma-Aldrich	Custom	5'-CAAGCATGTCTGGTTAGGAGAGA-3'	5'-GCTGAGTTGCCATTTCCTTGG-3'	5'-CCCTTCTACGTTGTATTCAAACTGGCTGCC-3'
Tgfb1	mouse	Sigma-Aldrich	Custom	5'-GGAACTCTACCAGAAATATAGCAACA-3'	5'-AAGCCCTGTATTCCGTCTCC-3'	5'-TGGTTCAGCCACTGCCGTACAACTCC-3'
Snai1	mouse	Sigma-Aldrich	Custom	5'-CGCACCCACACTGGTGAG-3'	5'-CACATCCGAGTGGGTTTGGA-3'	5'-TTGCTGACCGCTCCAACCTGCGT-3'
Aldh18a1	mouse	Sigma-Aldrich	Custom	5'-GGTCACCCGACTCGTCAGA-3'	5'-CAGCAGATCTCGGTGGATAAGC-3'	5'-AAATGTGAATACCCAGCCGCCTGTAATGCC-3'
Pycr1	mouse	Sigma-Aldrich	Custom	5'-ATGCCTTGCATGTGCTGGAG-3'	5'-TGCAGCTCCCGTGTACGG-3'	5'-CGCTCCTTGCTTATCAACGCTGTGGAAG-3'
Eprs	mouse	Sigma-Aldrich	Custom	5'-CGCTACCTGGCTAGAATTGCA-3'	5'-ACTCCAACCAATGATCAATCTCAG-3'	5'-CTCCATCAGGTTAGTCCCATACAGCCCAGA-3'
Cd45	mouse	Sigma-Aldrich	Custom	5'-TCAGGACATCGTTTGTGGTCA-3'	5'-ATGTGAAGAAGGAAACAGGAACAAG-3'	5'-ACAACAATATCTTTGAAAGCCCGAGTGCCT-3'
Cd68	mouse	Sigma-Aldrich	Custom	5'-CCGCTTATAGCCCAAGGAACA-3'	5'-TTCTGTGGCTGTAGGTGTCATC-3'	5'-AAAGGCCGTTACTCTCCTGCCATCCT-3'
Il1b	mouse	Sigma-Aldrich	Custom	5'-TGCCACCTTTTGACAGTGATGA-3'	5'-GCCCAGGTCAAAGGTTTGGAA-3'	5'-ACCTGTTCTTTGAAGTTGACGGACCCCA-3'
Il6	mouse	Sigma-Aldrich	Custom	5'-TGGAAATGAGAAAAGAGTTGTGC-3'	5'-TCCAGTTTGGTAGCATCCATCA-3'	5'-TTCTGCAAGTGCATCATCGTTGTTCATACA-3'
Ccl2	mouse	Sigma-Aldrich	Custom	5'-CGGCTGGAGCATCCACGT-3'	5'-ATTGGGATCATCTTGCTGGTGAAT-3'	5'-TCAGCCAGATGCAGTTAACGCCCCAC-3'
Cxcl2	mouse	Sigma-Aldrich	Custom	5'-AGGGTGAGTTGGGAACTAGCTA-3'	5'-CTCTCCTCGGTGCTTACAAAA-3'	5'-CACTCCTTCCATGAAAGCCATCCGACTGC-3'
Tnf	mouse	Sigma-Aldrich	Custom	5'-GTCTCAGCCTCTTCTCATTCCTG-3'	5'-GAGGCCATTTGGGAACTTCTCA-3'	5'-AGGGGCCACCACGCTCTTCTGTCTAC-3'
BCAT1	human	Sigma-Aldrich	Custom	5'-TCTGCTAGTCTGTATATTCGTCCTAC-3'	5'-ACTGGATTAAAGGTTCCACTTGAAA-3'	5'-TCATTGGAACTGAGCCTTCTCTTGGAGTCA-3'
ACTA2	human	Sigma-Aldrich	Custom	5'-GATCCTGACTGAGCGTGGC-3'	5'-CAGAGCTACATAACACAGTTTCTCC-3'	5'-ATTCCTTCGTTACTACTGCTGAGCGTGAGA-3'
Collagen 1a1	human	Sigma-Aldrich	Custom	5'-ACTGGTGACCTGCGTGTA-3'	5'-GCCGCATACTCGAACTGG-3'	5'-CTCTCGCCAACCAGACATGCCTCTTG-3'
GAPDH	human	Sigma-Aldrich	Custom	5'-TGGGCTACACTGAGCACCAG-3'	5'-GCCAAATTCGTTGTCATACCAGG-3'	5'-TTCAACAGCGACACCCACTCCTCCACC-3'

Supplemental Table 5. The rist of siRNAs used in this study

siRNA	Company	Assay ID	Species
si*Mrtfa*	ambion	s104594	mouse
si*Mrtfb*	ambion	s109075	mouse
si*Yap1*	ambion	s76159	mouse
si*Bcat1* #1	ambion	s62889	mouse
si*Bcat1* #2	ambion	s62890	mouse
si*BCAT1*	ambion	s1900	human
si*Smad3*	ambion	s69495	mouse
si*Aldh18a1*	ambion	4390771	mouse
si*Pycr1*	ambion	s102002	mouse
si*Eprs*	ambion	s232849	mouse
si*Hdac5* #1	ambion	s67423	mouse
si*Hdac5* #2	ambion	s67424	mouse
siCtrl	ambion	4390844	mouse/human

Supplemental Table 6. List of antibodies and reagents used in this study

Antibody	Company name	Dilution	Catalogue number	Clone number
anti-BCAT1	Invitrogen	1:4000	Cat# MA5-25892	OTI3F5
anti-αSMA	Thermo	1:5000	Cat# MS-113-P	1A4
anti-GAPDH	Santa Cruz	1:10000	Cat# sc-32233	6C5
anti-ALDH18A1	Proteintech	1:4000	Cat# 17719-1-AP	
anti-PYCR1	Proteintech	1:4000	Cat# 13108-1-AP	
anti-EPRS	Proteintech	1:4000	Cat# 67712-1-lg	1B7G2
anti-Collagen 1a1	CST	1:4000	Cat# 72026	E8F4L
anti-SMAD3	CST	1:4000	Cat# 8685P	D7G7
anti-P-SMAD3 (Ser423/425)	CST	1:4000	Cat# 9520P	C25A9
anti-P-mTOR	Proteintech	1:4000	Cat# 67778-1-Ig	2A12G3
anti-mTOR	Proteintech	1:4000	Cat# 66888-1-Ig	1G11A3
anti-P-TFEB	CST	1:4000	Cat# 87932	E9M5M
anti-TFEB	CST	1:4000	Cat# 83010	E5P9M
anti-P-GCN2	abcam	1:4000	Cat# ab75836	EPR2320Y
anti-GCN2	CST	1:4000	Cat# 3302	
anti-HDAC5	CST	1:4000	Cat# 20458	D1J7V
anti-P-HDAC4 (Ser 629)/P-HDAC5 (Ser488)	CST	1:4000	Cat# 3424	
anti-Collagen 6a1	CST	1:4000	Cat# 58232	E9Q3P
anti-β-tubulin	Fujifilm	1:4000	Cat# 014-25041	10G10
anti-HA-peroxidase conjugated	Sigma-Aldrich	1:4000	Cat# 12013819001	3F10
anti-mouse IgG-HRP	Abcam	1:10000	Cat# ab6789	
anti-mouse IgG-HRP-linked Antibody	CST	1:2000	Cat# 7076	
anti-rabbit IgG, HRP-linked Antibody	CST	1:2000	Cat# 7074	

Reagent	Company name	Concentration	Catalogue number
DAPI	Dojindo	1:1000	Cat# 340-07971
Latrunculin A	Abcam	2 μM	Cat# ab144290
BTT-3033	Tocris	30 μM	Cat# 4724
recombinant TGF-β1	R&D Systems	5 ng/mL	Cat# 240-B
TNF-α	Peprotech	10 ng/mL	Cat# 315-01A-20UG
LPS	Sigma-Aldrich	100 ng/mL	Cat# L4391
Proline	Nacalai Tesque	1 mM	Cat# 13040-22
BCAAs	Nacalai Tesque	1 mM	Cat# 13046-62, 13036-92, 13035-02

Immunohistochemistry

Antibody	Company name	Dilution	Catalogue number	Clone number
anti-Desmin	Abcam	1:200	Cat# ab32362	Y66
anti-TNNI3	Abcam	1:200	Cat# ab56357	
anti-CD31	BD Biosciences	1:200	Cat# 550274	MEC 13.3
anti-CD45	BioLegend	1:200	Cat# 103101	30-F11
anti-Collagen 1a1	CST	1:200	Cat# 72026	E8F4L
anti-αSMA	Abcam	1:200	Cat# ab5694	
AlexaFluor®488-conjugated donkey anti-rabbit IgG	Invitrogen	1:200	Cat# A21206	
AlexaFluor®488-conjugated donkey anti-goat IgG	Abcam	1:200	Cat# ab150129	
AlexaFluor®488-conjugated donkey anti-rat IgG	Invitrogen	1:200	Cat# A21208	
AlexaFluor®647-conjugated donkey anti-rabbit IgG	Invitrogen	1:200	Cat# A21244	

RNAscope

Gene	Species	Company name	Catalogue number
Negative probe	E.coli	ACD	Cat# 310034
Bcat1	mouse	ACD	Cat# 824231
Col1a1	mouse	ACD	Cat# 319371-C3
Postn	mouse	ACD	Cat# 418581-C2
Snai1	mouse	ACD	Cat# 451211-C2
TSA Plus Cyanine 3		PerkinElmer	Cat# NEL753001KT
TSA Plus Cyanine 5		PerkinElmer	Cat# NEL745001KT

Supplemental Table 7. List of ChIP-qPCR primer sets used in this study

ChIP-qPCR

Gene Name	Species	Company name	Catalogue number	Forward	Rerverse
Aldh18a1	mouse	Eurofins	Custom	5'-GGTTTGCAGAAGAGAACATCTGGC-3'	5'-CCCAGTTTCTACACCTCTGTGCTG-3'
Pycr1	mouse	Eurofins	Custom	5'-GCTGTGGGTAGCGGGACTTGGGGC-3'	5'-GATTGATTGAAGGGGAGGGTCAGG-3'
Eprs	mouse	Eurofins	Custom	5'-TGCGGTGCCTTCGGTCGCGCGCAC-3'	5'-TCCGGCGTTCACCGTGAGGCACAG-3'
