# Supplementary material for: Branched-chain amino acid transaminase 1–mediated pathway promotes proline-dependent collagen production in cardiac myofibroblasts
Source: J Clin Invest. 2026 Jul 16;136(17):e182216. doi: 10.1172/JCI182216 (PMC13528941; doi:10.1172/JCI182216)
Supplement: Supplementary file 2 [file jci-136-182216-s287.pdf]

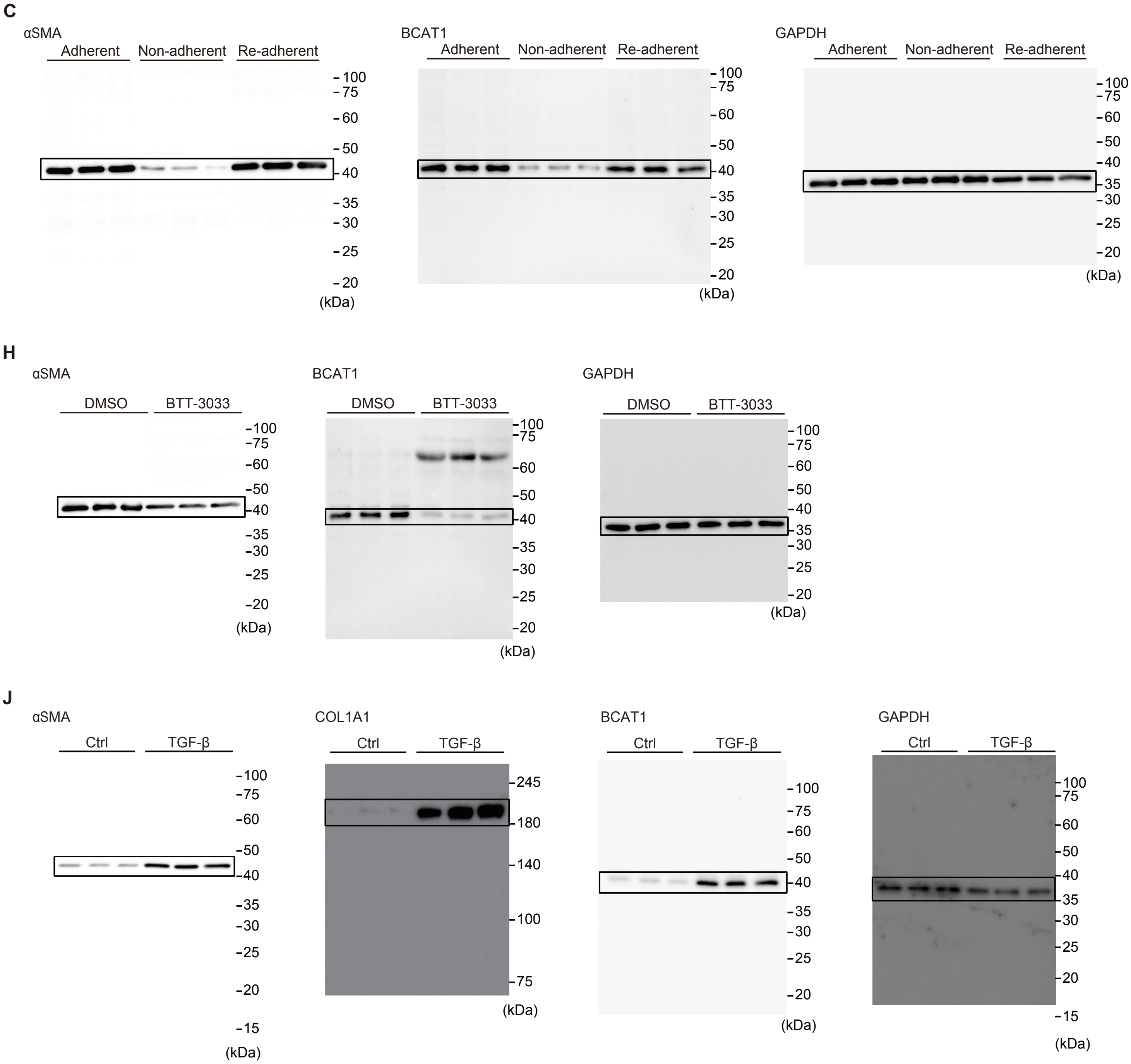
C
αSMA
Adherent
Non-adherent
Re-adherent
100
75
60
50
40
35
30
25
20
(kDa)
BCAT1
Adherent
Non-adherent
Re-adherent
GAPDH
Adherent
Non-adherent
Re-adherent
H
αSMA
DMSO
BTT-3033
BCAT1
DMSO
BTT-3033
GAPDH
DMSO
BTT-3033
J
αSMA
Ctrl
TGF-β
15
COL1A1
Ctrl
TGF-β
245
180
140
100
75
BCAT1
Ctrl
TGF-β
GAPDH
Ctrl
TGF-β

Figure 1

B

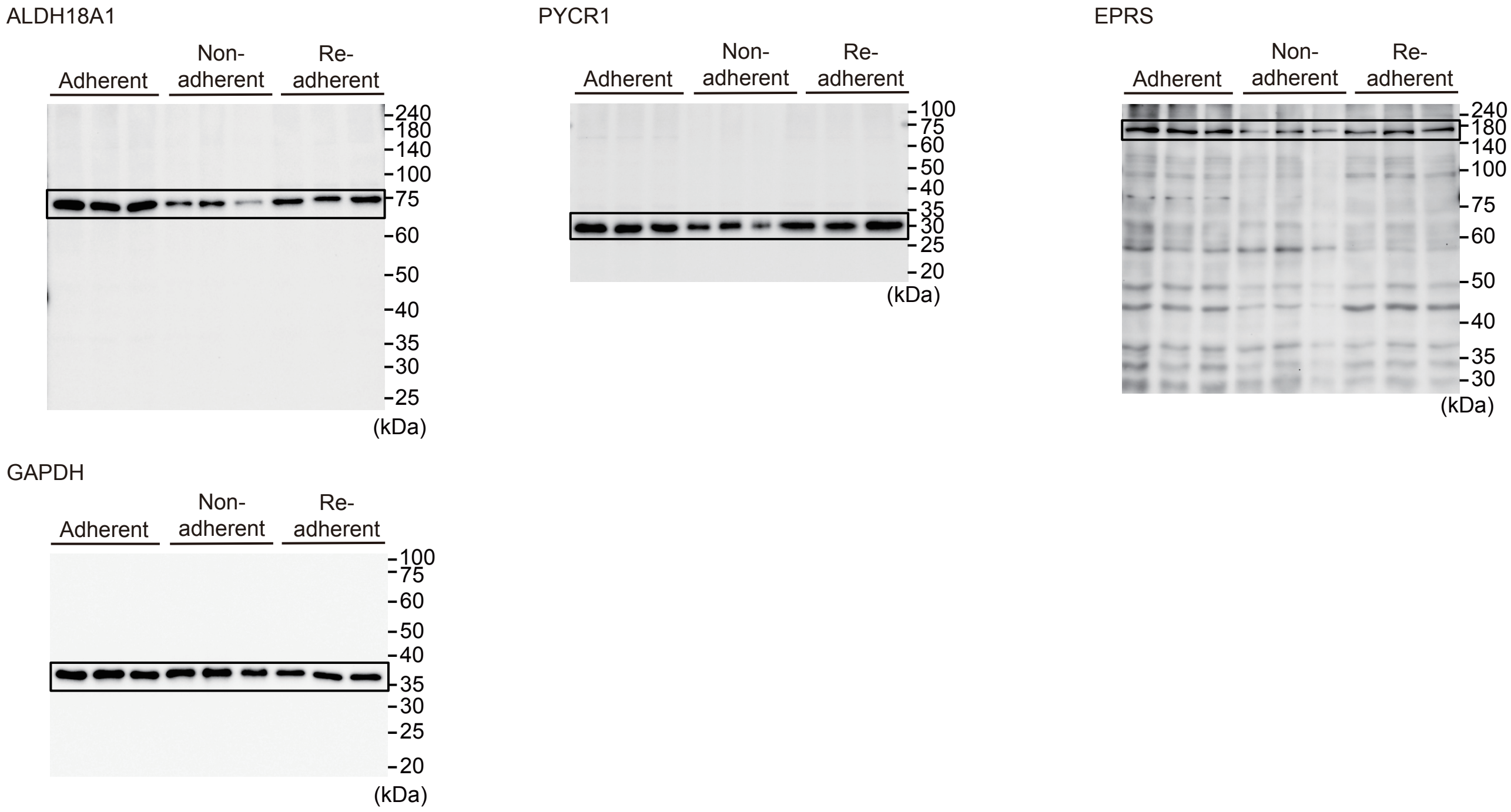

ALDH18A1
Adherent
Non-adherent
Re-adherent
240
180
140
100
75
60
50
40
35
30
25
(kDa)
PYCR1
Adherent
Non-adherent
Re-adherent
100
75
60
50
40
35
30
25
20
(kDa)
EPRS
Adherent
Non-adherent
Re-adherent
240
180
140
100
75
60
50
40
35
30
(kDa)
GAPDH
Adherent
Non-adherent
Re-adherent
100
75
60
50
40
35
30
25
20
(kDa)

D

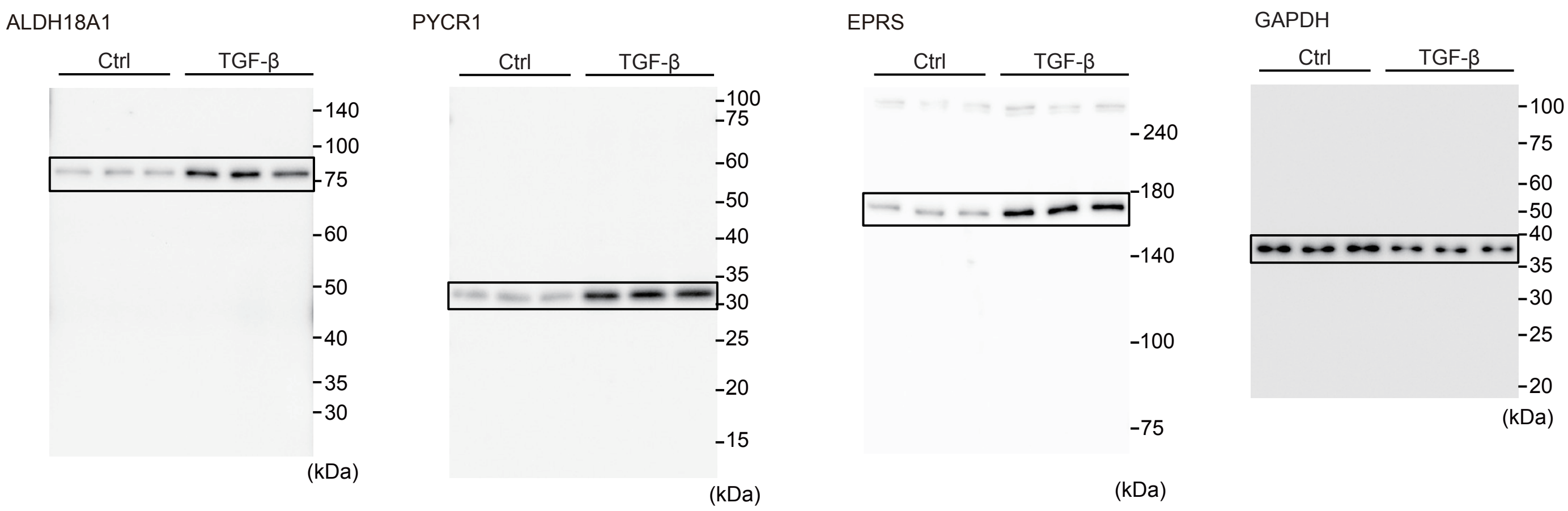

ALDH18A1
Ctrl
TGF-β
140
100
75
60
50
40
35
30
(kDa)
PYCR1
Ctrl
TGF-β
100
75
60
50
40
35
30
25
20
15
(kDa)
EPRS
Ctrl
TGF-β
240
180
140
100
75
(kDa)
GAPDH
Ctrl
TGF-β
100
75
60
50
40
35
30
25
20
(kDa)

Figure 4

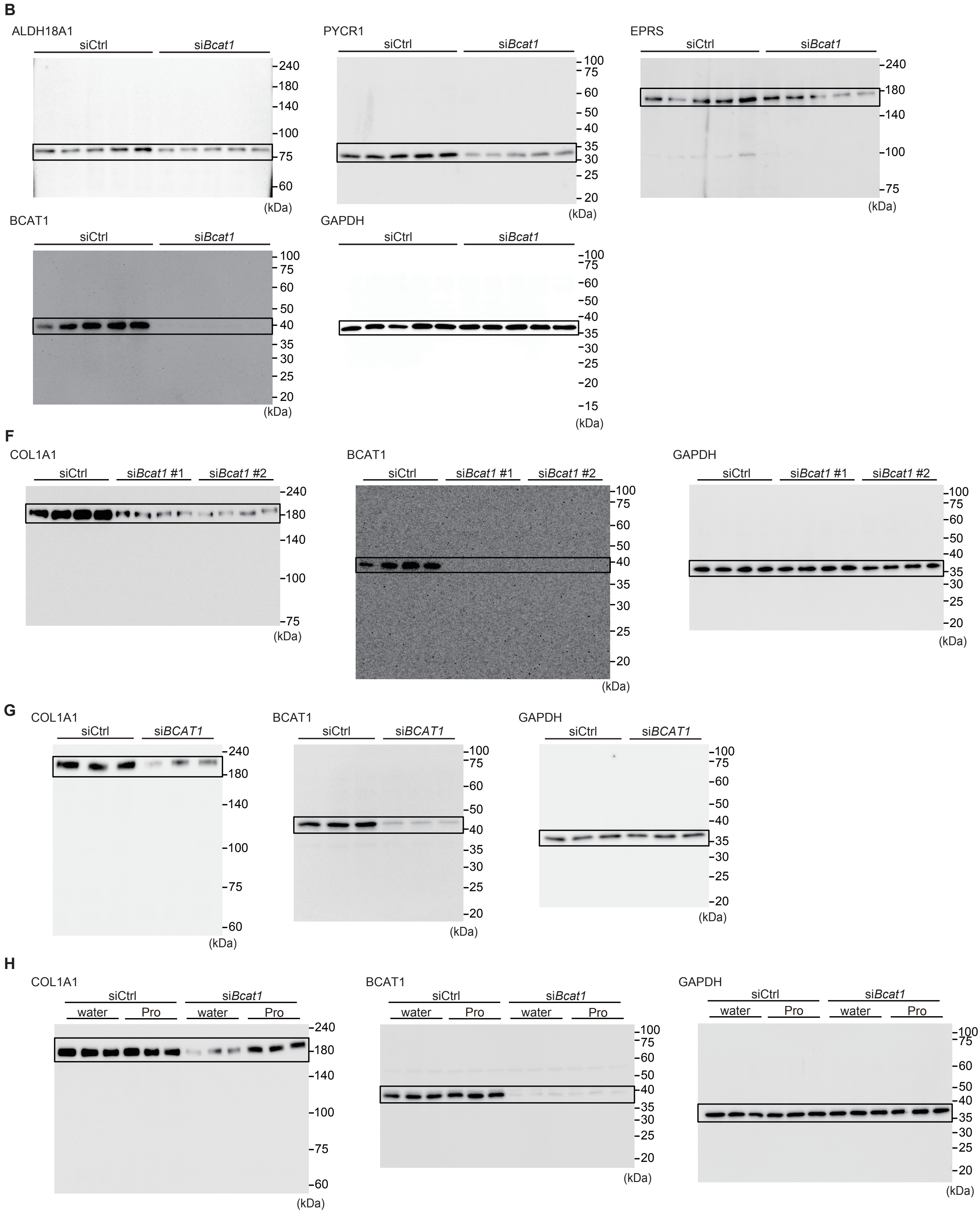
B
ALDH18A1
siCtrl
siBcat1
240
180
140
100
75
60
(kDa)
PYCR1
siCtrl
siBcat1
100
75
60
50
40
35
30
25
20
(kDa)
EPRS
siCtrl
siBcat1
240
180
140
100
75
(kDa)
BCAT1
siCtrl
siBcat1
100
75
60
50
40
35
30
25
20
(kDa)
GAPDH
siCtrl
siBcat1
100
75
60
50
40
35
30
25
20
15
(kDa)
F
COL1A1
siCtrl
siBcat1 #1
siBcat1 #2
240
180
140
100
75
(kDa)
BCAT1
siCtrl
siBcat1 #1
siBcat1 #2
100
75
60
50
40
35
30
25
20
(kDa)
GAPDH
siCtrl
siBcat1 #1
siBcat1 #2
100
75
60
50
40
35
30
25
20
(kDa)
G
COL1A1
siCtrl
siBCAT1
240
180
140
100
75
60
(kDa)
BCAT1
siCtrl
siBCAT1
100
75
60
50
40
35
30
25
20
(kDa)
GAPDH
siCtrl
siBCAT1
100
75
60
50
40
35
30
25
20
(kDa)
H
COL1A1
siCtrl
siBcat1
water
Pro
water
Pro
240
180
140
100
75
60
(kDa)
BCAT1
siCtrl
siBcat1
water
Pro
water
Pro
100
75
60
50
40
35
30
25
20
(kDa)
GAPDH
siCtrl
siBcat1
water
Pro
water
Pro
100
75
60
50
40
35
30
25
20
(kDa)

Figure 5

B

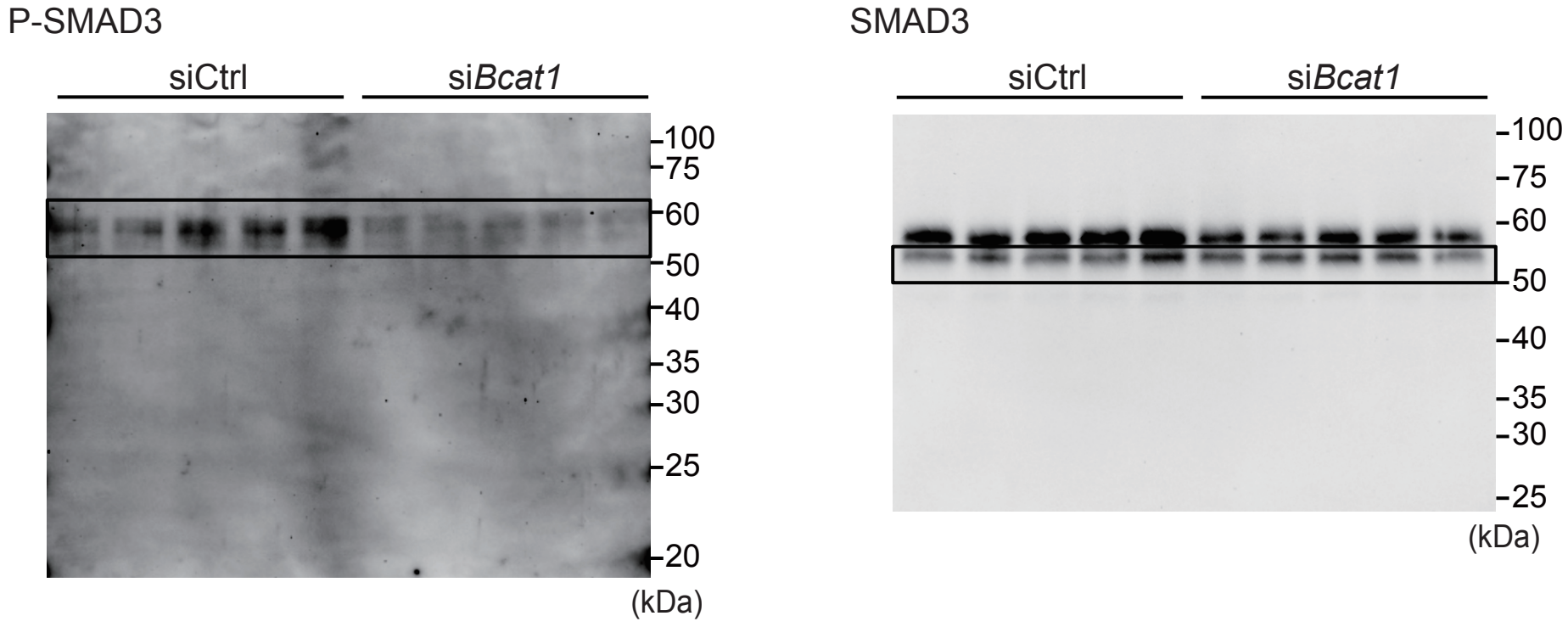

P-SMAD3
siCtrl
siBcat1
100
75
60
50
40
35
30
25
20
(kDa)
SMAD3
siCtrl
siBcat1
100
75
60
50
40
35
30
25
(kDa)

E

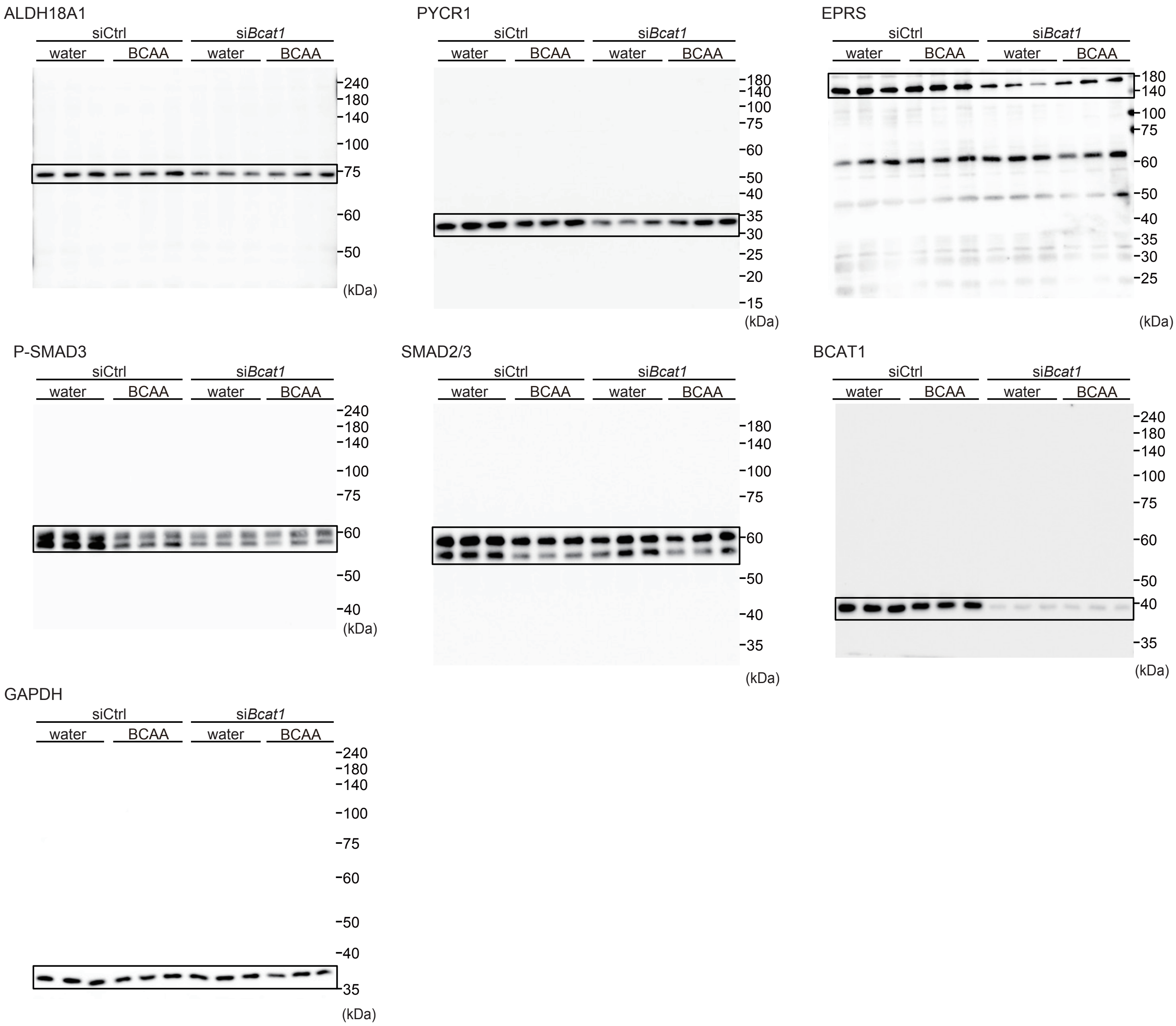

ALDH18A1
siCtrl
siBcat1
water
BCAA
water
BCAA
240
180
140
100
75
60
50
(kDa)
PYCR1
siCtrl
siBcat1
water
BCAA
water
BCAA
180
140
100
75
60
50
40
35
30
25
20
15
(kDa)
EPRS
siCtrl
siBcat1
water
BCAA
water
BCAA
180
140
100
75
60
50
40
35
30
25
(kDa)
P-SMAD3
siCtrl
siBcat1
water
BCAA
water
BCAA
240
180
140
100
75
60
50
40
(kDa)
SMAD2/3
siCtrl
siBcat1
water
BCAA
water
BCAA
180
140
100
75
60
50
40
35
(kDa)
BCAT1
siCtrl
siBcat1
water
BCAA
water
BCAA
240
180
140
100
75
60
50
40
35
(kDa)
GAPDH
siCtrl
siBcat1
water
BCAA
water
BCAA
240
180
140
100
75
60
50
40
35
(kDa)

Figure 6

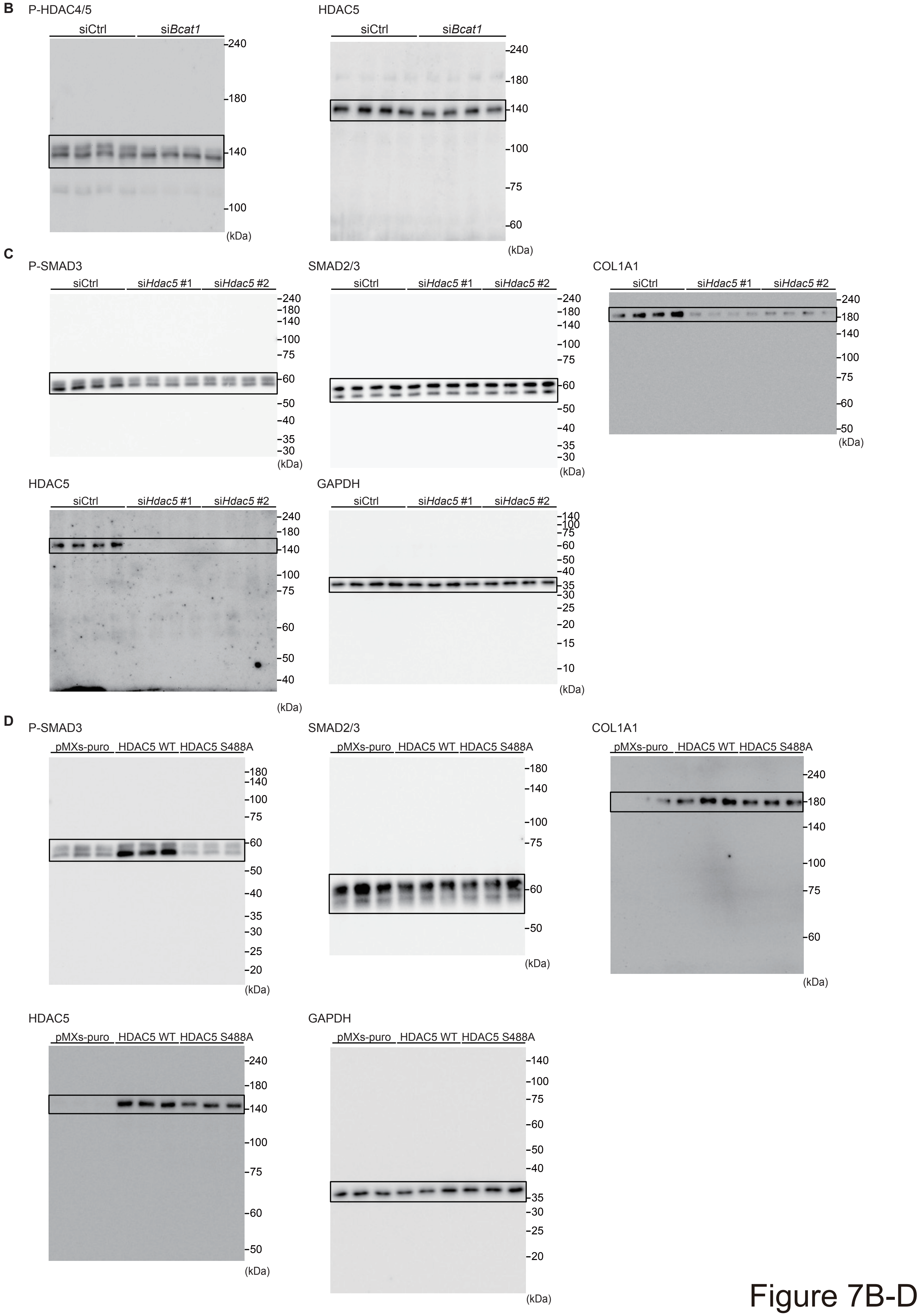
B
P-HDAC4/5
siCtrl
siBcat1
240
180
140
100
(kDa)
HDAC5
siCtrl
siBcat1
240
180
140
100
75
60
(kDa)
C
P-SMAD3
siCtrl
siHdac5 #1
siHdac5 #2
240
180
140
100
75
60
50
40
35
30
(kDa)
SMAD2/3
siCtrl
siHdac5 #1
siHdac5 #2
240
180
140
100
75
60
50
40
35
30
(kDa)
COL1A1
siCtrl
siHdac5 #1
siHdac5 #2
240
180
140
100
75
60
50
(kDa)
HDAC5
siCtrl
siHdac5 #1
siHdac5 #2
240
180
140
100
75
60
50
40
(kDa)
GAPDH
siCtrl
siHdac5 #1
siHdac5 #2
140
100
75
60
50
40
35
30
25
20
15
10
(kDa)
D
P-SMAD3
pMXs-puro
HDAC5 WT
HDAC5 S488A
180
140
100
75
60
50
40
35
30
25
20
(kDa)
SMAD2/3
pMXs-puro
HDAC5 WT
HDAC5 S488A
180
140
100
75
60
50
(kDa)
COL1A1
pMXs-puro
HDAC5 WT
HDAC5 S488A
240
180
140
100
75
60
(kDa)
HDAC5
pMXs-puro
HDAC5 WT
HDAC5 S488A
240
180
140
100
75
60
50
(kDa)
GAPDH
pMXs-puro
HDAC5 WT
HDAC5 S488A
140
100
75
60
50
40
35
30
25
20
(kDa)

Figure 7B-D

E

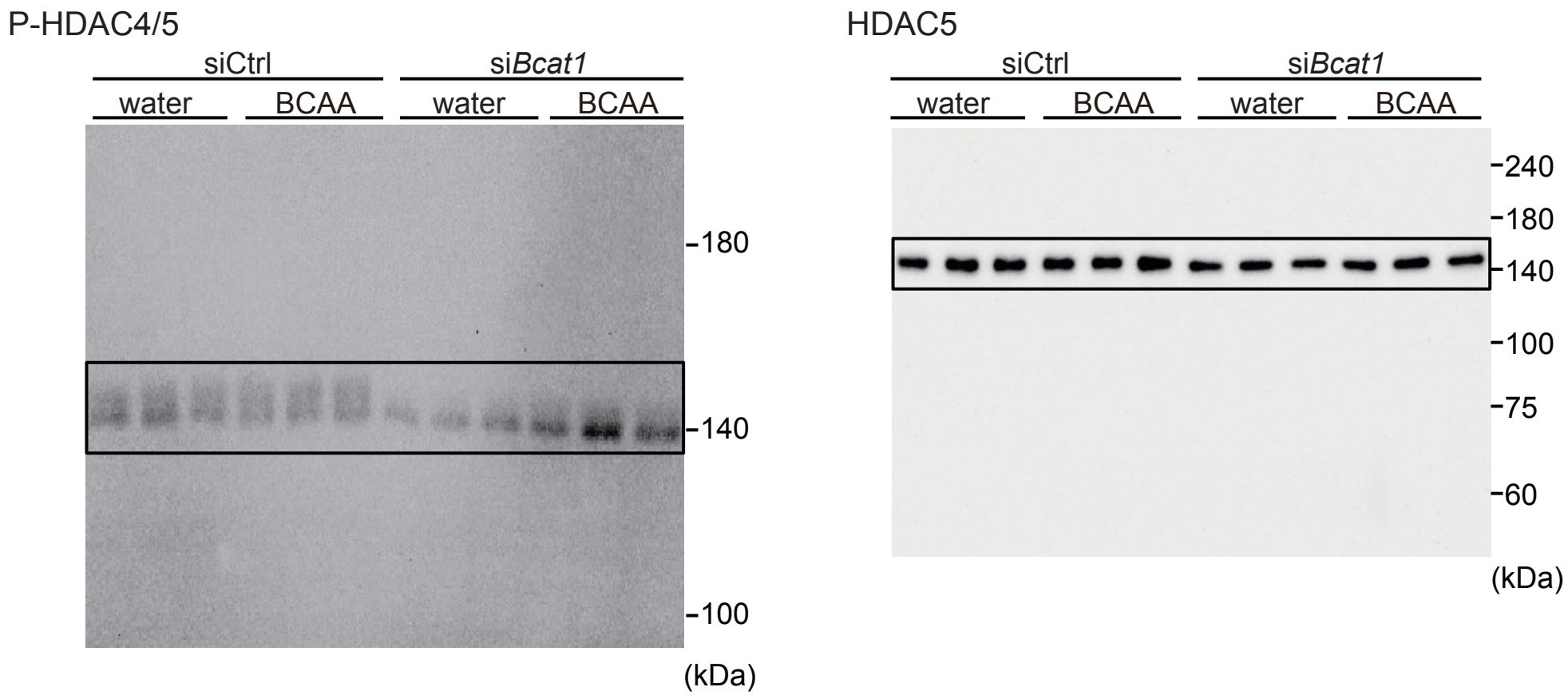
P-HDAC4/5
siCtrl
siBcat1
water
BCAA
water
BCAA
180
140
100
(kDa)
HDAC5
siCtrl
siBcat1
water
BCAA
water
BCAA
240
180
140
100
75
60
(kDa)

Figure 7E

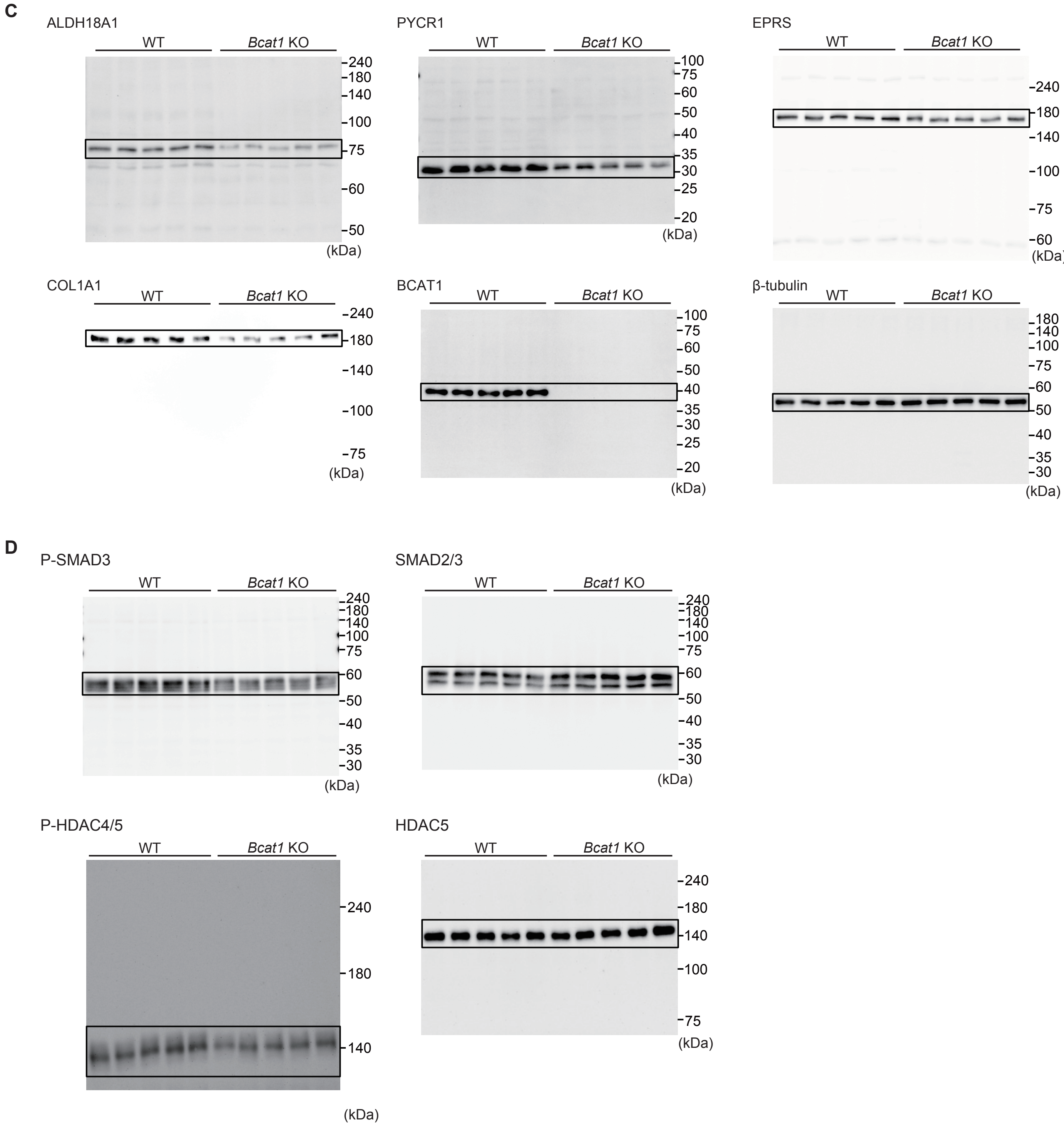
C
ALDH18A1
WT
Bcat1 KO
240
180
140
100
75
60
50
(kDa)
PYCR1
100
75
60
50
40
35
30
25
20
EPRS
240
180
140
100
75
60
COL1A1
BCAT1
β-tubulin
D
P-SMAD3
SMAD2/3
P-HDAC4/5
HDAC5

Figure 8

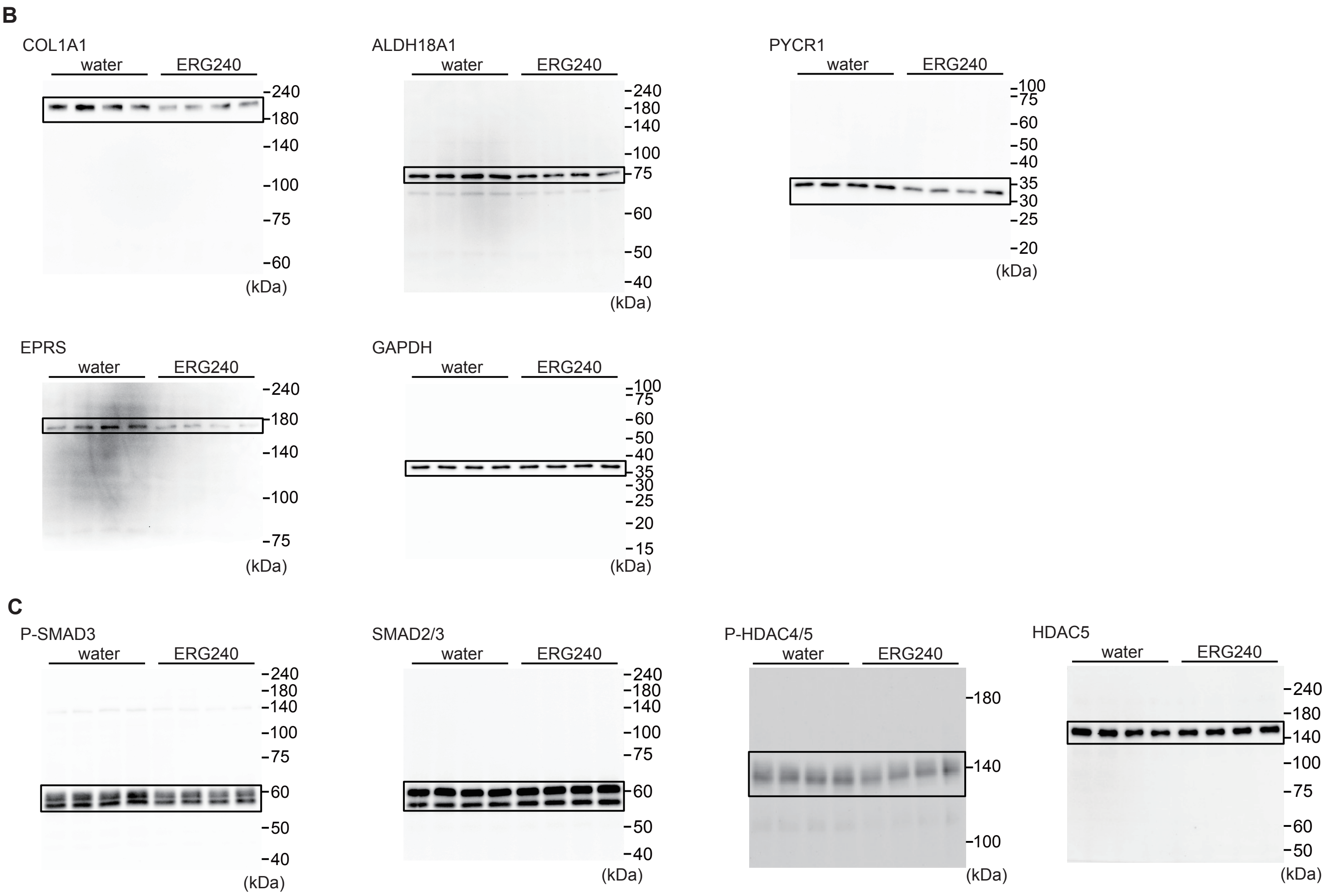
B
COL1A1
water
ERG240
240
180
140
100
75
60
(kDa)
ALDH18A1
water
ERG240
240
180
140
100
75
60
50
40
(kDa)
PYCR1
water
ERG240
100
75
60
50
40
35
30
25
20
(kDa)
EPRS
water
ERG240
240
180
140
100
75
(kDa)
GAPDH
water
ERG240
100
75
60
50
40
35
30
25
20
15
(kDa)
C
P-SMAD3
water
ERG240
240
180
140
100
75
60
50
40
(kDa)
SMAD2/3
water
ERG240
240
180
140
100
75
60
50
40
(kDa)
P-HDAC4/5
water
ERG240
180
140
100
(kDa)
HDAC5
water
ERG240
240
180
140
100
75
60
50
(kDa)

Figure 9

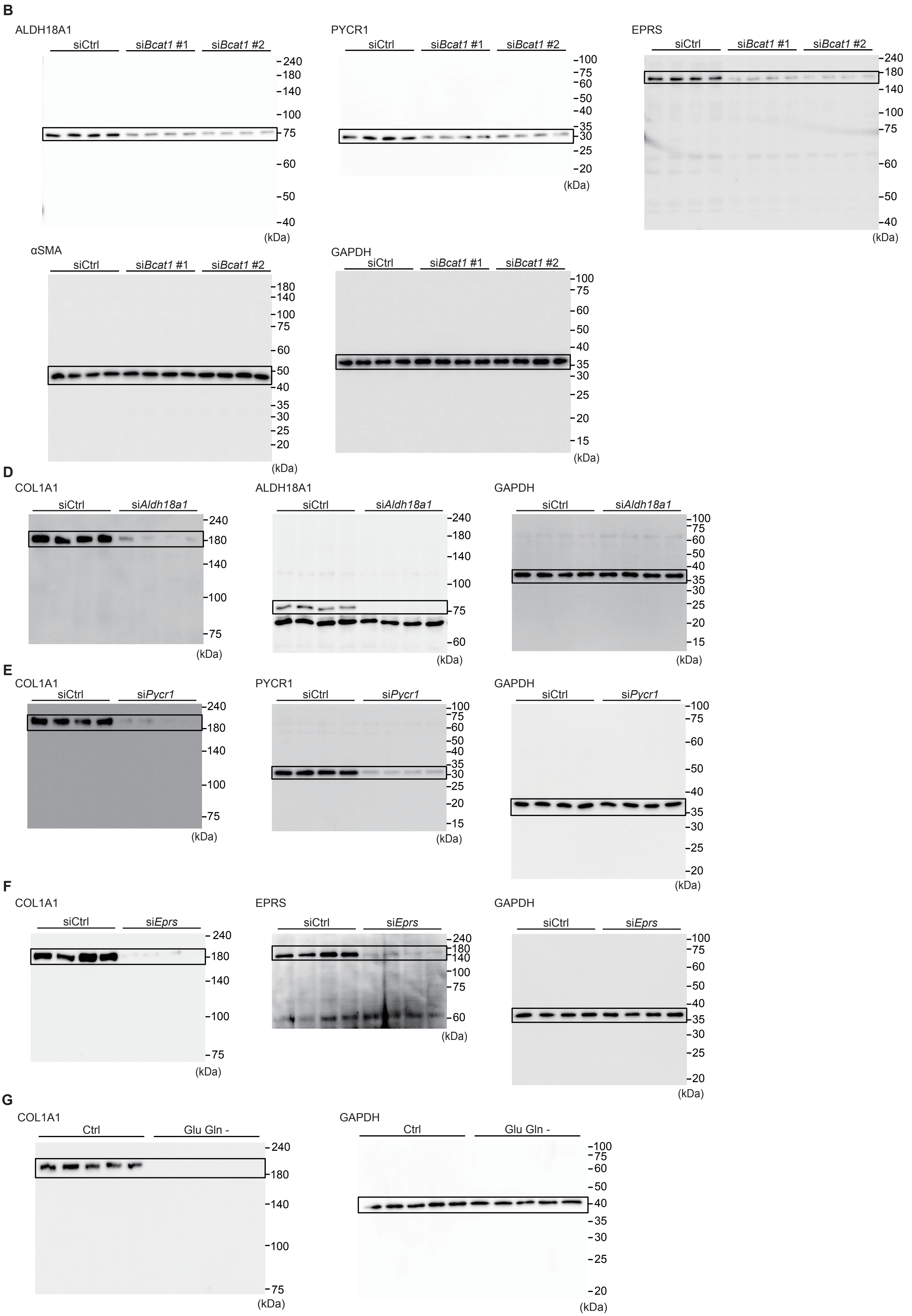

Supplemental Figure 4 B-G

H

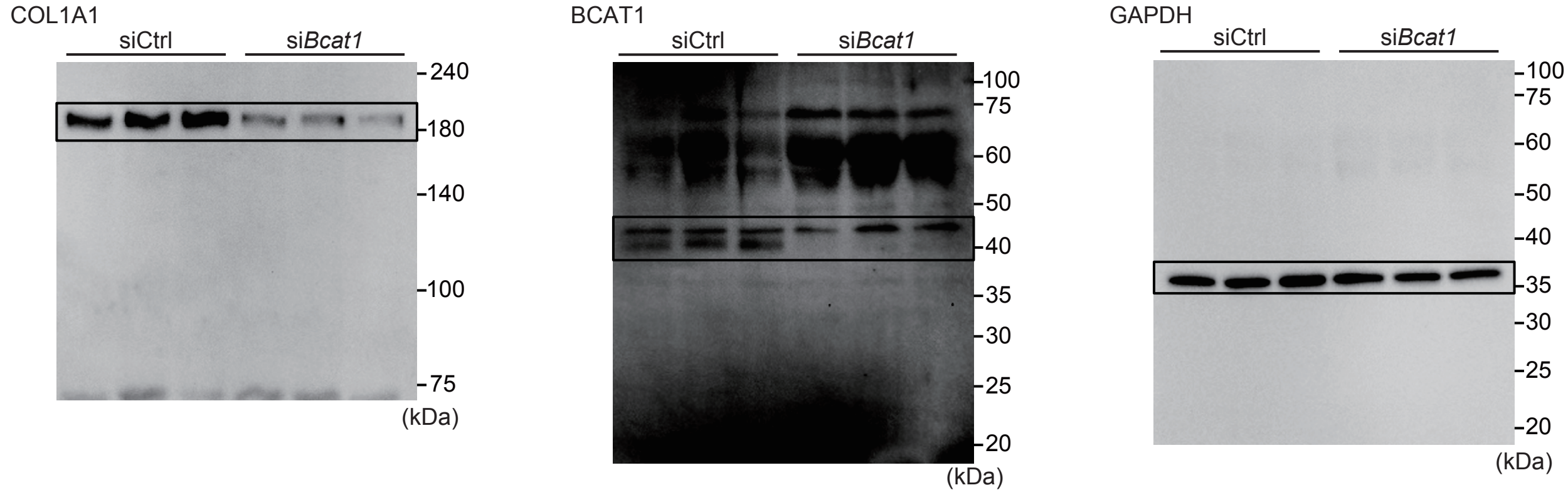

I

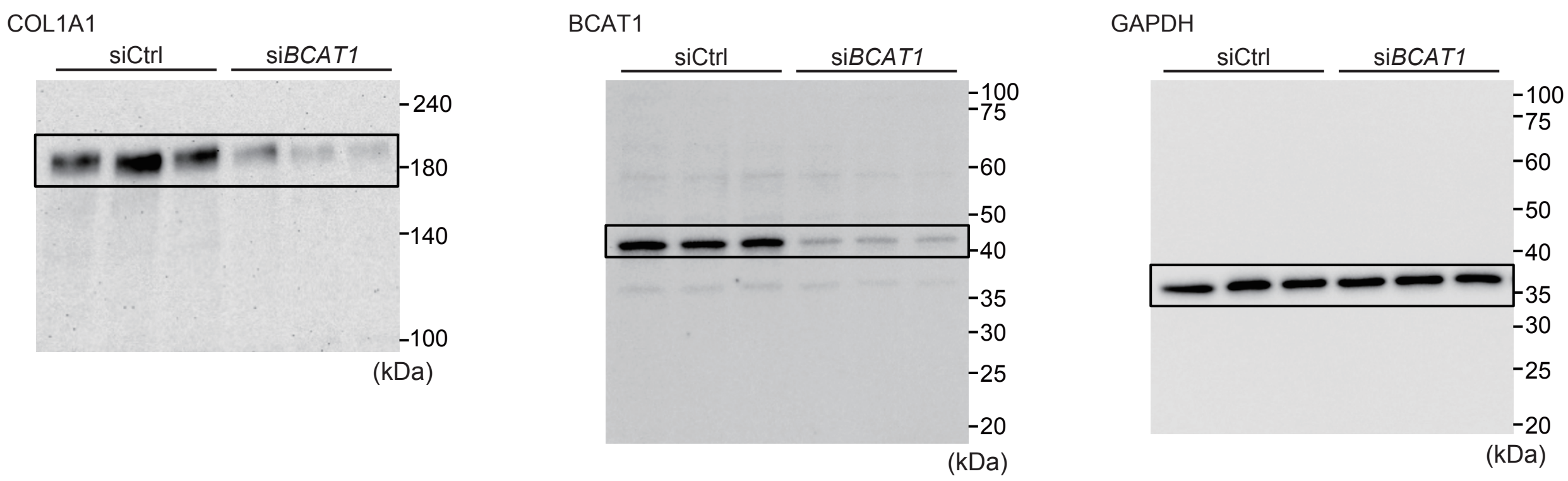

J

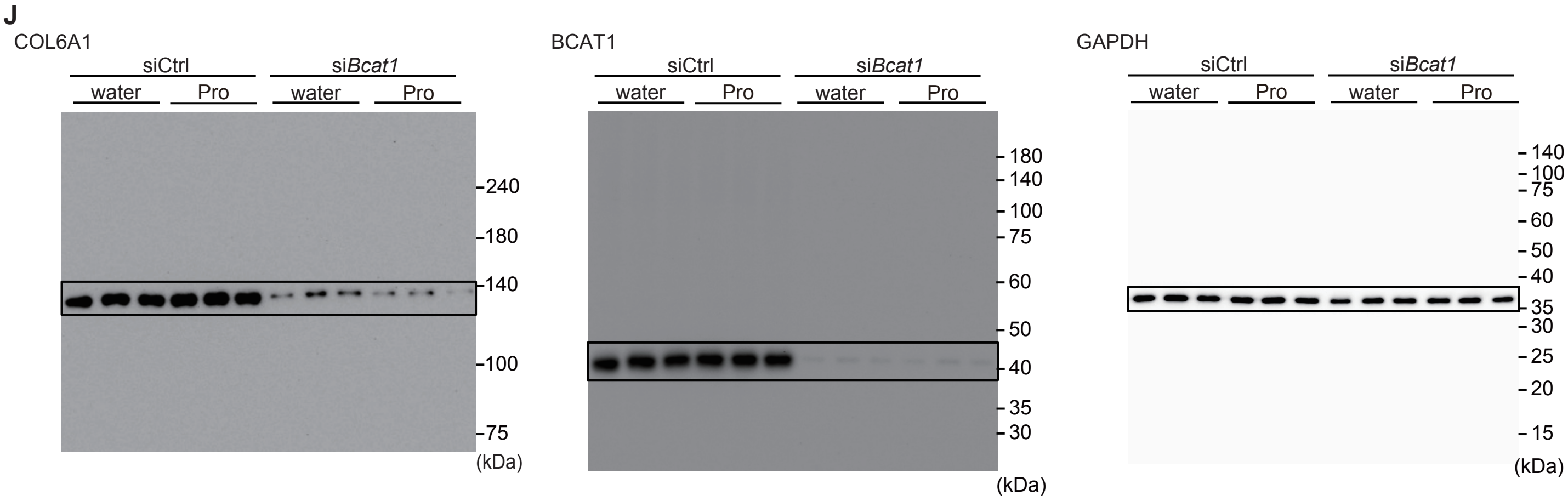

Supplemental Figure 4 H-J

A

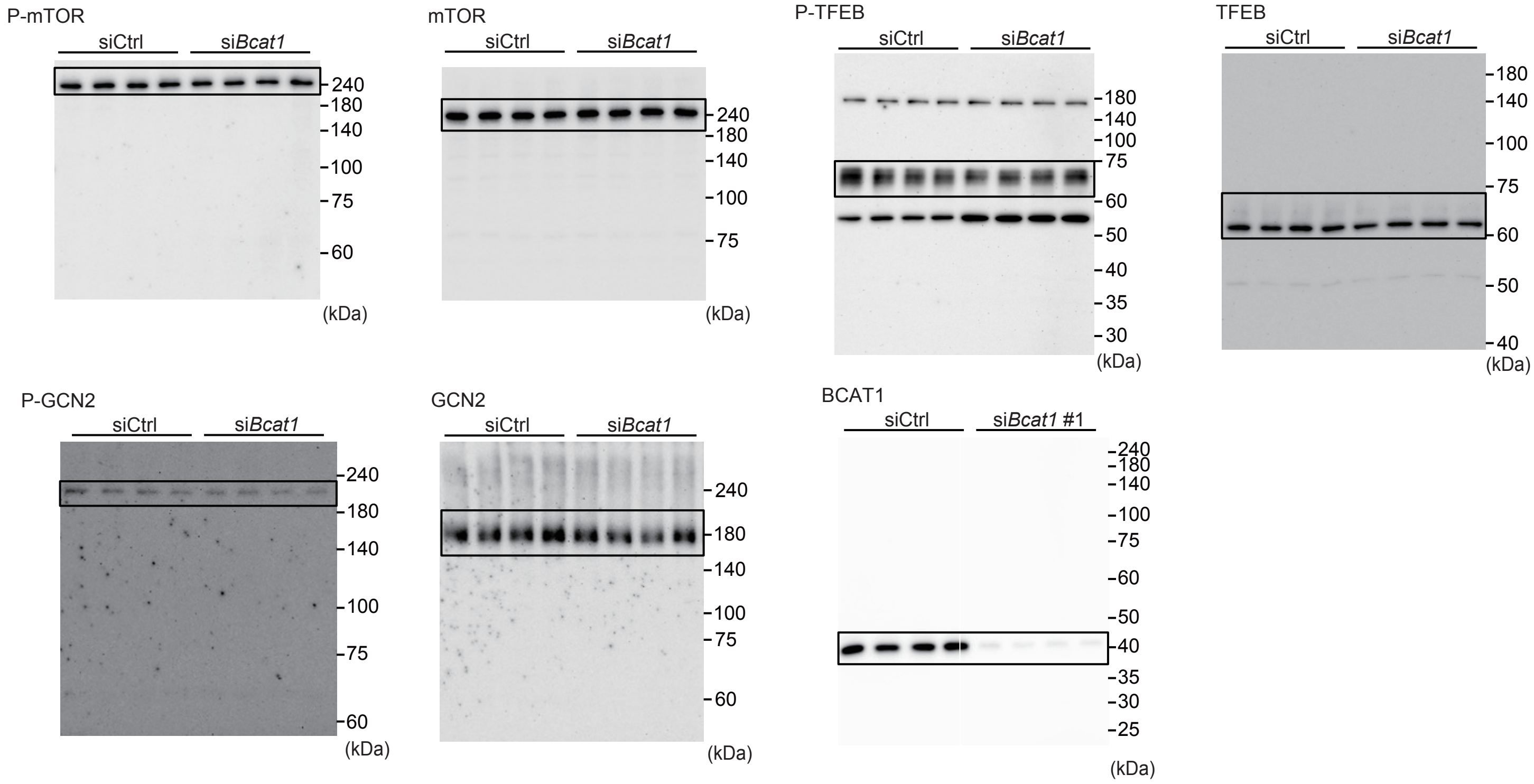

D

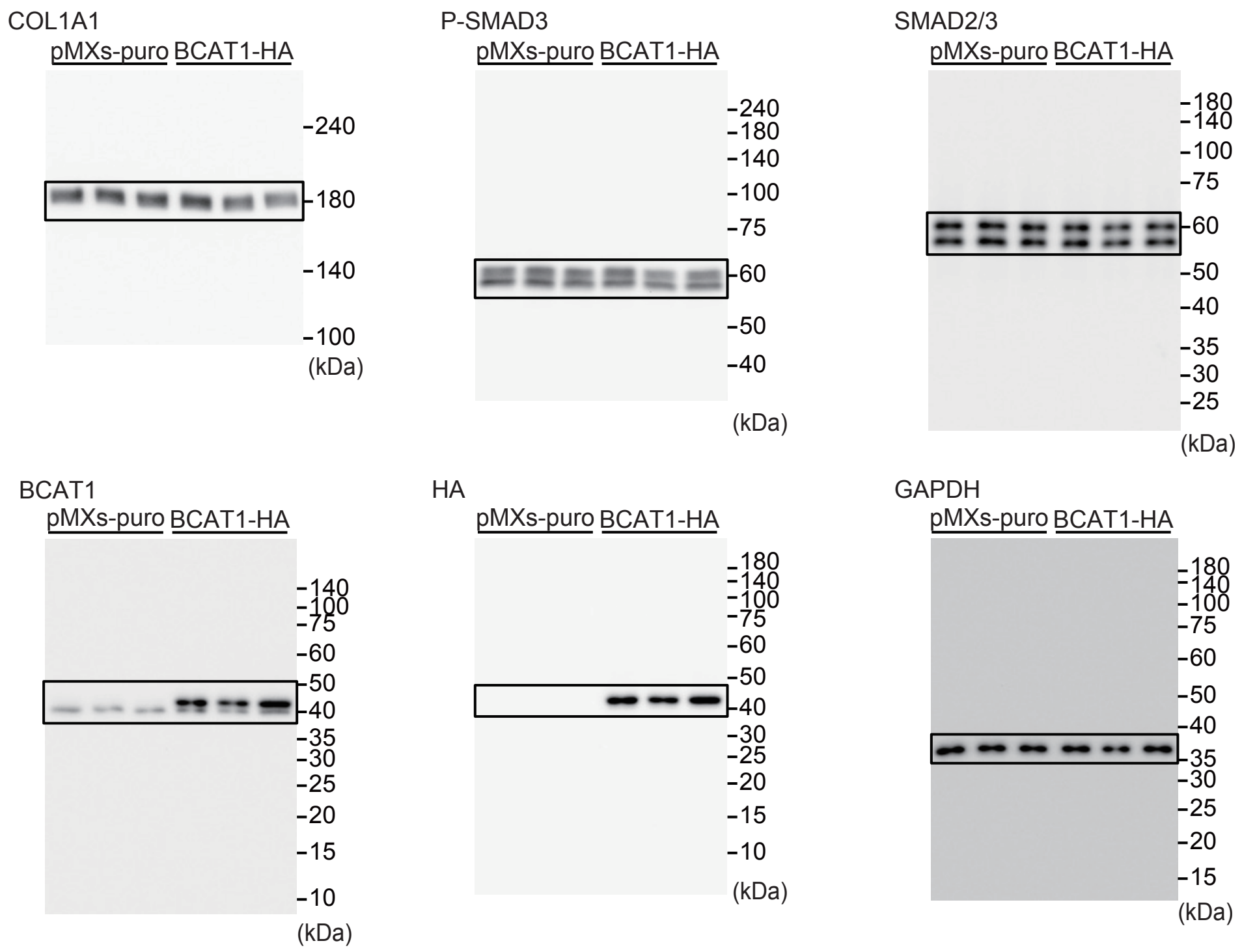

Supplemental Figure 5

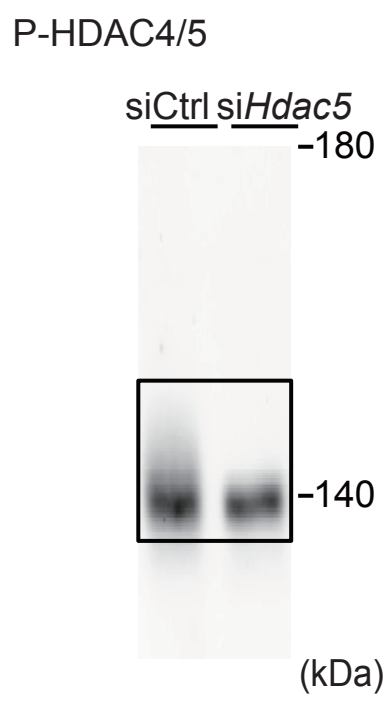

Supplemental Figure 6

C

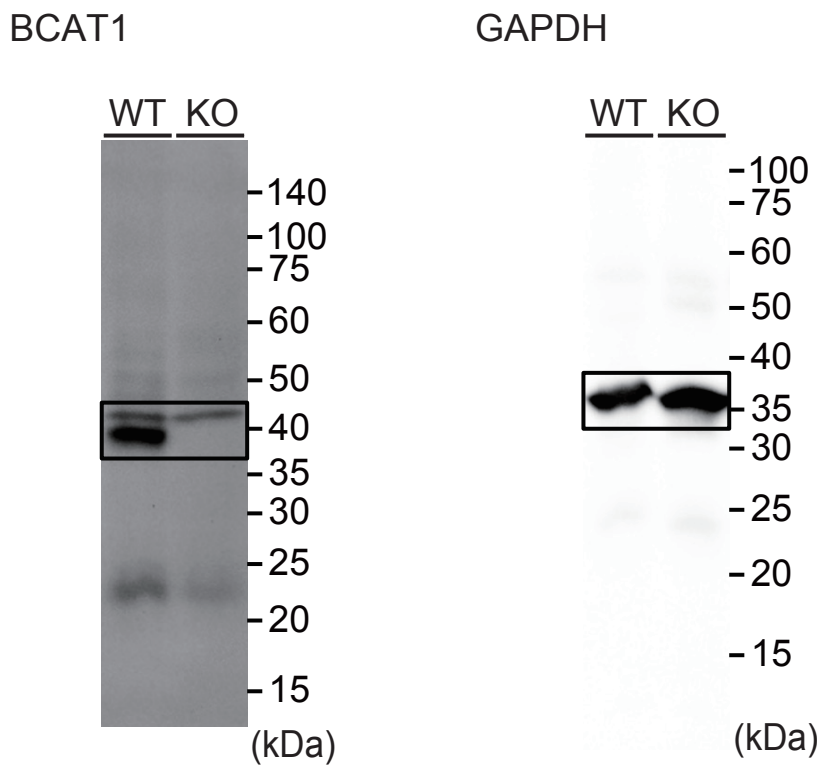

D

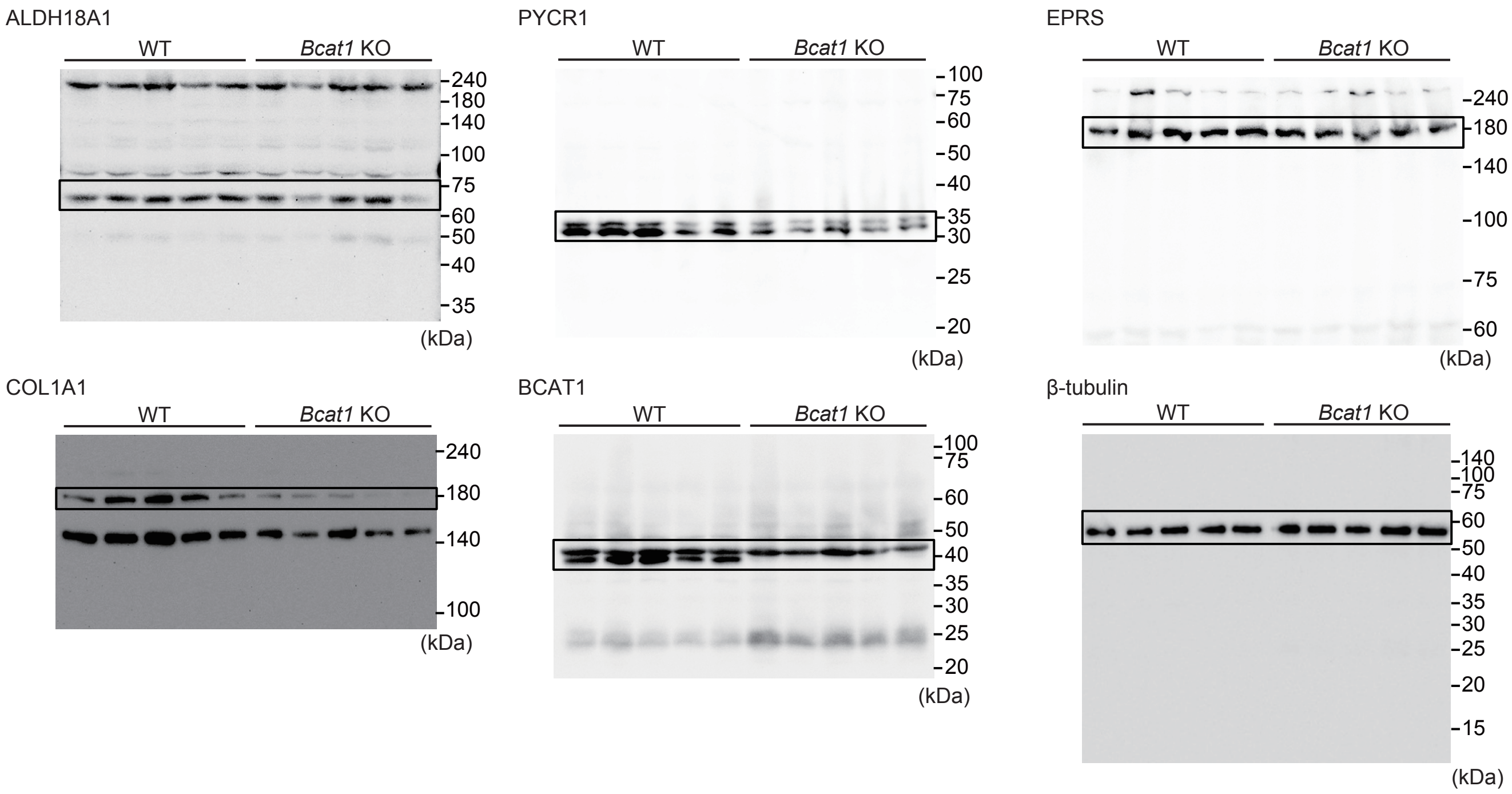

E

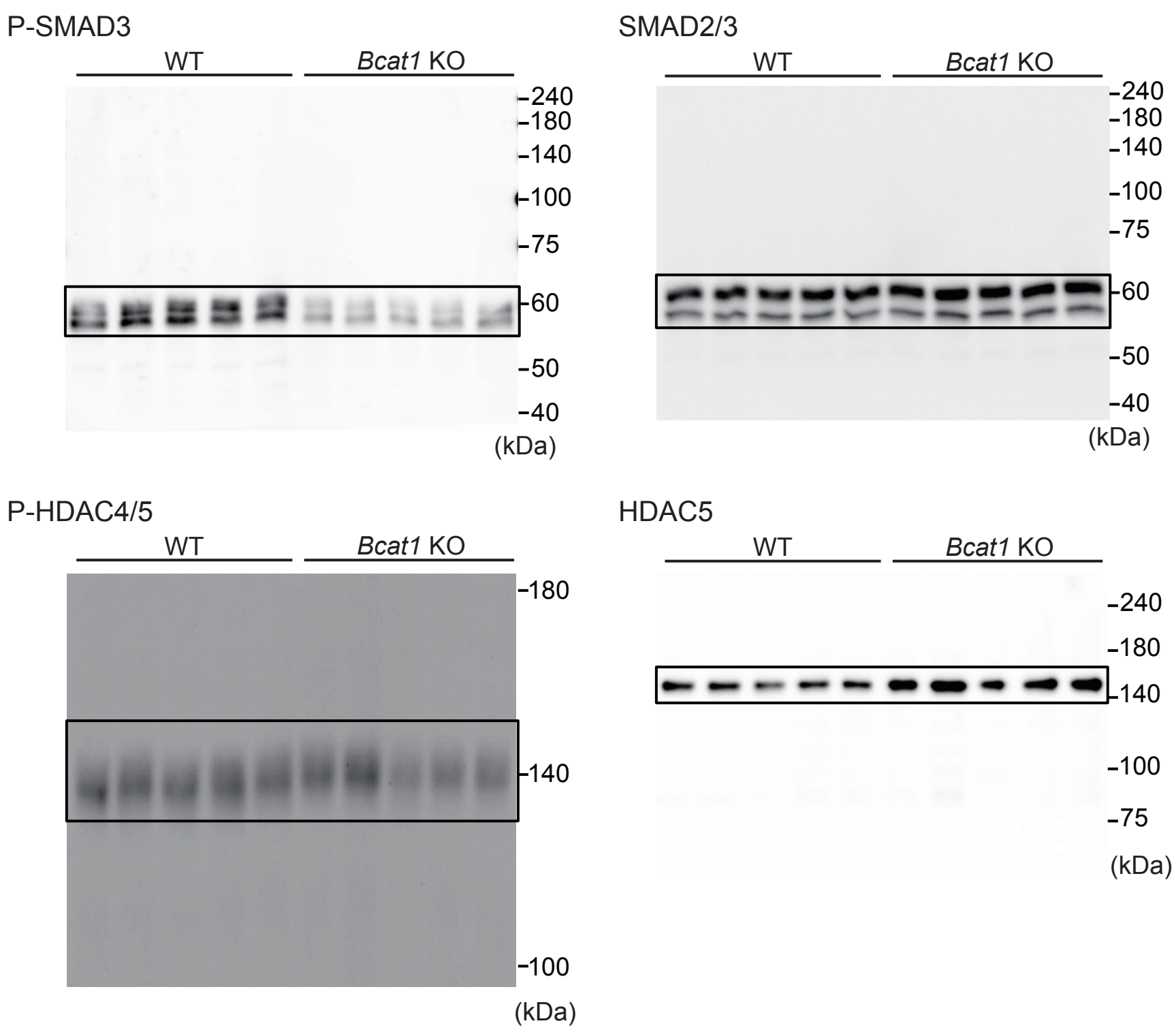

Supplemental Figure 7
